# Analysis of Antimicrobial Resistance and Virulence Factors in Multidrug-Resistant *Streptococcus suis* Serotype 2 Isolates Using Whole-Genome Sequencing

**DOI:** 10.3390/microorganisms13112552

**Published:** 2025-11-07

**Authors:** Lingling Zhang, Minglu Wang, Jiale Sheng, Lumin Yu, Yike Zhao, Wei Liao, Zitong Liu, Jiang Yu, Xinglin Zhang

**Affiliations:** 1 College of Agriculture and Forestry, Linyi University, Linyi 276005, China; zhangll0805@163.com (L.Z.); wangml040401@163.com (M.W.); 15275292005@163.com (J.S.); yulumin@lyu.edu.cn (L.Y.); zhaoyk19709873633@163.com (Y.Z.); 17379771965@163.com (W.L.); zhanglingling2020@lyu.edu.cn (Z.L.); 2 Key Laboratory of Livestock and Poultry Multi-omics of MARA, Institute of Animal Science and Veterinary Medicine, Shandong Academy of Agricultural Sciences, Jinan 250100, China

**Keywords:** MDR *Streptococcus suis* serotype 2, whole-genome sequencing, antimicrobial resistance, virulence factors

## Abstract

Multidrug-resistant (MDR) *Streptococcus suis* (*S. suis*) is a zoonotic pathogen capable of infecting pigs across all age groups, leading to conditions such as meningitis, arthritis, and endocarditis. In humans, infections can result in septic arthritis, meningitis, necrotizing fasciitis, and septicemia, which may be fatal. The absence of a complete genome sequence hinders comprehensive bioinformatic studies of MDR *S. suis* derived from pigs. In this study, we present the whole-genome sequence of MDR *S. suis* serotype 2 ST01 isolated from joint fluid samples obtained from pigs. Whole-genome analysis revealed that the ST01 chromosome carries 19 antibiotic resistance genes that confer resistance to major classes of antibiotic including aminoglycosides, tetracyclines, fluoroquinolones, lincosamides, polypeptide, and nitrofurans. Additionally, it contains 15 virulence factors associated with immune modulation, bacterial adherence, and stress survival. Whole-genome analysis identified 84 horizontal gene transfer elements in ST01 (comprising 28 genomic islands, 52 transposons, and 4 prophages), alongside mutations resulting in reduced virulence (302 instances) and loss of pathogenicity (34 instances). Furthermore, 18 antibiotic targets along with 21 lethal mutations were identified as potential targets for preventing, controlling, and treating infection caused by MDR *S. suis* serotype 2 ST01. In vivo infection experiments demonstrated that intraperitoneal inoculation with ST01 resulted in mortality among Kunming mice, with a median lethal dose (LD_50_) of 5.62 × 10^9^ CFU/mL. Histopathological analysis revealed varying degrees of lesions in the infected organs of the mice. This study thus provides valuable insights into strategies aimed at combating *S. suis* infections and their transmission within swine populations.

## 1. Introduction

*Streptococcus suis* (*S. suis*) is a facultatively anaerobic, Gram-positive coccus that typically forms chains. It is non-flagellated, non-motile, and asporogenous; however, it possesses a capsule [1]. This bacterium represents a significant zoonotic pathogen that commonly colonizes the respiratory, digestive, and reproductive tracts of pigs, particularly in the tonsils and nasal cavity. *S. suis* can cause severe infections in swine, including arthritis, septicemia, meningitis, and other systemic diseases. In humans, exposure to *S. suis* may lead to bacterial meningitis and toxic shock syndrome (TSS) in susceptible individuals [2,3]. *S. suis* exhibits considerable serotypic diversity; current classification recognizes 29 traditional serotypes based on distinct capsular polysaccharide antigens alongside advancing molecular biological techniques [3]. Among these serotypes, serotype 2 of *S. suis* remains the predominant strain isolated from human clinical cases worldwide. Notably, two large-scale outbreaks of human infections caused by *S. suis* serotype 2 occurred in China in 1998 and 2005, resulting in 14 and 38 fatalities, respectively [4,5]. Consequently, *S. suis* has garnered global attention from medical professionals as a significant public health threat.

The ability of *S. suis* to withstand various environmental stresses and flourish within host organisms can be attributed to its diverse array of virulence factors. These include capsular polysaccharide, suilysin, enolase, extracellular factor, muramidase-released protein, fibrinogen-binding proteins, peptidoglycan, lipoteichoic acid, glutamate dehydrogenase, a two-component signaling or regulatory system, hyaluronidase, subtilisin-like protease, and IgG-binding protein, among others. Collectively, these factors facilitate survival through multiple mechanisms such as physical protection, nutrient acquisition, adhesion–invasion processes, and immune evasion strategies [6,7,8,9].

Currently, antibiotic therapy and vaccine-based prevention are the primary strategies for managing *S. suis* infections. However, *S. suis* exhibits significant serotype diversity; even within the same serotype, different strains show considerable genetic, phenotypic, and geographical variation [10]. Consequently, existing vaccines provide suboptimal protection against *S. suis* [11]. Furthermore, the persistent overuse and misuse of antibiotics have led to the development of various resistance mechanisms in *S. suis*, including the production of degradative enzymes, plasmid/transposon-mediated dissemination of antibiotic resistance genes, reduced bacterial membrane permeability, alterations in antibiotic targets, overexpression of efflux pumps, and biofilm formation [12,13,14,15]. Research has shown that *S. suis* has high resistance rates to multiple clinically effective antibiotics [13,16,17]. As a result, these strains are categorized into three groups: multidrug-resistant (MDR) strains, extensively drug-resistant (XDR) strains, and even pan-drug-resistant (PDR) strains—each posing significant challenges to clinical treatment.

The economic impact of *S. suis* on the swine industry, along with its public health implications, underscores the urgent need for robust surveillance strategies. A significant challenge in managing *S. suis* is its escalating antimicrobial resistance (AMR), with high rates of resistance to tetracyclines, macrolides, and lincosamides reported, leading to MDR phenotypes that complicate treatment regimens [18,19]. Conventional methods for AMR profiling and epidemiological tracking, such as phenotypic susceptibility testing and serotyping, offer limited resolution for understanding the genetic diversity and dissemination dynamics of resistant clones. Whole-genome sequencing (WGS), as a high-resolution genetic analysis tool, has been increasingly utilized in bacterial AMR studies. The advent of WGS has revolutionized bacterial surveillance by providing high-resolution insights into the genomic basis of resistance and population structure. WGS enables the comprehensive identification of resistance genes, point mutations in target genes (e.g., *gyrA*, *parC* for fluoroquinolones; *pbp* genes for β-lactams), and the mobile genetic elements (MGEs) that facilitate their spread, such as integrative conjugative elements (ICEs) and prophages [19,20,21]. For example, studies have identified ICEs carrying *tet(O)* and *erm(B)* genes, conferring tetracycline and macrolide resistance, respectively, in *S. suis* serotype 14 strains [20]. Alarmingly, WGS has also revealed strains harboring transferable resistance genes to last-resort antibiotics like linezolid (*optrA*) and vancomycin (*vanG*), highlighting the role of *S. suis* as a reservoir for AMR gene dissemination among Gram-positive bacteria [21,22]. In epidemiological investigations, WGS facilitates precise molecular typing (e.g., Multi-Locus Sequence Typing (MLST)), serotype prediction from capsule loci, and phylogenetic analysis based on single-nucleotide polymorphisms (SNPs), allowing researchers to trace outbreak origins, monitor cross-species transmission, and identify emerging virulent clones. For instance, WGS-based surveillance in Spain and Switzerland has revealed the dominance and dissemination of specific sequence types (e.g., ST1, ST7) and their association with resistance patterns [18,20]. International standards, such as ISO 23418:2022, now provide guidelines for applying WGS in the food chain, ensuring reproducibility and reliability in bacterial characterization [23]. Despite challenges in data standardization and analysis, WGS remains a powerful tool for One Health approaches, integrating data from animals, humans, and the environment to mitigate the AMR crisis [19,24]. Since the initial identification of *S. suis*, over 1600 human cases have been reported across more than 30 countries and regions [25]. Notably, approximately 80% of these infections are attributed to *streptococcus suis* serotype 2 (SS2), which is considered the most virulent serotype and has been extensively studied for its pathogenicity and prevalence [26,27,28]. However, there is a significant lack of research and data on the transmission dynamics of SS2 in food-producing animals, such as farmed pigs, and the evolution of antibiotic resistance. Further investigations are urgently needed to inform the development of effective vaccines against *S. suis* infections.

This study aims to investigate the whole-genome characteristics, pathogenicity, and antibiotic resistance of an SS2 isolate obtained from the synovial fluid of a deceased piglet. Furthermore, it seeks to elucidate the pathogenic mechanisms and antibiotic resistance profiles of this SS2 isolate in a mouse model, as well as the differences compared to SS2 isolates from other sources. Thus, this research contributes to the development of strategies for the prevention, control, and treatment of SS2 infections.

## 2. Materials and Methods

### 2.1. Isolation and Identification of SS2

An outbreak of a bacterial disease occurred at a swine farm in Shandong Province, China. The affected piglets exhibited clinical signs including depression, lethargy, loss of appetite, and convulsions before succumbing. Postmortem examinations revealed joint effusion, gelatinous pericardial exudate, fibrinous peritoneal exudate, and splenomegaly. To isolate the bacteria, viscera and joint fluid from deceased piglets (Taian, China) were collected. The organs were homogenized using a stomacher (Scientz, Ningbo, China) for 2 min. The resulting homogenate and joint fluid were aseptically streaked onto blood agar plates and Tryptone Soy Agar (TSA) (Qingdao Hi-Tech Industrial Park Hope Bio-Technology Co., Ltd., Qingdao, China) plates containing 5% fetal bovine serum (Zhejiang Tianhang Biotechology Co., Ltd., Huzhou, China). All plates were incubated at 37 °C for 48 h [27]. Suspected colonies were subsequently inoculated into Trypticase Soy Broth (TSB) liquid medium supplemented provided by Qingdao Hi-Tech Industrial Park Hope Bio-Technology Co., Ltd. with 5% fetal bovine serum and cultured in a shaking incubator (Shanghai Minquan Instrument Co., Ltd., Shanghai, China) at 37 °C for 16–18 h. Gram staining and biochemical tests (Hangzhou Microbial Reagent Co., Ltd., Hangzhou, China) were conducted for further confirmation. PCR amplification was conducted using *S. suis* specific primers [29] (F: 5′-TTCTGCAGCGTATTCTGTCAAACG-3′; R: 5′-TGTTCCATGGACAGATAAAGATGG-3′) and SS2 specific primers (F: 5′-TGAGTGATTTGTCGGGAGGG-3′; R: 5′-GAGTATCTAAAGAATGCCTATTG-3′). The identified SS2 strain was designated ST01 and preserved in 50% sterile glycerol at −80 °C. Before each experiment, a pure culture of the SS2 strain ST01 was prepared by inoculating TSA plates supplemented with 5% fetal bovine serum and incubating overnight at 37 °C.

### 2.2. Whole-Genome Sequencing and Library Construction

The genomic DNA of ST01 was extracted using the TIANamp Bacteria DNA kit (Tiangen Biotech (Beijing) Co., Ltd., Beijing, China) following the manufacturer’s instructions. The quality, purity, integrity, and yield of the extracted genomic DNA were assessed through 0.8% agarose gel electrophoresis and a NanoDrop2000 spectrophotometer (Thermo Fisher Scientific, Pittsburg, PA, USA) and quantified using a Qubit 4.0 fluorometer (Invitrogen, Carlsbad, CA, USA). Whole-genome sequencing was conducted on both the Illumina NovaSeq X Plus platform and the Oxford Nanopore ONT (Version R10) platform. Libraries for sequencing were prepared using the TruSeqTM DNA Sample Prep Kit (Illumina, San Diego, CA, USA) specifically designed for Illumina TruSeq Nano DNA LT and quantified again with a Qubit 4.0 fluorometer (Invitrogen) [30]. All procedures mentioned above were carried out at Shanghai Personal Biotechnology Co. Ltd. (Shanghai, China). Following the completion of genome assembly, the whole-genome sequence of ST01 was compared with that of *S. suis* published in the NCBI Genbank nucleotide sequence database (https://www.ncbi.nlm.nih.gov/) accessed on 20 March 2024.

### 2.3. Genome Functional Elements Analysis

Genome functional element analysis of ST01 was conducted to predict repetitive sequences, clustered regularly interspaced short palindromic repeats (CRISPRs), noncoding RNAs (ncRNAs), and protein-coding genes. Interspersed repeats were identified through comparison with the Repbase database (https://www.girinst.org/repbase/) accessed on 16 May 2024. CRISPRs were predicted using the CRISPR finder tool (http://crispr.i2bc.paris-saclay.fr/Server/) accessed on 16 May 2024. ncRNAs include small RNAs (sRNAs), ribosome RNAs (rRNAs), transfer RNAs (tRNAs), small nuclear RNAs (snRNAs), small nucleolar RNAs (snoRNAs), and microRNAs (miRNAs). tRNAs were predicted utilizing tRNAscan-SE software (Version 2.0) (http://lowelab.ucsc.edu/tRNAscan-SE/) accessed on 16 May 2024, while rRNAs were predicted using Barrnap software (Version 0.9) (http://www.vicbioinformatics.com/software.barrnap.shtml) accessed on 16 May 2024. Predictions for other ncRNAs were obtained by comparing against the Rfam database (http://rfam.xfam.org/) accessed on 16 May 2024. Protein-coding genes were predicted using GeneMarkS software (Version 4.32) (http://topaz.gatech.edu/GeneMark/) accessed on 16 May 2024.

### 2.4. Genome Subsystem Analysis

Genome subsystem analysis of ST01 was conducted to predict virulence factors of pathogenic bacteria using the Virulence Factor Database (VFDB) (http://www.mgc.ac.cn/VFs/main.htm) accessed on 16 May 2024, assess comprehensive antibiotic resistance through the Comprehensive Antibiotic Resistance Database (CARD) (https://card.mcmaster.ca/) accessed on 16 May 2024, and examine carbohydrate-active enzymes via the Carbohydrate-Active Enzymes (CAZy) database (http://www.cazy.org/) accessed on 16 May 2024. The prophages present in the genome were predicted using the Phispy tool (Version 2.2) [31], while genomic islands (GIs) were identified using the IslandViewer 4 database (http://www.pathogenomics.sfu.ca/islandviewer/) accessed on 16 May 2024. Analyses for VFDB and CARD were performed using the BLAST tool (Version 2.5.0) (https://blast.ncbi.nlm.nih.gov/Blast.cgi) accessed on 16 May 2024, whereas CAZy was analyzed using hmmscan software (Version 3.2.1) (http://hmmer.org/) accessed on 16 May 2024.

### 2.5. Genome Functional Annotation Analysis

The functions of protein-coding genes in ST01 were investigated through various databases, including the Non-redundant Protein database (NR, https://ftp.ncbi.nih.gov/blast/db/) accessed on 16 May 2024, Evolutionary Genealogy of Genes: Non-supervised Orthologous Groups databases (eggNOG, http://eggnogdb.embl.de) accessed on 16 May 2024, Kyoto Encyclopedia of Genes and Genomes (KEGG, http://www.genome.jp/kegg/) accessed on 16 May 2024, Swiss-Prot databases (http://www.expasy.ch/sprot accessed on 16 May 2024), Gene Ontology databases (GO, http://www.geneontology.org/) accessed on 16 May 2024, Transporter Classification Database (TCDB, http://www.tcdb.org/) accessed on 16 May 2024, and the Pathogen–Host Interactions Database (PHI, http://www.phi-base.org/) accessed on 16 May 2024. Additionally, proteases—enzymes that catalyze the hydrolysis of peptide bonds between amino acids within proteins or peptides—were predicted using the MEROPS database (http://www.ebi.ac.uk/merops) accessed on 16 May 2024. The structural domains of proteins were analyzed with reference to the Pfam database (http://pfam.xfam.org/) accessed on 16 May 2024.

### 2.6. Antimicrobial Susceptibility Assessment

In accordance with the guidelines established by the Clinical and Laboratory Standards Institute (CLSI, 2022) [32] and the antibacterial range standard book provided by Hangzhou Microbial Reagent Co., Ltd. (Hanzhou, China), the antimicrobial susceptibility profile of ST01 was evaluated using the Kirby–Bauer disk diffusion method on Mueller–Hinton agar (Haibo, Qingdao, China). In this susceptibility determination, a total of 11 categories (28 types) of antimicrobial susceptibility disk were utilized, including cefoperazone (75 µg), ceftriaxone (30 µg), ceftazidime (30 µg), ampicillin (10 µg), oxacillin (1 µg), penicillin G (10 µg), piperacillin (100 µg), cephradine (30 µg), cefazolin (30 µg), cefalexin (30 µg), neomycin (30 µg), kanamycin (30 µg), gentamicin (10 µg), amikacin (30 µg), minocycline (30 µg), doxycycline (30 µg), tetracycline (30 µg), ofloxacin (5 µg), ciprofloxacin (5 µg), norfloxacin (10 µg), erythromycin (15 µg), midecamycin (30 µg), clindamycin (2 µg), chloramphenicol (30 µg), vancomycin (30 µg), cotrimoxazole (23.75/1.25 µg), polymyxin B (300 µg), and furazolidone (100 µg). Specifically, when the diameter of the inhibition zone is greater than or equal to the sensitive inflection point, it indicates that SS2 strain ST01 is sensitive to that antibiotic. Conversely, when this diameter is less than or equal to the resistance inflection point, it signifies that ST01 is resistant. If it falls between these two points, then it can be concluded that there is intermediate sensitivity present for that particular antibiotic against SS2 strain ST01.

### 2.7. Animals

A total of 60 clean-grade Kunming mice, with an equal sex distribution (30 males and 30 females), aged 6 weeks and weighing approximately 30 g each, were obtained from Qingdao Daren Fucheng Animal Husbandry Co., Ltd. (Qingdao, China) (License No. SYXK (Lu) 20180027). The mice were provided with sufficient food and water (a complete diet free from antibiotics) and housed in uniform and comfortable environmental conditions. Following a feeding period of 7 days, the mice were utilized for the infection experiment. At the experiment’s conclusion, all mice were humanely euthanized via intraperitoneal injection of an overdose of pentobarbital sodium. All procedures strictly adhered to the Guide for the Care and Use of Laboratory Animals established by the National Institutes of Health (NIH) and the American Veterinary Medical Association (AVMA) guidelines on euthanasia [33,34]. The study protocol was approved by the Animal Ethics Committee of Linyi University (Approval No. LYU20250114).

### 2.8. Animal Infection Experiments

After 7 days of feeding, all mice underwent an animal infection experiment to assess the virulence of the ST01 strain. The mice were randomly assigned to six groups (groups A–F), with each group consisting of 5 male and 5 female mice. First, the bacterial lawns of ST01 cultured overnight on a TSA plate supplemented with 5% fetal bovine serum were collected and rinsed three times with physiological saline, and then resuspended to prepare inocula at concentrations of 1.0 × 10^10^, 1.0 × 10^9^, 1.0 × 10^8^, 1.0 × 10^7^, and 1.0 × 10^6^ CFU/mL, respectively [35]. Subsequently, group A received an intraperitoneal injection of 0.2 mL of 1.0 × 10^10^ CFU/mL bacterial suspension, group B received 0.2 mL of 1.0 × 10^9^ CFU/mL, group C received 0.2 mL of 1.0 × 10^8^ CFU/mL, group D received 0.2 mL of 1.0 × 10^7^ CFU/mL, and group E received 0.2 mL of 1.0 × 10^6^ CFU/mL. Group F was injected intraperitoneally with 0.2 mL of physiological saline as a negative control. Following inoculation, the physiological activity and clinical symptoms of the mice were closely monitored, and survival curves were recorded for virulence assessment. After 7 days, all mice were euthanized via intraperitoneal administration of an overdose of pentobarbital sodium [36]. One mouse each from group B and group F was randomly selected for necropsy. Heart, liver, spleen, lungs, kidneys, and brain tissues were collected for histopathological examination. The tissues were fixed in 4% paraformaldehyde, stained with Hematoxylin–Eosin (HE) to create pathological sections, and observed under a microscope [37].

### 2.9. Statistical Analysis

The GraphPad Prism 6.02 (GraphPad, San Diego, CA, USA) and IBM SPSS statistics 25.0 (IBM, Armonk, NY, USA) were used for statistical analysis. The test results were shown as means ± SD.

## 3. Results

### 3.1. Identification of SS2

**Figure 1:**
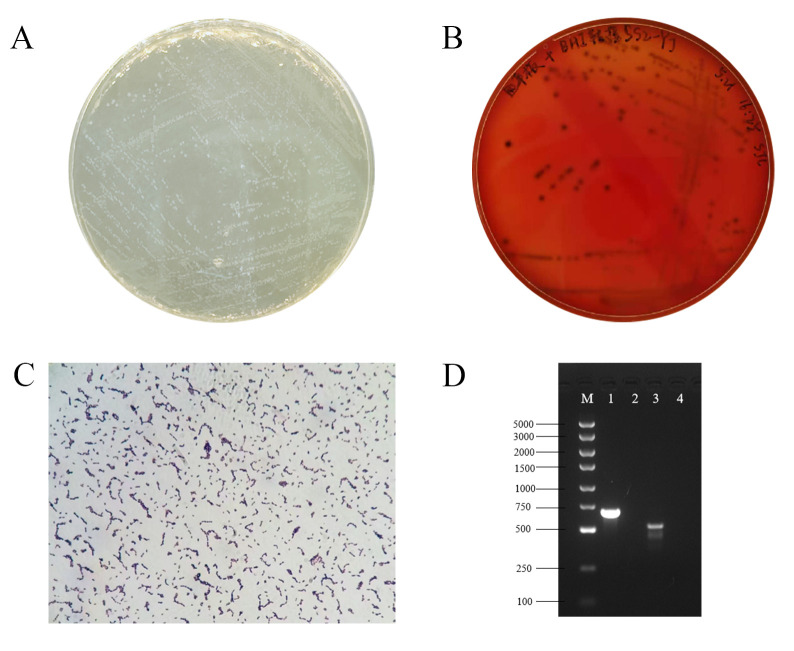
Isolation and identification of the SS2 strain ST01. (**A**) Colony morphology of the *S. suis* strain ST01 on TSA plate supplemented with 5% fetal bovine serum; (**B**) hemolysis analysis of the *S. suis* strain ST01 on blood agar plate (note: the non-English in this figure are marked as experimental records); (**C**) Gram staining of the *S. suis* strain ST01 (1000×); (**D**) PCR amplification results for SS2 strain ST01 (M, DL5000 DNA Marker; 1, Amplification results for *S. suis*; 3, Amplification results for SS2; 2&4, Negative control).

*S. suis* strain ST01, isolated from the joint fluid of a deceased piglet, formed grayish-white, moist, smooth, circular, or ovoid colonies with entire margins on TSA plates supplemented with 5% fetal bovine serum (Figure 1A). On blood agar plates, the colonies were surrounded by a zone of hemolysis characteristic of alpha-hemolysis (Figure 1B), indicating alpha-hemolytic activity. Gram staining revealed Gram-positive coccoid cells arranged in pairs or short chains (Figure 1C), morphological features typical of *S. suis*. Biochemical profiling showed that the isolate fermented esculin, raffinose, lactose, mannose, mannitol, and inulin, but did not ferment sorbitol, arabinose, melezitose, D-ribose, or sodium hippurate. In addition, the strain did not grow in 6.5% NaCl broth and pH 9.6 broth (Table 1), further supporting its classification within *S. suis*. PCR amplification using specific primers for *S. suis* and SS2-specific primers generated amplicons of approximately 695 bp and 557 bp, respectively (Figure 1D), confirming the identity of the isolate as SS2. Based on these findings, the isolated strain was identified as SS2 and named ST01.

### 3.2. Quality Assessment of Genome Assemblies

**Figure 2:**
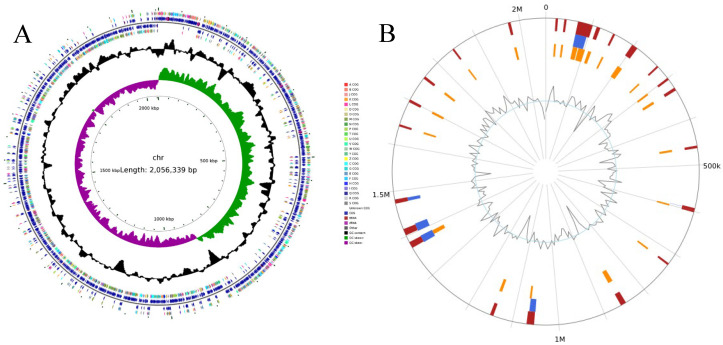
An overview of the complete genome of SS2 strain ST01. (**A**) Chromosomal features, from the innermost to the outermost circle: the first circle represents the genomic scale; the second circle is GC skew; the third circle shows GC content; the fourth and seventh circles represent the COG to which each CDS belongs; the fifth circle illustrate the genomic positions of CDS; and the sixth circle is the positions of tRNA and rRNA on the genome. (**B**) Genomic islands (GIs) identified on the SS2 strain ST01 chromosome. Blue regions indicate predictions made by IslandPath-DIMOB; yellow regions represent predictions from SIGI-HMM; red regions denote genomic islands predicted by both methods.

The sequence obtained from sequencing ST01 was compared in the NCBI database, and the results indicated that ST01’s sequence exhibited the highest similarity to that of *S. suis*, with an identification rate of 99.86% (Supplementary Table S1). Currently, bacteriologists generally agree that when the 16S rDNA sequence homology exceeds 97%, the organisms can be considered the same species within the genus [38]. Consequently, it was reaffirmed that the ST01 isolate is indeed an *S. suis.* Genome assembly quality assessment indicated that the SS2 strain ST01 comprises one chromosome (2,056,339 bp; GC content 41.30%) with no detectable plasmids, as illustrated in Figure 2A and detailed in Table 2. The Phispy tool was employed to identify prophages within the ST01 genome. The results indicated that four prophages were predicted within its chromosomal sequence (Table 2). Additionally, a comparison with the IslandViewer 4 database revealed a total of 28 genomic islands present on the chromosome (Figure 2B).

### 3.3. Genome Functional Elements Analysis Results

The protein-coding genes of ST01 were predicted using GeneMarkS software (Version 4.3.2), revealing a total of 1973 coding sequences located on the chromosome. The chromosome also contained 56 tRNAs, 12 rRNAs, and 34 ncRNAs. Additionally, there were two CRISPRs and four prophages present on the chromosome. Furthermore, the interspersed repeats of ST01 on the chromosome included six SINEs (short interspersed repeats), 35 LINEs (long interspersed repeats), 111 LTRs (long terminal repeats), 52 transposons, five unclassified interspersed repeats, and six satellites RNAs (Table 2).

### 3.4. Genome Subsystem Analysis Results

**Figure 3:**
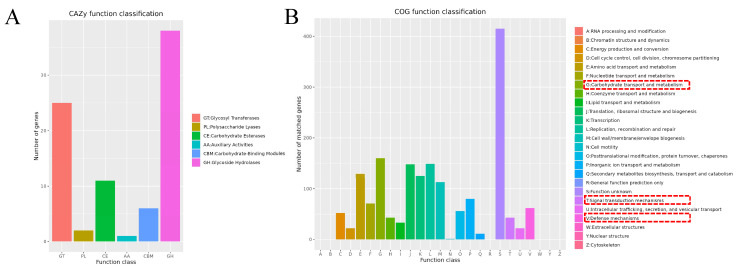
(**A**) The functional classifications annotated using CAZy on the chromosome of the SS2 strain ST01. (**B**) The functional classifications annotated using COG on the chromosome of the SS2 strain ST01.

Protein-coding sequences from the ST01 genome were aligned against amino acid sequences in the VFDB database using BLAST analysis to predict virulence factor-associated genes. This analysis revealed that all 15 predicted virulence factors were associated with bacterial adherence, immune modulation, and stress survival (Table 3). BLAST analysis against the CARD database identified a total of 19 antibiotic resistance genes, 18 antibiotic target genes, and one antibiotic biosynthesis gene within the chromosome (Table 4). Moreover, a comprehensive annotation of carbohydrate-active enzymes in the ST01 genome was performed using the CAZy database (Figure 3A and Table S2), revealing a total of 83 such enzymes on the chromosome, including 25 glycosyl transferases (GTs), 2 polysaccharide lyases (PLs), 11 carbohydrate esterases (CEs), 1 auxiliary activity enzyme (AA), 6 carbohydrate-binding modules (CBMs), and 38 glycoside hydrolases (GHs).

### 3.5. Genome Functional Annotation Analysis Results

Protein-coding genes were annotated using multiple databases. The results indicated that annotation with the NR database identified a total of 1969 protein-coding genes located on the chromosome. Comparative analysis utilizing the eggNOG database annotated an additional 1735 chromosomal genes. Figure 3B illustrates the functional classification within eggNOG, revealing that 113 chromosomal genes are associated with cell wall/membrane/envelope biogenesis, 43 with signal transduction mechanisms, and 62 with defense mechanisms.

**Figure 4:**
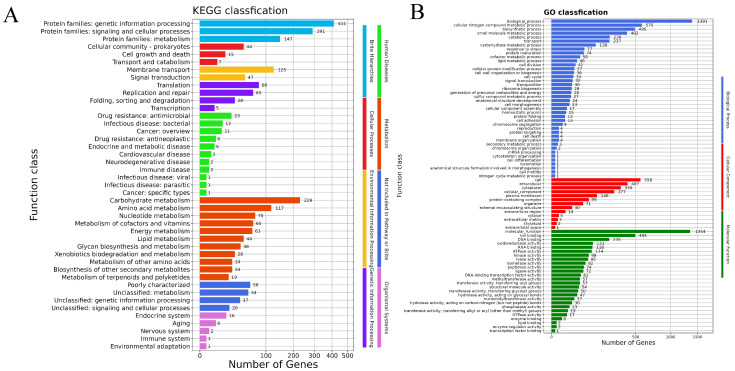
(**A**) The functional classifications annotated using KEGG on the Chromosome of SS2 strain ST01. (**B**) The functional classifications annotated using GO on the Chromosome of SS2 strain ST01.

In the ST01 genome, protein-coding genes were annotated and categorized into eight KEGG categories through KO (KEGG Ortholog) annotation and KEGG pathway analysis. As depicted in Figure 4A, a total of 43 chromosomal pathways were classified into the following eight KEGG categories: Brite hierarchies (3), Cellular processes (3), Environmental information processing (2), Human diseases (11), Genetic information processing (4), Metabolism (11), Not included in pathway or Brite (4), and Organismal systems (5).

The Gene Ontology (GO) system provides a comprehensive functional classification of gene properties and their products [39]. In this study, GO classification assigned a total of 106 chromosomal terms to three primary ontologies: biological processes (58 terms), cellular components (17 terms), and molecular functions (31 terms) as illustrated in Figure 4B. Specific GO term annotations detailed in Figure 4B include stress response encompassing 77 protein-coding genes, cell wall organization or biogenesis involving 36 protein-coding genes, and cell adhesion comprising 13 protein-coding genes, among others.

**Figure 5:**
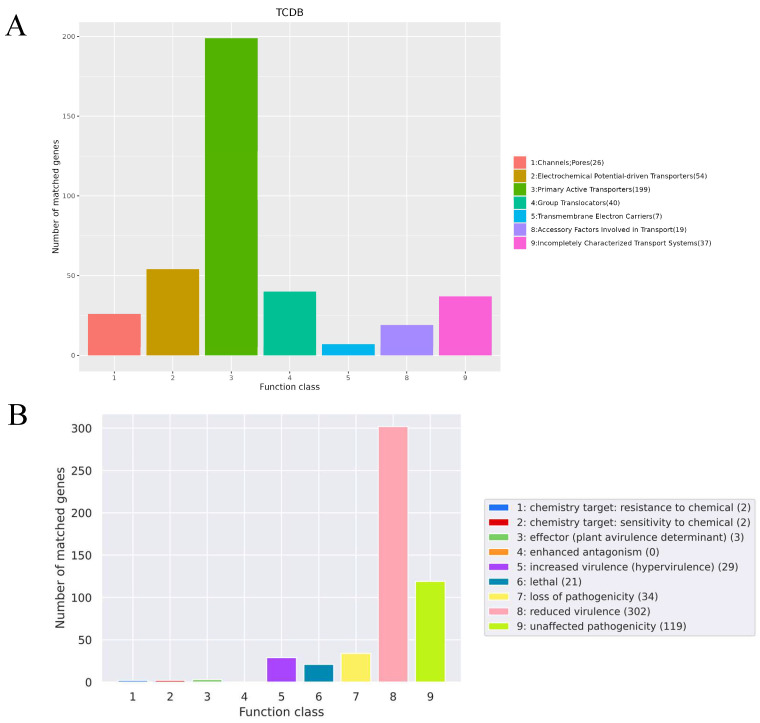
(**A**) The functional classifications annotated using TCDB on the SS2 strain ST01 Chromosome. (**B**) The functional classifications annotated using PHI on the SS2 strain ST01 Chromosome.

A comparative analysis utilizing data from the TCDB Database revealed a total of 382 transport proteins presented on the chromosome. These proteins can be categorized as follows: 26 channels or pores, 54 electrochemical potential-driven transporters, 199 primary active transporters, 40 group translocators, seven transmembrane electron carriers, 19 accessory factors involved in transport, and 37 incompletely characterized transport systems (Figure 5A).

The annotation of bacterial virulence and pathogenicity was conducted using the PHI database. This analysis uncovered distinct categories related to chromosomal mutations: specifically, four associated with chemical targets, 29 linked to enhanced virulence, 302 resulting in diminished virulence, 34 leading to loss of pathogenicity, and 21 lethal mutations that resulted in mortality within strain ST01 (Figure 5B and Table S3). Furthermore, using data from the Pfam database, predictions were made regarding 3358 structural domains distributed across 1646 proteins within genome ST01. Concurrently, analysis with the MEROPS database identified 763 peptidases among 695 proteins, which constitutes approximately 35.22% of all protein-coding genes (Tables S4 and S5).

### 3.6. Antimicrobial Susceptibility Analysis

The antimicrobial susceptibility profile of strain ST01 against 28 tested agents is presented in Table 5. Strain ST01 exhibited resistance to neomycin, kanamycin, gentamicin, amikacin, minocycline, tetracycline, ciprofloxacin, clindamycin, polymyxin B, and furazolidone. Intermediate susceptibility was noted for oxacillin, penicillin G, doxycycline, and norfloxacin. Conversely, it demonstrated sensitivity to cefoperazone, ceftriaxone, ceftazidime, ampicillin, piperacillin, cephradine, cefazolin, cefalexin, ofloxacin, erythromycin, midecamycin, chloramphenicol, vancomycin, and cotrimoxazole. Antibiogram analysis revealed that the SS2 strain ST01 is resistant to 6 out of 11 classes, which is 10 out of 28 antimicrobial agents (35.7%), thereby meeting the international standards for MDR bacterial pathogens [40]. Consequently, ST01 was classified as an MDR SS2 strain.

### 3.7. Animal Infection Analysis

**Figure 6:**
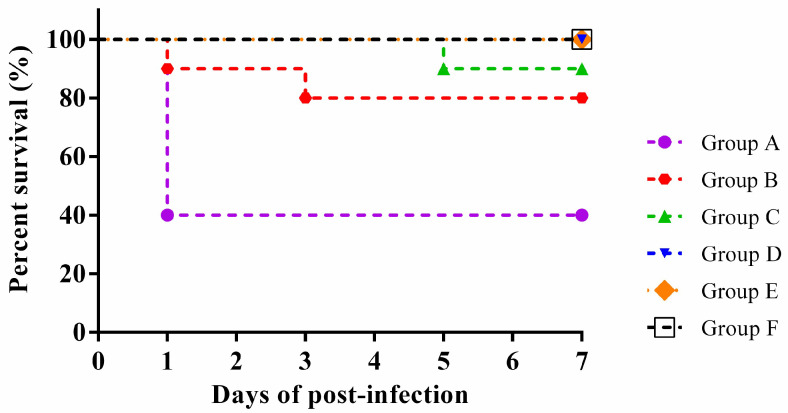
The survival rates of mice infected with varying concentrations of the SS2 strain ST01 were assessed through mouse infection experiments. Mice in experimental groups A–E were administered intraperitoneal injections of 0.2 mL of MDR SS2 strain ST01 at concentrations of 1.0 × 10^10^, 1.0 × 10^9^, 1.0 × 10^8^, 1.0 × 10^7^, and 1.0 × 10^6^ CFU/mL, respectively. Mice in group F received intraperitoneal injections of 0.2 mL of physiological saline. Survival was monitored for 7 days post-infection.

**Figure 7:**
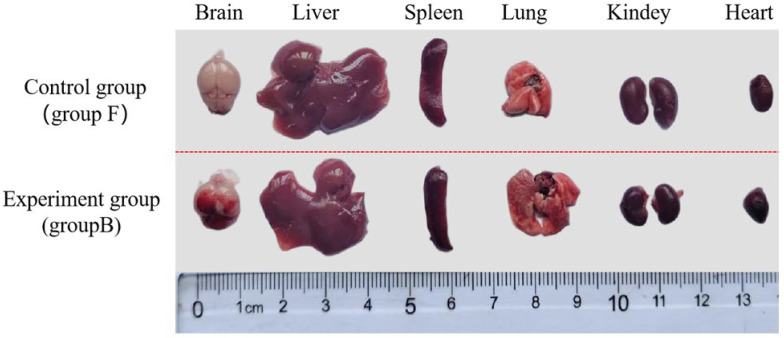
Pathological and anatomical examination of mouse organs. The control group (group F) comprised the heart, liver, spleen, lung, and kidney of a mouse that was randomly selected to receive an intraperitoneal injection of 0.2 mL of physiological saline. The experimental group (group B) consisted of the heart, liver, spleen, lung, and kidney of a mouse that was randomly selected and infected with the MDR SS2 strain ST01 at a concentration of 1.0 × 10^9^ CFU/mL.

**Figure 8:**
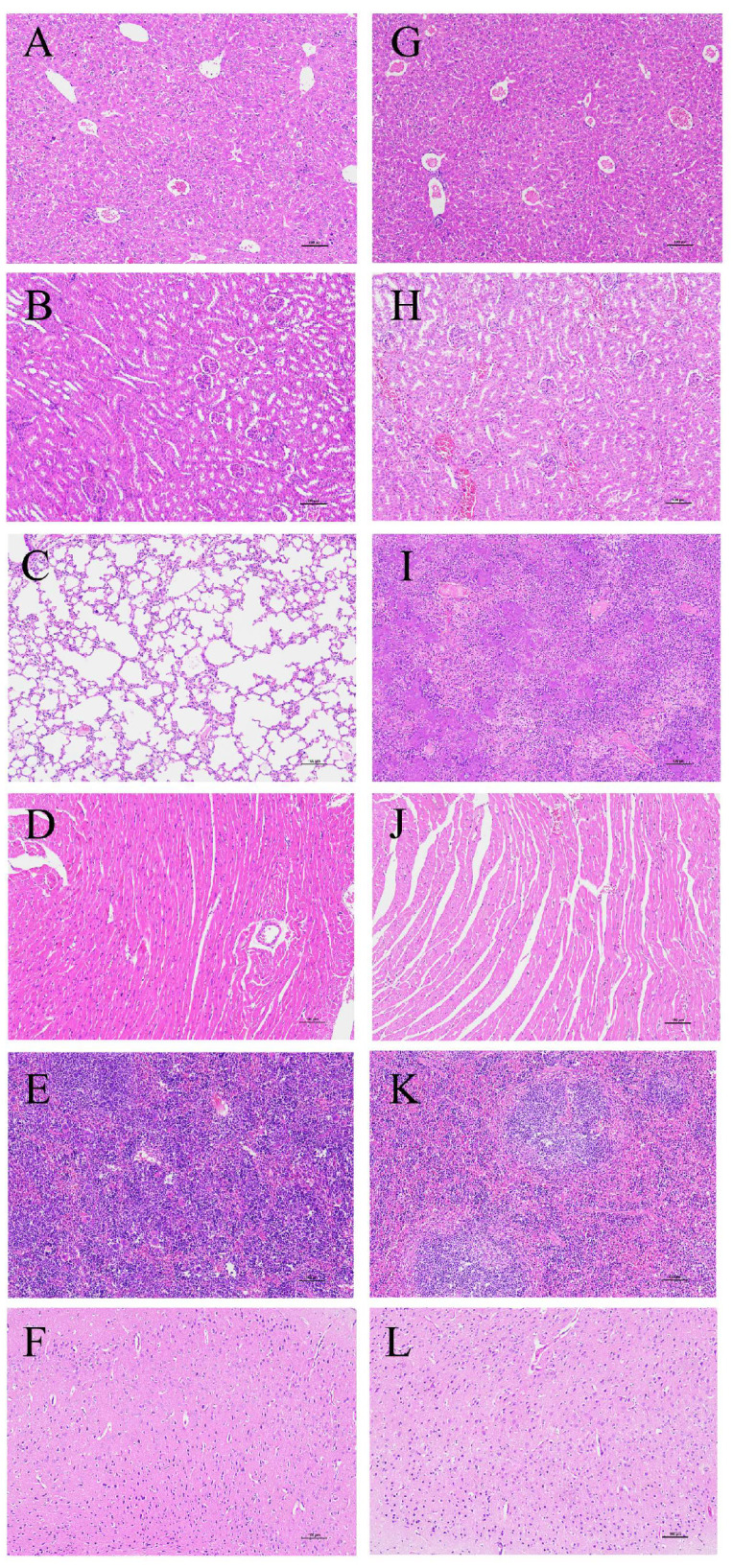
Histopathological examination of mouse tissues (1000×). (**A**–**F**): Representative images of HE-stained pathological sections of the liver, kidney, lung, heart, spleen, and brain from a randomly selected mouse in group F, which was injected intraperitoneally with 0.2 mL of physiological saline. (**G**–**L**): Representative images of HE-stained pathological sections of the liver, kidney, lung, heart, spleen, and brain from a randomly selected mouse in group B, which was injected intraperitoneally with 0.2 mL of SS2 strain ST01 at a concentration of 1.0 × 10^9^ CFU/mL.

The virulence of the SS2 strain ST01 was assessed using an in vivo mouse model. Mice were intraperitoneally injected with varying concentrations of ST01, specifically 1.0 × 10^10^, 1.0 × 10^9^, 1.0 × 10^8^, 1.0 × 10^7^, and 1.0 × 10^6^ CFU/mL, respectively, and mortality rates were monitored for 7 days post-infection. On the first day following the challenge, mice in groups A, B, and C exhibited symptoms such as huddling behavior, ruffled fur, significantly reduced spontaneous activity levels, and decreased food and water intake. Some individuals within these groups displayed fecal material adhering to the perianal region and presented with viscous ocular discharge accompanied by closed eyes. Conversely, mice in groups D and E did not exhibit any notable abnormal symptoms. Mice in group F maintained a normal demeanor and appetite without significant changes. Mouse deaths occurred in groups A, B, and C, while no deaths occurred in groups D, E, and F. As shown in Figure 6, the case fatality rates were 60% (6/10) in group A, 20% (2/10) in group B, and 10% (1/10) in group C. As illustrated in Figure 7, pathological examination revealed no gross lesions presented in the organs of group F. In contrast, the mice intraperitoneally injected with 1.0 × 10^9^ CFU/mL of MDR SS2 strain ST01 in group B demonstrated significant hemorrhage within the meninges along with pulmonary swelling and hemorrhage; necrotic foci were also observed on both liver and kidney surfaces. Histopathological analysis indicated that all examined organs from mice of group F retained normal tissue architecture (Figure 8A–F). However, histopathological findings from SS2 strain ST01-infected mice of group B revealed several abnormalities: liver—disruption of hepatic lobular architecture characterized by nuclear pyknosis and hyperchromasia in hepatocytes alongside extensive vascular congestion (Figure 8G); kidney—mild edema of renal tubular epithelial cells exhibiting cytoplasmic vacuolation coupled with pallor as well as significant interstitial hemorrhage (Figure 8H); lung—destruction of pulmonary architecture marked by severe alveolar congestion along with prominent infiltration by heterophilic granulocytes (Figure 8I); heart—thickening of myocardial fibers accompanied by widening of interstitial spaces (Figure 8J); spleen—disorganization of splenic structure, swelling of certain cells with prominent nuclei (Figure 8K); brain—irregular neuronal architecture, indistinct nucleoli, increased cellular density, gliosis, and interstitial edema indicate inflammatory changes in the brain parenchyma (Figure 8L).

## 4. Discussion

*S. suis* is a significant global zoonotic pathogen that poses severe threats to both human and animal health. This pathogen can be carried not only by infected animals but also by healthy and clinically recovered animals, which can serve as carriers. *S. suis* has resulted in considerable economic losses within the swine industry, primarily manifesting as meningitis, septicemia, polyserositis, arthritis, and endocarditis in weaned piglets [41]. Apparently healthy asymptomatic pigs in swine production systems can serve as critical reservoirs of infection. This situation not only heightens the likelihood of *S. suis* transmission among swine populations but also increases the risk of human exposure to this pathogen for individuals who have close contact with pork products [27,42]. The misuse of antibiotics further exacerbates antimicrobial resistance in *S. suis*. Furthermore, horizontal gene transfer of resistance determinants among bacteria complicates the prevention and treatment of *S. suis* infections [43,44]. Asymptomatic pigs are recognized as reservoirs for MDR *S. suis* strains. Consequently, it is imperative to conduct ongoing isolation studies, characterization efforts, and population assessments of *S. suis* across various regions, while enhancing surveillance of its resistance patterns and investigating the underlying mechanisms contributing to this resistance [45]. To expand the whole-genome database for *S. suis* and gain insights into the genomic characteristics and protein-coding gene functions associated with SS2 isolates, we performed WGS on the SS2 strain ST01. This work provides a reliable foundation for analyzing the adaptation processes, evolutionary dynamics, and antimicrobial resistance mechanisms related to *S. suis*.

Horizontal gene transfer (HGT) refers to the movement of genetic material between organisms, playing a crucial role in genome plasticity and evolution [39,46,47]. Key elements involved in HGT—including transposons, prophages, plasmids, and GIs—facilitate the dissemination of antimicrobial resistance genes and virulence factors among bacteria. This process enhances the pathogenic potential and profiles of AMR [48]. WGS analysis of the MDR SS2 strain ST01 identified 52 transposons, four prophages, and 28 GIs. This genomic plasticity is a significant driver in the evolution of *S. suis*, enabling both the acquisition and dissemination of antimicrobial resistance genes, as well as virulence factors. Similar findings were observed in the genomic analysis of strain SS2011GZ, which also possessed numerous gene islands and resistance genes alongside considerable genomic rearrangements [49]. The presence of these mobile genetic elements underscores the potential for the emergence of new, highly adapted, and resistant clones, complicating disease management strategies. The 302 mutations associated with reduced virulence and the 34 leading to a loss of pathogenicity further suggest a complex and ongoing evolutionary adaptation, possibly in response to host immune pressures or environmental stresses.

Whole-genome sequencing revealed two CRISPR arrays on the chromosome of MDR SS2 strain ST01, indicating its capacity for adaptive immunity through spacer-mediated integration of exogenous DNA [50,51]. Furthermore, annotation uncovered a total of 1969 protein-coding genes on this chromosome; all were functionally categorized using the COG database. Among these genes are 212 that encode hypothetical proteins with unknown functions. This knowledge gap significantly complicates our ability to infer their potential roles in metabolism, proliferation, development, virulence mechanisms, and antimicrobial resistance pathways. Consequently, further functional characterization studies targeting these proteins are warranted.

Antimicrobial resistance is a defining characteristic of *S. suis* isolates [52]. Consistent with this, our analysis revealed that the clinical isolate MDR SS2 strain ST01 exhibited resistance to multiple agents across six antimicrobial classes: aminoglycosides, tetracyclines, fluoroquinolones, lincosamides, polypeptides, and nitrofurans. Whole-genome sequencing identified 19 antimicrobial resistance genes located on the ST01 chromosome. These included penicillin-binding proteins (*Pbp1a*, *Pbp1b*, *Pbp2b*, and *Pbp2x*), DNA-directed RNA polymerase (*rpoB* and *rpoC*), multidrug transporter (*lmrC* and *lmrD*), a multidrug resistance efflux pump (*pmrA*), DNA gyrase (*GyrB* and *GyrA*), CDP-diacylglycerol-glycerol-3-phosphate 3-phosphatidyltransferase (*pgsA*), DNA topoisomerase IV *(grlB* and *parC*), dihydropteroate synthase (*folP*), the 30S ribosomal protein S12 (*StrA*), tetracycline resistance ribosomal protection protein (*tetM*), elongation factor Tu (*EF-Tu*), and a luxR family response regulator. Research conducted on various *S. suis* strains indicates that several multidrug efflux pump systems significantly contribute to resistance against a range of antibiotics, including fluoroquinolones, macrolides, and tetracyclines [53,54]. Despite possessing numerous antimicrobial resistance genes, strain ST01 displayed susceptibility to fluoroquinolones, macrolides, and tetracyclines. This observation suggests that the mechanisms underlying ST01’s resistance differ from those presented in other *S. suis* strains. It is possible that some of these resistance genes are minimally active or expressed at low levels; thus, they may primarily function as components of ST01’s intrinsic resistome. Furthermore, the ST01 chromosome encodes 18 antibiotic target proteins, such as D-alanyl-alanine synthetase, alanine racemase, and putative dihydrofolate reductase, among others. This finding suggests that antibiotics like β-lactams and macrolides could be effective against this MDR strain. In the face of increasing antibiotic resistance, as highlighted in a recent review which detailed mechanisms such as PBP2x mutations for β-lactams and *erm(B)* for macrolides [55], the discovery of new targets is paramount. Our findings could inform the design of new antimicrobials or support alternative approaches like immunomodulation. For example, a recent study exploring “trained immunity” demonstrated that combining an inactivated immunomodulator (*dpB*) with a *S. suis* vaccine enhanced protection in pigs, reducing bacterial loads and lung lesions [56]. Our genomic data could complement such efforts by identifying conserved, essential proteins that are critical for bacterial survival, against which new therapeutics or next-generation vaccines can be directed.

Virulence factors are microbial properties that enable colonization within specific host species and enhance the potential for disease causation. These factors include bacterial toxins, cell surface proteins that mediate adhesion, cell surface carbohydrates, and protective proteins, as well as hydrolytic enzymes that may contribute to pathogenesis. Consequently, these findings demonstrate the complexity of both pathogenicity and pathogenic mechanisms in SS2 within animal infection models. Whole-genome sequencing identified 15 virulence genes in strain ST01, which included *GroEL*, t*ufA*, *cps4B*, *neuB*, *cpsI*, *glf*, *wbtL*, *galE*, *pavA*, *fbpS*, *clpP*, *gndA*, *hasC*, *srtC4*, and *srtB*. Subsequent in vivo infection experiments confirmed the virulence of ST01 in mice. The results indicated a median lethal dose 5.62 × 10^9^ CFU/mL for mice, which did exceed the threshold for defining high virulence (≤1.0 × 10^8^ CFU/mL). Therefore, the strain did not exhibit significant high virulence. Although the SS2 strain has the highest clinical isolation rate among its counterparts, there exist notable differences in virulence across various strains. These differences are primarily reflected in the lethality rates observed in animal models, virulence gene profiles, and antibiotic susceptibility. Generally, the combination of virulence factors—including capsular polysaccharide (*CPS*), muramidase-released protein (*MRP*), extracellular factors (*EF*), and suilysin (*SLY*)—is regarded as a typical genetic marker indicative of highly virulent strains [57,58,59]. Collectively, these findings align with the experimentally observed low-virulence phenotype exhibited by the present SS2 strain ST01. A clinical serotype 1 strain, SS2011GZ, was reported to have an LD_50_ of 4.09 × 10^4^ CFU/mL in zebrafish, and strains from Shandong were uniformly classified as highly pathogenic [60]. The specific combination of virulence genes, such as those encoding hemolysins and adhesion proteins, likely facilitates the bacterium’s ability to cause systemic infections like meningitis and arthritis, as observed in both pigs and humans [55,61]. Furthermore, the annotation of the PHI database has identified 302 mutations associated with reduced virulence, 34 mutations leading to a loss of pathogenicity, and 21 lethal mutations in strain ST01. This finding indicates that specific mutants can attenuate virulence, abolish pathogenicity, or even induce lethality in the MDR SS2 strain ST01.

Despite the extensive information generated through whole-genome sequencing, experimental validation remains essential for characterizing the specific traits of ST01 isolates. Therefore, future investigations must empirically verify the properties and pathogenic mechanisms of this MDR strain ST01. Future research directions should encompass comprehensive epidemiological investigations addressing four key aspects: the prevalence of SS2 isolates among food-producing animals; their associated antimicrobial resistance profiles, virulence determinants, and pathogenic potential; and zoonotic connections with human isolates.

## 5. Conclusions

In this study, it was found that the MDR SS2 strain ST01, isolated from pigs, possesses a circular chromosome containing 19 antimicrobial resistance genes and 15 virulence genes. The ST01 strain may constitute a significant threat to global health due to its virulence and resistance to six classes of antibiotics, including several aminoglycosides, tetracyclines, fluoroquinolones, lincosamides, polypeptide, and nitrofurans. Notably, whole-genome sequencing analysis identified 18 antibiotic targets and 21 lethal mutations on the ST01 chromosome, which may serve as potential targets for the prevention, control, and treatment of infections caused by MDR SS2 strain ST01.

## Figures and Tables

**Table 1:** Results of biochemical identification of SS2 strain ST01.

Biochemical Test	Results	Biochemical Test	Results
Glucose Peptone	−	*L*-Arabinose	−
Esculin	+	Mannite	+
6.5% Salt Meat Broth	−	Melezitose	−
pH 9.6 Broth	−	*D*-ribose	−
Raffinose	+	Lnulin	+
Lactose	+	Glycerin	+
Sorbitol	−	Hippurate	−
Mannose	+		

+ positive; − negative.

**Table 2:** General features of SS2 strain ST01.

Genomic Parameter	Value for Parameter
Sequence length/bp	2,056,339
GC content/%	41.30
ORF number	1973
tRNA copy number	56
rRNA copy number	12
ncRNA copy number	34
CRISPRs number	2
Prophage number	4
Short interspersed repeats number	6
Long interspersed repeats number	35
Long terminal repeats number	111
Transposons number	52
Unclassified interspersed repeats number	5
Satellites RNA number	6
Simple-repeats number	0

**Table 3:** Summary of virulence factors on the chromosome of SS2 strain ST01 using VFDB.

ORF Name	Gene Name (Abbreviations)	Function	Gene Size/bp	Description	VFs_ID
chr_138	*groEL* (Growth essential, Large subunit)	Adherence	1623	Chaperonin GroEL	VFG012095(gb|WP_003435012)
chr_480	*tufA* (Translation elongation Factor Tu, A copy)		1197	Elongation factor Tu	VFG046465(gb|WP_003028672)
chr_1271	*fbp54* (Fructose-bisphosphate aldolase, 54 kDa)		1659	Fibronectin-bing protein Fbp54	VFG000959(gb|WP_010922232)
chr_1890	*srtC4* (Sortase Class 4)		807	Class C sortase	VFG005293(gb|WP_000508992)
chr_529	*neuB* (N-acetyleuraminate biosynthesis synthase)	Immune modulation	1017	N-acetylneuraminate synthase	VFG005894(gb|WP_000262522)
chr_553	*cpsI* (Capsular polysaccharide synthesis gene I)		1113	UDP-galactopyranose mutase	VFG002182(gb|WP_002376666)
chr_1098	*wbtL* (Wzy-biosynthesis of Transferase L)		870	Glucose-1-phosphate thymidylyltransferase	VFG047039(gb|WP_003018140)
chr_1553	*gndA* (6-phosphoGluconate Dehydrogenase, A chain)		1428	NADP-dependent phosphogluconate dehydrogenase	VFG048830(gb|WP_014907233)
chr_1839	*hasC* (Hyaluronic acid synthesis gene C)		900	UTP-glucose-1-phosphate uridylyltransferase HasC	VFG000964(gb|WP_010922799)
chr_1379	*clpP* (Caseinolytic protease Proteolytic subunit)	Stress survival	591	ATP-dependent Clp protease proteolytic subunit	VFG000077(gb|NP_465991)

Note: Only the top 10 matching genes are presented in the table.

**Table 4:** Summary of antimicrobial resistance genes on the chromosome of SS2 strain ST01 using CARD.

ORF Name	Gene Name (Abbreviations)	Gene Size/bp	Description	Identity (%)
chr_358	*tetM* (Tetracycline resistance M)	1920	Tetracycline resistance ribosomal protection protein Tet(M)	90.61
chr_703	*parC* (Paralyzed Cell division, C subunit)	2418	DNA topoisomerase IV subunit A	81.61
chr_139	*StrA* (Streptomycin Adenylyltransferase)	414	30S ribosomal protein S12	70.80
chr_957	*folP* (Dihydropteroate synthase)	813	Dihyropteroate synthase	70.63
chr_480	*tufA* (Elongation Factor Thermo Unstable, A copy)	1197	Elongation factor Tu	70.57
chr_697	*grlB* (GadRegulator locus B)	1944	DNA topoisomerase IV subunit B	70.22
chr_113	*rpoB* (RNA polymerase Beta subunit)	3573	DNA-directed RNA polymerase subunit beta	69.03
chr_114	*rpoC* (RNA polymerase Beta-prime subunit)	3621	DNA-directed RNA polymerase subunit beta	68.78
chr_1835	*imrD* (Inner membrane Resistance protein D)	1785	ABC transporter ATP-binding protein	66.67
chr_1288	*gyrB* (DNA Gyrase Subunit B)	1953	DNA topoisomerase (ATP-hydrolyzing) subunit B	65.15

Note: Only the top 10 matching genes are presented in the table.

**Table 5:** Antimicrobial susceptibility profile of SS2 strain ST01.

Antibiotics Classes		Antimicrobial Drugs	Inhibition Zone Diameter/mm	Susceptibility
β-lactams	Third-generation cephalosporins	Cefoperazone	23.07 ± 1.80	S
		Ceftriaxone	25.00 ± 1.39	S
		Ceftazidime	22.30 ± 0.70	S
	Penicillins	Ampicillin	28.17 ± 0.57	S
		Oxacillin	14.00 ± 0.00	I
		Penicillin G	25.23 ± 0.32	I
		Piperacillin	29.87 ± 0.71	S
	First-generation cephalosporins	Cephradine	25.33 ± 0.35	S
		Cefazolin	27.60 ± 0.66	S
		Cefalexin	22.20 ± 0.26	S
Aminoglycosides		Neomycin	9.67 ± 0.35	R
		Kanamycin	10.77 ± 0.59	R
		Gentamicin	9.83 ± 0.86	R
		Amikacin	9.00 ± 0.00	R
Tetracyclines		Minocycline	12.83 ± 0.29	R
		Doxycycline	14.00 ± 0.50	I
		Tetracycline	7.63 ± 0.35	R
Fluoroquinolones		Ofloxacin	19.83 ± 0.97	S
		Ciprofloxacin	20.03 ± 0.95	S
		Norfloxacin	14.00 ± 0.30	I
Macrolides		Erythromycin	25.50 ± 0.46	S
		Midecamycin	21.53 ± 0.50	S
Lincosamides		Clindamycin	19.60 ± 0.53	S
Amphenicols		Chloramphenicol	20.87 ± 0.81	S
Glycopeptides		Vancomycin	18.17 ± 0.81	S
Sulfonamides		Cotrimoxazole	22.10 ± 0.17	S
Polypeptide		Polymyxin B	0.00 ± 0.00	R
Nitrofurans		Furazolidone	0.00 ± 0.00	R

Note: S represents Susceptible; I represents Intermediate susceptible; R represents Resistant.

## Data Availability

The original contributions presented in this study are included in the article/Supplementary Material. Further inquiries can be directed to the corresponding authors.

## Supplementary Materials

The following supporting information can be downloaded at: https://www.mdpi.com/article/10.3390/microorganisms13112552/s1, Table S1: Comparisons of the chromosome of *S. Suis* 2 ST01 with S. Suis 2 published in the NCBI Genbank nucleotide sequence database; Table S2: Summary of carbohydrate-active enzymes on the *S. Suis* 2 ST01 chromosome using CAZy; Table S3: Summary of the gene mutants on the *S. Suis* 2 ST01 chromosome using PHI; Table S4: Summary of protein domain on the *S. Suis* 2 ST01 chromosome using PFAM; Table S5: Summary of proteases on the *S. Suis* 2 ST01 chromosome using MEROPS.

## Abbreviations

The following abbreviations are used in this manuscript:

*S. Suis*	*Streptococcus suis*
SS2	*Streptococcus suis* serotype 2
TSS	*Streptococcus suis* toxic shock syndrome
MDR	Multidrug-resistant
XDR	Extensively drug-resistant
PDR	Pan-drug-resistant
WGS	Whole-genome sequencing
AMR	Antimicrobial resistance
ncRNA	Non-coding RNA
rRNA	Ribosome RNA
sRNA	Small RNA
tRNA	Transfer RNA
snRNA	Small nuclear RNA
snoRNA	Small nucleolar RNA
miRNA	microRNA
NR	Non-Redundant Protein Database
GO	Gene Ontology
KEGG	Kyoto Encyclopedia of Genes and Genomes
COG	Clusters of Orthologous Groups
TCDB	Transporter Classification Database
CAZy	Carbohydrate-Active Enzymes Database
PHI	Pathogen–Host Interactions Database
