# Supplementary material for: Analysis of Antimicrobial Resistance and Virulence Factors in Multidrug-Resistant Streptococcus suis Serotype 2 Isolates Using Whole-Genome Sequencing
Source: Microorganisms. 2025 Nov 7;13(11):2552. doi: 10.3390/microorganisms13112552 (PMC12654837; doi:10.3390/microorganisms13112552)
Supplement: Supplementary file 1 [file microorganisms-13-02552-s001.zip › Table. S1 Comparisons of the chromosome of S. Suis 2 ST01 with S. Suis 2 published in the NCBI Genbank nucleotide sequence database.pdf]

An official website of the United States government

Here's how you know

The .gov means it’s official.
Federal government websites often end in .gov or .mil. Before sharing sensitive information, make sure you’re on a federal government site.

Feedback

The site is secure.
The **https://** ensures that you are connecting to the official website and that any information you provide is encrypted and transmitted securely.
Access keys NCBI Homepage MyNCBI Homepage Main Content Main Navigation

BLAST ® » blastn suite » results for RID-KKHNRNDR013

Check out the ClusteredNR database on BLAST+ Learn more Give us feedback ×

Job Title	Z3880-5 ...
RID	KKHNRNDR013 Search expires on 10-27 09:53 am
Program	BLASTN
Database	rRNA_typestrains/16S_ribosomal_RNA
Query ID	lcl|Query_27003
Description	04F+D+1500+...+a2310259699n-Z3880-5-Contig1 ...
Molecule type	dna
Query Length	1468

Descriptions

Description	Scientific Name	Max Score	Total Score	Query Cover	E value	Per. Ident	Acc. Len	Accession
Streptococcus suis strain S735 16S ribosomal RNA, partial sequence	Streptococcus suis	2684	2684	99%	0.0	99.86%	1535	NR_036918.1

Description	Scientific Name	Max Score	Total Score	Query Cover	E value	Per. Ident	Acc. Len	Accession
Streptococcus suis strain ATCC 43765 16S ribosomal RNA, partial sequence	Streptococcus suis	2667	2667	98%	0.0	99.86%	1476	NR_115737.1
Streptococcus parasuis strain SUT-286 16S ribosomal RNA, partial sequence	Streptococcus parasuis	2501	2501	99%	0.0	97.61%	1533	NR_179215.1
Streptococcus suis strain ATCC 43765 16S ribosomal RNA, partial sequence	Streptococcus suis	2462	2462	91%	0.0	99.93%	1336	NR_117504.1
Streptococcus oriscaviae strain HKU75 16S ribosomal RNA, complete sequence	Streptococcus oriscaviae	2435	2435	99%	0.0	96.55%	1557	NR_181935.1
Streptococcus oriscaviae strain HKU75 16S ribosomal RNA, partial sequence	Streptococcus oriscaviae	2435	2435	99%	0.0	96.55%	1512	NR_181481.1
Streptococcus respiraculi strain HTS25 16S ribosomal RNA, partial sequence	Streptococcus respiraculi	2427	2427	99%	0.0	96.53%	1510	NR_179720.1
Streptococcus gallinaceus strain CCUG 42692 16S ribosomal RNA, partial sequence	Streptococcus gallinaceus	2401	2401	98%	0.0	96.62%	1502	NR_025453.1
Streptococcus plurextorum strain 1956-02 16S ribosomal RNA, partial sequence	Streptococcus plurextorum	2398	2398	98%	0.0	96.50%	1451	NR_042649.1
Streptococcus saliviloxodontae strain NUM 6306 16S ribosomal RNA, partial sequence	Streptococcus saliviloxodontae	2396	2396	98%	0.0	96.54%	1504	NR_126178.1
Streptococcus parasuis strain SUT-286 16S ribosomal RNA, partial sequence	Streptococcus parasuis	2388	2388	91%	0.0	98.81%	1411	NR_178262.1
Streptococcus himalayensis strain HTS2 16S ribosomal RNA, partial sequence	Streptococcus himalayensis	2386	2386	99%	0.0	96.06%	1509	NR_156072.1
Streptococcus koreensis strain KCOM 2890 16S ribosomal RNA, partial sequence	Streptococcus koreensis	2379	2379	98%	0.0	96.34%	1471	NR_165737.1
Streptococcus cristatus AS 1.3089 16S ribosomal RNA, partial sequence	Streptococcus cristatus AS 1.3089	2366	2366	99%	0.0	95.79%	1556	NR_103943.1
Streptococcus parasanguinis ATCC 15912 16S ribosomal RNA, partial sequence	Streptococcus parasanguinis ATCC 15912	2366	2366	99%	0.0	95.90%	1513	NR_024842.1
Streptococcus marmotae strain HTS5 16S ribosomal RNA, partial sequence	Streptococcus marmotae	2361	2361	99%	0.0	95.72%	1510	NR_152678.1
Streptococcus porcorum strain 682/03 16S ribosomal RNA, partial sequence	Streptococcus porcorum	2357	2357	93%	0.0	97.54%	1380	NR_108477.1
Streptococcus cristatus ATCC 51100 16S ribosomal RNA, partial sequence	Streptococcus cristatus ATCC 51100	2353	2353	99%	0.0	95.65%	1533	NR_042771.1
Streptococcus parasanguinis ATCC 15912 16S ribosomal RNA, partial sequence	Streptococcus parasanguinis ATCC 15912	2351	2351	98%	0.0	96.01%	1470	NR_115241.1
Streptococcus sinensis strain HKU4 16S ribosomal RNA, partial sequence	Streptococcus sinensis	2348	2348	99%	0.0	95.54%	1512	NR_028833.1
Streptococcus sanguinis SK1 = NCTC 7863 16S ribosomal RNA, partial sequence	Streptococcus sanguinis SK1 = NCTC 7863	2346	2346	99%	0.0	95.69%	1460	NR_024841.1
Streptococcus australis strain AI-1 16S ribosomal RNA, partial sequence	Streptococcus australis	2346	2346	98%	0.0	95.93%	1471	NR_036936.1
Streptococcus hyointestinalis strain ATCC 49169 16S ribosomal RNA, partial sequence	Streptococcus hyointestinalis	2342	2342	97%	0.0	96.11%	1439	NR_041780.1
Streptococcus vestibularis ATCC 49124 16S ribosomal RNA, partial sequence	Streptococcus vestibularis ATCC 49124	2342	2342	99%	0.0	95.51%	1538	NR_042777.1
Streptococcus salivarius strain ATCC 7073 16S ribosomal RNA, partial sequence	Streptococcus salivarius	2342	2342	99%	0.0	95.52%	1546	NR_042776.1
Streptococcus penaeicida strain CAIM 1838 16S ribosomal RNA, partial sequence	Streptococcus penaeicida	2340	2340	99%	0.0	95.55%	1532	NR_178901.1
Streptococcus rubneri strain LMG 27207 16S ribosomal RNA, partial sequence	Streptococcus rubneri	2340	2340	98%	0.0	95.98%	1457	NR_109720.1
Streptococcus urinalis strain 2285-97 16S ribosomal RNA gene, partial sequence	Streptococcus urinalis	2340	2340	98%	0.0	95.86%	1501	NR_037101.1
Streptococcus lutetiensis strain HDP90246 16S ribosomal RNA, partial sequence	Streptococcus lutetiensis	2338	2338	98%	0.0	95.92%	1473	NR_037096.1
Streptococcus loxodontisalivarius strain NUM 6304 16S ribosomal RNA, partial sequence	Streptococcus loxodontisalivarius	2337	2337	98%	0.0	95.79%	1506	NR_126177.1
Streptococcus urinalis strain 2285-97 16S ribosomal RNA, partial sequence	Streptococcus urinalis	2337	2337	98%	0.0	95.74%	1476	NR_115738.1
Streptococcus ilei strain I-G2 16S ribosomal RNA, partial sequence	Streptococcus ilei	2335	2335	96%	0.0	96.53%	1411	NR_178515.1
Streptococcus gordonii strain SK3 16S ribosomal RNA, partial sequence	Streptococcus gordonii	2333	2333	99%	0.0	95.39%	1512	NR_028666.1
Streptococcus equinus strain NBRC 12553 16S ribosomal RNA, partial sequence	Streptococcus equinus	2333	2333	98%	0.0	95.79%	1467	NR_113594.1
Streptococcus parasanguinis ATCC 15912 16S ribosomal RNA, partial sequence	Streptococcus parasanguinis ATCC 15912	2333	2333	97%	0.0	95.98%	1464	NR_115735.1
Streptococcus vicugnae strain SL1232 16S ribosomal RNA, partial sequence	Streptococcus vicugnae	2331	2331	99%	0.0	95.60%	1529	NR_181987.1
Streptococcus thermophilus strain ATCC 19258 16S ribosomal RNA, partial sequence	Streptococcus thermophilus	2331	2331	99%	0.0	95.38%	1539	NR_042778.1
Streptococcus lutetiensis strain CIP 106849 16S ribosomal RNA, partial sequence	Streptococcus lutetiensis	2331	2331	98%	0.0	95.91%	1470	NR_115719.1
Streptococcus uberis strain JCM 5709 16S ribosomal RNA, partial sequence	Streptococcus uberis	2329	2329	98%	0.0	95.72%	1501	NR_040820.1
Streptococcus ruminantium strain GUT-187 16S	Streptococcus	2327	2327	99%	0.0	95.44%	1546	NR_158064.1

Description	Scientific Name	Max Score	Total Score	Query Cover	E value	Per. Ident	Acc. Len	Accession
ribosomal RNA, partial sequence	ruminantium							
Streptococcus cristatus ATCC 51100 16S ribosomal RNA, partial sequence	Streptococcus cristatus ATCC 51100	2327	2327	98%	0.0	95.72%	1469	NR_115274.1
Streptococcus sanguinis strain JCM 5708 16S ribosomal RNA, partial sequence	Streptococcus sanguinis	2327	2327	98%	0.0	95.72%	1470	NR_113260.1
Streptococcus hillyeri strain 28462 16S ribosomal RNA, partial sequence	Streptococcus hillyeri	2324	2324	98%	0.0	95.66%	1501	NR_180151.1
Streptococcus agalactiae ATCC 13813 strain JCM 5671 16S ribosomal RNA, partial sequence	Streptococcus agalactiae ATCC 13813	2324	2324	98%	0.0	95.65%	1501	NR_040821.1
Streptococcus agalactiae ATCC 13813 strain JCM 5671 16S ribosomal RNA, partial sequence	Streptococcus agalactiae ATCC 13813	2324	2324	98%	0.0	95.65%	1471	NR_113262.1
Streptococcus oralis ATCC 35037 16S ribosomal RNA, partial sequence	Streptococcus oralis ATCC 35037	2318	2318	98%	0.0	95.47%	1460	NR_114413.1
Streptococcus macedonicus strain LAB617 16S ribosomal RNA, partial sequence	Streptococcus macedonicus	2318	2318	99%	0.0	95.17%	1542	NR_037002.1
Streptococcus oralis ATCC 35037 16S ribosomal RNA, partial sequence	Streptococcus oralis ATCC 35037	2318	2318	98%	0.0	95.59%	1471	NR_042927.1
Streptococcus cristatus strain 2-4 16S ribosomal RNA, partial sequence	Streptococcus cristatus	2318	2318	97%	0.0	95.83%	1510	NR_029052.1
Streptococcus oralis subsp. tigurinus AZ_3a 16S ribosomal RNA, partial sequence	Streptococcus oralis subsp. tigurinus AZ_3a	2316	2316	99%	0.0	95.23%	1476	NR_118234.1
Streptococcus oralis subsp. dentisani strain 7747 16S ribosomal RNA, partial sequence	Streptococcus oralis subsp. dentisani	2314	2314	99%	0.0	95.18%	1535	NR_117719.1
Streptococcus moroccensis strain CCMM B831 16S ribosomal RNA, partial sequence	Streptococcus moroccensis	2314	2314	98%	0.0	95.36%	1520	NR_134191.1
Streptococcus gordonii strain ATCC 10558 16S ribosomal RNA, partial sequence	Streptococcus gordonii	2314	2314	98%	0.0	95.48%	1479	NR_115242.1
Streptococcus acidominimus strain LMG 17755 16S ribosomal RNA, partial sequence	Streptococcus acidominimus	2311	2311	98%	0.0	95.52%	1500	NR_104972.1
Streptococcus oralis strain CCUG 24891 16S ribosomal RNA, partial sequence	Streptococcus oralis	2311	2311	98%	0.0	95.45%	1472	NR_115734.1
Streptococcus azizii strain 12-5202 16S ribosomal RNA, partial sequence	Streptococcus azizii	2309	2309	99%	0.0	95.10%	1538	NR_159228.1
Streptococcus agalactiae ATCC 13813 16S ribosomal RNA, partial sequence	Streptococcus agalactiae ATCC 13813	2309	2309	98%	0.0	95.62%	1465	NR_115728.1
Streptococcus tangierensis strain CCMM B832 16S ribosomal RNA, partial sequence	Streptococcus tangierensis	2307	2307	98%	0.0	95.28%	1520	NR_134818.1
Streptococcus porcinus strain 176 16S ribosomal RNA, partial sequence	Streptococcus porcinus	2307	2307	99%	0.0	95.26%	1496	NR_024634.1
Streptococcus parauberis strain DSM 6631 16S ribosomal RNA, partial sequence	Streptococcus parauberis	2307	2307	98%	0.0	95.45%	1471	NR_043001.1
Streptococcus lactarius strain MV1 16S ribosomal RNA, partial sequence	Streptococcus lactarius	2307	2307	98%	0.0	95.39%	1452	NR_117425.1
Streptococcus mitis strain NS51 16S ribosomal RNA, partial sequence	Streptococcus mitis	2302	2302	99%	0.0	95.03%	1520	NR_028664.1
Streptococcus downii 16S ribosomal RNA, partial sequence	Streptococcus downii	2300	2300	98%	0.0	95.26%	1513	NR_175455.1
Streptococcus iniae strain ATCC 29178 16S ribosomal RNA, partial sequence	Streptococcus iniae	2300	2300	99%	0.0	94.97%	1536	NR_025148.1
Streptococcus vulneris strain DM3B3 16S ribosomal RNA, complete sequence	Streptococcus vulneris	2298	2298	99%	0.0	94.98%	1547	NR_179383.1
Streptococcus alactolyticus strain ATCC 43077 16S ribosomal RNA, partial sequence	Streptococcus alactolyticus	2298	2298	97%	0.0	95.55%	1437	NR_041781.1
Streptococcus equinus strain NCDO 1037 16S ribosomal RNA, partial sequence	Streptococcus equinus	2296	2296	97%	0.0	95.67%	1463	NR_114642.1
Streptococcus toyakuensis strain TP1632 16S ribosomal RNA, partial sequence	Streptococcus toyakuensis	2290	2290	99%	0.0	94.90%	1543	NR_179385.1
Streptococcus ictaluri 707-05 16S ribosomal RNA, partial sequence	Streptococcus ictaluri 707-05	2290	2290	98%	0.0	95.24%	1471	NR_115802.1
Streptococcus thermophilus strain DSM 20617 16S ribosomal RNA, partial sequence	Streptococcus thermophilus	2289	2289	99%	0.0	94.84%	1540	NR_118998.1
Streptococcus panodentis strain TKU50 16S ribosomal RNA, partial sequence	Streptococcus panodentis	2289	2289	98%	0.0	95.24%	1471	NR_145950.1
Streptococcus cameli strain CCMM B834 16S ribosomal RNA, partial sequence	Streptococcus cameli	2289	2289	98%	0.0	95.08%	1518	NR_134817.1
Streptococcus infantis ATCC 700779 16S ribosomal RNA, partial sequence	Streptococcus infantis ATCC 700779	2287	2287	98%	0.0	95.24%	1468	NR_042928.1
Streptococcus pasteurianus strain CIP 107122 16S ribosomal RNA, partial sequence	Streptococcus pasteurianus	2287	2287	98%	0.0	95.36%	1470	NR_043660.1
Streptococcus timonensis strain Marseille-P2915 16S ribosomal RNA, partial sequence	Streptococcus timonensis	2285	2285	99%	0.0	94.83%	1509	NR_179540.1
Streptococcus cuniculipharyngis strain DICM10-00796B 16S ribosomal RNA, partial sequence	Streptococcus cuniculipharyngis	2285	2285	96%	0.0	95.77%	1417	NR_137219.1
Streptococcus troglodytidis strain M09-11185 16S ribosomal RNA, partial sequence	Streptococcus troglodytidis	2283	2283	99%	0.0	94.73%	1515	NR_109371.1
Streptococcus cuniculi strain : NED12-00049-6B 16S ribosomal RNA, partial sequence	Streptococcus cuniculi	2281	2281	96%	0.0	95.65%	1423	NR_134190.1
Streptococcus minor strain ON59 16S ribosomal RNA, partial sequence	Streptococcus minor	2281	2281	99%	0.0	94.89%	1497	NR_025729.1

Description	Scientific Name	Max Score	Total Score	Query Cover	E value	Per. Ident	Acc. Len	Accession
Streptococcus varani strain FF10 16S ribosomal RNA, partial sequence	Streptococcus varani	2279	2279	99%	0.0	94.66%	1522	NR_179404.1
Streptococcus dysgalactiae subsp. equisimilis strain CIP 105120 16S ribosomal RNA, partial sequence	Streptococcus dysgalactiae subsp. equisimilis	2279	2279	100%	0.0	94.70%	1487	NR_043661.1
Streptococcus xiaochunlingii strain E24 16S ribosomal RNA, partial sequence	Streptococcus xiaochunlingii	2278	2278	96%	0.0	95.44%	1437	NR_180875.1
Streptococcus mitis strain ATCC 49456 16S ribosomal RNA, partial sequence	Streptococcus mitis	2278	2278	98%	0.0	95.10%	1469	NR_115240.1
Streptococcus porci strain 2923-03 16S ribosomal RNA, partial sequence	Streptococcus porci	2278	2278	93%	0.0	96.58%	1388	NR_115087.1
Streptococcus constellatus strain ATCC 27823 16S ribosomal RNA, partial sequence	Streptococcus constellatus	2276	2276	99%	0.0	94.72%	1558	NR_041721.1
Streptococcus pseudoporcinus LQ 940-04 16S ribosomal RNA, partial sequence	Streptococcus pseudoporcinus LQ 940-04	2276	2276	97%	0.0	95.39%	1456	NR_043704.1
Streptococcus pyogenes strain JCM 5674 16S ribosomal RNA, partial sequence	Streptococcus pyogenes	2274	2274	98%	0.0	95.03%	1501	NR_112088.1
Streptococcus lutetiensis strain NEM 782 16S ribosomal RNA, partial sequence	Streptococcus lutetiensis	2274	2274	94%	0.0	96.38%	1461	NR_042051.1
Streptococcus intermedius strain 1877 16S ribosomal RNA, partial sequence	Streptococcus intermedius	2270	2270	99%	0.0	94.65%	1544	NR_028736.1
Streptococcus mitis strain ATCC 49456 16S ribosomal RNA, partial sequence	Streptococcus mitis	2270	2270	98%	0.0	94.97%	1446	NR_115732.1
Streptococcus gwangjuense strain ChDC B345 16S ribosomal RNA, partial sequence	Streptococcus gwangjuense	2268	2268	99%	0.0	94.86%	1477	NR_165744.1
Streptococcus oricebi strain M8 16S ribosomal RNA, partial sequence	Streptococcus oricebi	2263	2263	98%	0.0	94.90%	1504	NR_148589.1
Streptococcus chosunense strain ChDC B353 16S ribosomal RNA, partial sequence	Streptococcus chosunense	2254	2254	99%	0.0	94.65%	1477	NR_165741.1
Streptococcus anginosus SK52 = DSM 20563 16S ribosomal RNA, partial sequence	Streptococcus anginosus SK52 = DSM 20563	2254	2254	98%	0.0	94.90%	1477	NR_117426.1
Streptococcus constellatus subsp. pharyngis strain MM9889a 16S ribosomal RNA, partial sequence	Streptococcus constellatus subsp. pharyngis	2252	2252	98%	0.0	94.84%	1470	NR_042833.1
Streptococcus sanguinis SK1 = NCTC 7863 16S ribosomal RNA, partial sequence	Streptococcus sanguinis SK1 = NCTC 7863	2248	2248	94%	0.0	96.02%	1420	NR_115736.1
Streptococcus oriloxodontae strain NUM 2101 16S ribosomal RNA, partial sequence	Streptococcus oriloxodontae	2246	2246	99%	0.0	94.25%	1550	NR_178254.1
Streptococcus pneumoniae strain ATCC 33400 16S ribosomal RNA, partial sequence	Streptococcus pneumoniae	2246	2246	99%	0.0	94.43%	1515	NR_028665.1
Streptococcus iniae strain ATCC 29178 16S ribosomal RNA, partial sequence	Streptococcus iniae	2244	2244	98%	0.0	94.74%	1466	NR_115731.1
Streptococcus caledonicus strain S784/96/1 16S ribosomal RNA, partial sequence	Streptococcus caledonicus	2242	2242	98%	0.0	94.56%	1485	NR_180776.1

Graphic Summary

Distribution of the top 100 Blast Hits on 100 subject sequences

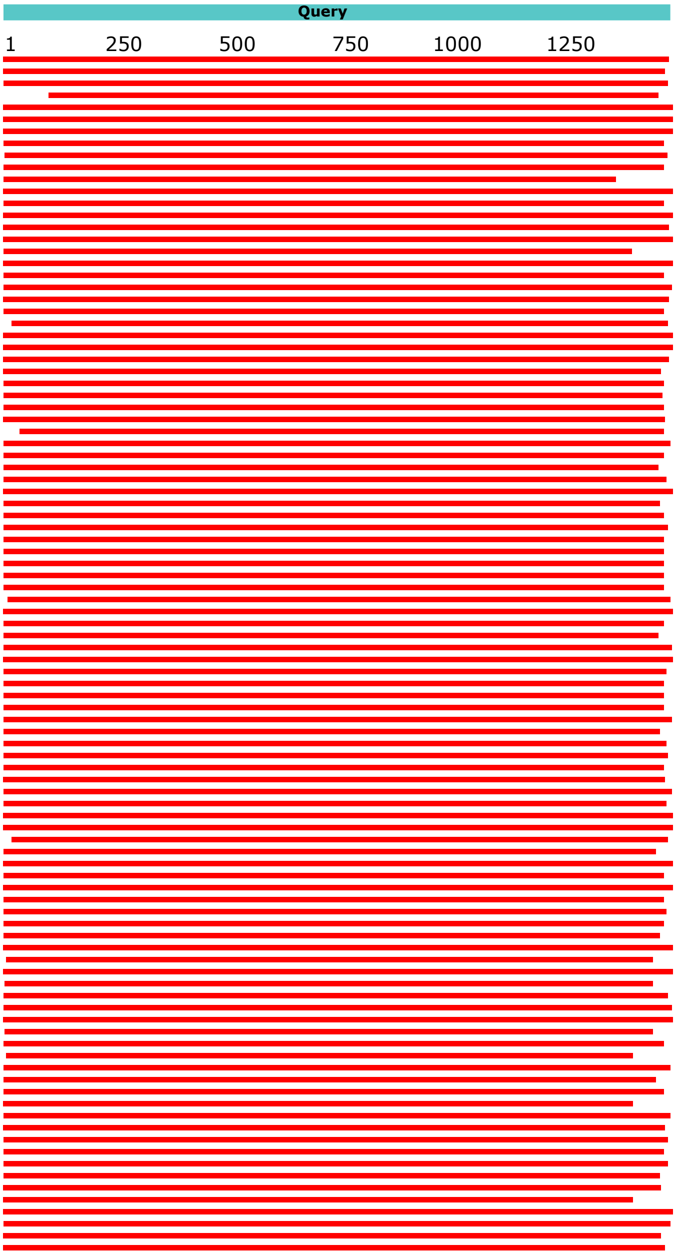

Alignments

Alignment view Pairwise ☐ CDS feature Restore defaults

Streptococcus suis strain S735 16S ribosomal RNA, partial sequence
Sequence ID: **NR_036918.1** Length: 1535 Number of Matches: 1
Range 1: 5 to 1464

Score	Expect	Identities	Gaps	Strand	Frame
2684 bits(1453)	0.0()	1458/1460(99%)	1/1460(0%)	Plus/Minus	

```
Query  1     AGGCGGCTGGCTCCCAAAAGGTTACCTCACCGACTTCGGGTGTTACAAACTCTCGTGGTG  60
             ||||||||||||| ||||||||||||||||||||||||||||||||||||||||||||||
Sbjct  1464  AGGCGGCTGGCTCCTAAAAGGTTACCTCACCGACTTCGGGTGTTACAAACTCTCGTGGTG  1405

Query  61    TGACGGGCGGTGTGTACAAGGCCCGGGAACGTATTCACCGCGGCGTGCTGATCCGCGATT  120
             ||||||||||||||||||||||||||||||||||||||||||||||||||||||||||||
Sbjct  1404  TGACGGGCGGTGTGTACAAGGCCCGGGAACGTATTCACCGCGGCGTGCTGATCCGCGATT  1345

Query  121   ACTAGCGATTCCGACTTCATGTAGGCGAGTTGCAGCCTACAATCCGAACTGAGACTGGCT  180
             ||||||||||||||||||||||||||||||||||||||||||||||||||||||||||||
Sbjct  1344  ACTAGCGATTCCGACTTCATGTAGGCGAGTTGCAGCCTACAATCCGAACTGAGACTGGCT  1285

Query  181   TTAAGAGATTAGCTTGCCGTCACCGACTTGCGACTCGTTGTACCAGCCATTGTAGCACGT  240
             ||||||||||||||||||||||||||||||||||||||||||||||||||||||||||||
Sbjct  1284  TTAAGAGATTAGCTTGCCGTCACCGACTTGCGACTCGTTGTACCAGCCATTGTAGCACGT  1225

Query  241   GTGTAGCCCAGGTCATAAGGGGCATGATGATTTGACGTCATCCCCACCTTCCTCCGGTTT  300
             ||||||||||||||||||||||||||||||||||||||||||||||||||||||||||||
Sbjct  1224  GTGTAGCCCAGGTCATAAGGGGCATGATGATTTGACGTCATCCCCACCTTCCTCCGGTTT  1165

Query  301   ATTACCGGCAGTCTCGCTAGAGTGCCCAACTGAATGATGGCAACTAACAATAGGGGTTGC  360
             ||||||||||||||||||||||||||||||||||||||||||||||||||||||||||||
Sbjct  1164  ATTACCGGCAGTCTCGCTAGAGTGCCCAACTGAATGATGGCAACTAACAATAGGGGTTGC  1105

Query  361   GCTCGTTGCGGGACTTAACCCAACATCTCACGACACGAGCTGACGACAACCATGCACCAC  420
             ||||||||||||||||||||||||||||||||||||||||||||||||||||||||||||
Sbjct  1104  GCTCGTTGCGGGACTTAACCCAACATCTCACGACACGAGCTGACGACAACCATGCACCAC  1045

Query  421   CTGTCACCGATGCTCCGAAGAGAAACCCTATCTCTAGGGCGGTCATCGGGATGTCAAGAC  480
             ||||||||||||||||||||||||||||||||||||||||||||||||||||||||||||
Sbjct  1044  CTGTCACCGATGCTCCGAAGAGAAACCCTATCTCTAGGGCGGTCATCGGGATGTCAAGAC  985
```

```
Query  481   CTGGTAAGGTTCTTCGCGTTGCTTCGAATTAAACCACATGCTCCACCGCTTGTGCGGGCC  540
             ||||||||||||||||||||||||||||||||||||||||||||||||||||||||||||
Sbjct  984   CTGGTAAGGTTCTTCGCGTTGCTTCGAATTAAACCACATGCTCCACCGCTTGTGCGGGCC  925

Query  541   CCCGTCAATTCCTTTGAGTTTCAACCTTGCGGTCGTACTCCCCAGGCGGAGTGCTTAATG  600
             ||||||||||||||||||||||||||||||||||||||||||||||||||||||||||||
Sbjct  924   CCCGTCAATTCCTTTGAGTTTCAACCTTGCGGTCGTACTCCCCAGGCGGAGTGCTTAATG  865

Query  601   CGTTAGCTGCGGCACTGAGTCCCGGAAAGGACCCAACACCTAGCACTCATCGTTTACGGC  660
             ||||||||||||||||||||||||||||||||||||||||||||||||||||||||||||
Sbjct  864   CGTTAGCTGCGGCACTGAGTCCCGGAAAGGACCCAACACCTAGCACTCATCGTTTACGGC  805

Query  661   GTGGACTACCAGGGTATCTAATCCTGTTCGCTCCCCACGCTTTCGAGCCTCAGCGTCAGT  720
             ||||||||||||||||||||||||||||||||||||||||||||||||||||||||||||
Sbjct  804   GTGGACTACCAGGGTATCTAATCCTGTTCGCTCCCCACGCTTTCGAGCCTCAGCGTCAGT  745

Query  721   TACAGACCAGAGAGCCGCTTTCGCCACCGGTGTTCCTCCATATATCTACGCATTTCACCG  780
             ||||||||||||||||||||||||||||||||||||||||||||||||||||||||||||
Sbjct  744   TACAGACCAGAGAGCCGCTTTCGCCACCGGTGTTCCTCCATATATCTACGCATTTCACCG  685

Query  781   CTACACATGGAATTCCACTCTCCCCTTCTGCACTCAAGTTTGACAGTTTCCAAAGCGTAC  840
             ||||||||||||||||||||||||||||||||||||||||||||||||||||||||||||
Sbjct  684   CTACACATGGAATTCCACTCTCCCCTTCTGCACTCAAGTTTGACAGTTTCCAAAGCGTAC  625

Query  841   TATGGTTAAGCCACAGCCTTTTACTTCAGACTTATCAAACCGCCTGCGCTCGCTTTACGC  900
             ||||||||||||||||||||||||||||||||||||||||||||||||||||||||||||
Sbjct  624   TATGGTTAAGCCACAGCCTTTTACTTCAGACTTATCAAACCGCCTGCGCTCGCTTTACGC  565

Query  901   CCAATAAATCCGGACAACGCTCGGGACCTACGTATTACCGCGGCTGCTGGCACGTAGTTA  960
             ||||||||||||||||||||||||||||||||||||||||||||||||||||||||||||
Sbjct  564   CCAATAAATCCGGACAACGCTCGGGACCTACGTATTACCGCGGCTGCTGGCACGTAGTTA  505

Query  961   GCCGTCCCTTTCTGGTAAGATACCGTCAAGTGAGAAACTTTCCACTCTTCTCACAGTTCT  1020
             ||||||||||||||||||||||||||||||||||||||||||||||||||||||||||||
Sbjct  504   GCCGTCCCTTTCTGGTAAGATACCGTCAAGTGAGAAACTTTCCACTCTTCTCACAGTTCT  445

Query  1021  TCTCTTACAACAGAGCTTTACGATCCGAAAACCTTCTTCACTCACGCGGCGTTGCTCGGT  1080
             ||||||||||||||||||||||||||||||||||||||||||||||||||||||||||||
Sbjct  444   TCTCTTACAACAGAGCTTTACGATCCGAAAACCTTCTTCACTCACGCGGCGTTGCTCGGT  385

Query  1081  CAGGGTTGCCCCCATTGCCGAAGATTCCCTACTGCTGCCTCCCGTAGGAGTCTGGGCCGT  1140
             ||||||||||||||||||||||||||||||||||||||||||||||||||||||||||||
Sbjct  384   CAGGGTTGCCCCCATTGCCGAAGATTCCCTACTGCTGCCTCCCGTAGGAGTCTGGGCCGT  325

Query  1141  GTCTCAGTCCCAGTGTGGCCGATCACCCTCTCAGGTCGGCTATGTATCGAAGCCTTGGTG  1200
             ||||||||||||||||||||||||||||||||||||||||||||||||||||||||||||
Sbjct  324   GTCTCAGTCCCAGTGTGGCCGATCACCCTCTCAGGTCGGCTATGTATCGAAGCCTTGGTG  265

Query  1201  AGCCGTTACCTCACCAACTAGCTAATACAACGCAGGTCCATCTCATAGTGAAGCAATTGC  1260
             ||||||||||||||||||||||||||||||||||||||||||||||||||||||||||||
Sbjct  264   AGCCGTTACCTCACCAACTAGCTAATACAACGCAGGTCCATCTCATAGTGAAGCAATTGC  205

Query  1261  TCCTTTCAAATATCTACCATGCGGTAAATACTGTTATGCGGTATTAGCTATCGTTTCCAA  1320
             ||||||||||||||||||||||||||||||||||||||||||||||||||||||||||||
Sbjct  204   TCCTTTCAAATATCTACCATGCGGTAAATACTGTTATGCGGTATTAGCTATCGTTTCCAA  145

Query  1321  TAGTTATCCCCCGCTATGAGGCAGGTTACCTACGCGTTACTCACCCGTTCGCAACTCATC  1380
             ||||||||||||||||||||||||||||||||||||||||||||||||||||||||||||
Sbjct  144   TAGTTATCCCCCGCTATGAGGCAGGTTACCTACGCGTTACTCACCCGTTCGCAACTCATC  85

Query  1381  CGTCTAGTGCAAGCACCAGACTTCAGCGTTCTACTTGCATGTATTAGGCACGCCGCCAGC  1440
             ||||||||||||||||||||||||||||||||||||||||||||||||||||||||||||
Sbjct  84    CGTCTAGTGCAAGCACCAGACTTCAGCGTTCTACTTGCATGTATTAGGCACGCCGCCAGC  25

Query  1441  GTTCGTCCTGAGCCAG-ATC  1459
             |||||||||||||||| |||
Sbjct  24    GTTCGTCCTGAGCCAGGATC  5
```

Streptococcus suis strain ATCC 43765 16S ribosomal RNA, partial sequence

Sequence ID: **NR_115737.1** Length: 1476 Number of Matches: 1

Range 1: 1 to 1450

Score	Expect	Identities	Gaps	Strand	Frame
2667 bits(1444)	0.0()	1449/1451(99%)	1/1451(0%)	Plus/Minus	

```
Query  1     AGGCGGCTGGCTCCCAAAAGGTTACCTCACCGACTTCGGGTGTTACAAACTCTCGTGGTG  60
             |||||||||||||| |||||||||||||||||||||||||||||||||||||||||||||
Sbjct  1450  AGGCGGCTGGCTCCTAAAAGGTTACCTCACCGACTTCGGGTGTTACAAACTCTCGTGGTG  1391

Query  61    TGACGGGCGGTGTGTACAAGGCCCGGGAACGTATTCACCGCGGCGTGCTGATCCGCGATT  120
             ||||||||||||||||||||||||||||||||||||||||||||||||||||||||||||
Sbjct  1390  TGACGGGCGGTGTGTACAAGGCCCGGGAACGTATTCACCGCGGCGTGCTGATCCGCGATT  1331

Query  121   ACTAGCGATTCCGACTTCATGTAGGCGAGTTGCAGCCTACAATCCGAACTGAGACTGGCT  180
             ||||||||||||||||||||||||||||||||||||||||||||||||||||||||||||
Sbjct  1330  ACTAGCGATTCCGACTTCATGTAGGCGAGTTGCAGCCTACAATCCGAACTGAGACTGGCT  1271

Query  181   TTAAGAGATTAGCTTGCCGTCACCGACTTGCGACTCGTTGTACCAGCCATTGTAGCACGT  240
             ||||||||||||||||||||||||||||||||||||||||||||||||||||||||||||
Sbjct  1270  TTAAGAGATTAGCTTGCCGTCACCGACTTGCGACTCGTTGTACCAGCCATTGTAGCACGT  1211

Query  241   GTGTAGCCCAGGTCATAAGGGGCATGATGATTTGACGTCATCCCCACCTTCCTCCGGTTT  300
             ||||||||||||||||||||||||||||||||||||||||||||||||||||||||||||
Sbjct  1210  GTGTAGCCCAGGTCATAAGGGGCATGATGATTTGACGTCATCCCCACCTTCCTCCGGTTT  1151

Query  301   ATTACCGGCAGTCTCGCTAGAGTGCCCAACTGAATGATGGCAACTAACAATAGGGGTTGC  360
             ||||||||||||||||||||||||||||||||||||||||||||||||||||||||||||
Sbjct  1150  ATTACCGGCAGTCTCGCTAGAGTGCCCAACTGAATGATGGCAACTAACAATAGGGGTTGC  1091

Query  361   GCTCGTTGCGGGACTTAACCCAACATCTCACGACACGAGCTGACGACAACCATGCACCAC  420
             ||||||||||||||||||||||||||||||||||||||||||||||||||||||||||||
Sbjct  1090  GCTCGTTGCGGGACTTAACCCAACATCTCACGACACGAGCTGACGACAACCATGCACCAC  1031

Query  421   CTGTCACCGATGCTCCGAAGAGAAACCCTATCTCTAGGGCGGTCATCGGGATGTCAAGAC  480
             ||||||||||||||||||||||||||||||||||||||||||||||||||||||||||||
Sbjct  1030  CTGTCACCGATGCTCCGAAGAGAAACCCTATCTCTAGGGCGGTCATCGGGATGTCAAGAC  971

Query  481   CTGGTAAGGTTCTTCGCGTTGCTTCGAATTAAACCACATGCTCCACCGCTTGTGCGGGCC  540
             ||||||||||||||||||||||||||||||||||||||||||||||||||||||||||||
Sbjct  970   CTGGTAAGGTTCTTCGCGTTGCTTCGAATTAAACCACATGCTCCACCGCTTGTGCGGGCC  911

Query  541   CCCGTCAATTCCTTTGAGTTTCAACCTTGCGGTCGTACTCCCCAGGCGGAGTGCTTAATG  600
             ||||||||||||||||||||||||||||||||||||||||||||||||||||||||||||
Sbjct  910   CCCGTCAATTCCTTTGAGTTTCAACCTTGCGGTCGTACTCCCCAGGCGGAGTGCTTAATG  851

Query  601   CGTTAGCTGCGGCACTGAGTCCCGGAAAGGACCCAACACCTAGCACTCATCGTTTACGGC  660
             ||||||||||||||||||||||||||||||||||||||||||||||||||||||||||||
Sbjct  850   CGTTAGCTGCGGCACTGAGTCCCGGAAAGGACCCAACACCTAGCACTCATCGTTTACGGC  791

Query  661   GTGGACTACCAGGGTATCTAATCCTGTTCGCTCCCCACGCTTTCGAGCCTCAGCGTCAGT  720
             ||||||||||||||||||||||||||||||||||||||||||||||||||||||||||||
Sbjct  790   GTGGACTACCAGGGTATCTAATCCTGTTCGCTCCCCACGCTTTCGAGCCTCAGCGTCAGT  731

Query  721   TACAGACCAGAGAGCCGCTTTCGCCACCGGTGTTCCTCCATATATCTACGCATTTCACCG  780
```

```
                 ||||||||||||||||||||||||||||||||||||||||||||||||||||||||||||
Sbjct  730       TACAGACCAGAGAGCCGCTTTCGCCACCGGTGTTCCTCCATATATCTACGCATTTCACCG  671

Query  781       CTACACATGGAATTCCACTCTCCCCTTCTGCACTCAAGTTTGACAGTTTCCAAAGCGTAC  840
                 ||||||||||||||||||||||||||||||||||||||||||||||||||||||||||||
Sbjct  670       CTACACATGGAATTCCACTCTCCCCTTCTGCACTCAAGTTTGACAGTTTCCAAAGCGTAC  611

Query  841       TATGGTTAAGCCACAGCCTTTTACTTCAGACTTATCAAACCGCCTGCGCTCGCTTTACGC  900
                 ||||||||||||||||||||||||||||||||||||||||||||||||||||||||||||
Sbjct  610       TATGGTTAAGCCACAGCCTTTTACTTCAGACTTATCAAACCGCCTGCGCTCGCTTTACGC  551

Query  901       CCAATAAATCCGGACAACGCTCGGGACCTACGTATTACCGCGGCTGCTGGCACGTAGTTA  960
                 ||||||||||||||||||||||||||||||||||||||||||||||||||||||||||||
Sbjct  550       CCAATAAATCCGGACAACGCTCGGGACCTACGTATTACCGCGGCTGCTGGCACGTAGTTA  491

Query  961       GCCGTCCCTTTCTGGTAAGATACCGTCAAGTGAGAAACTTTCCACTCTTCTCACAGTTCT  1020
                 ||||||||||||||||||||||||||||||||||||||||||||||||||||||||||||
Sbjct  490       GCCGTCCCTTTCTGGTAAGATACCGTCAAGTGAGAAACTTTCCACTCTTCTCACAGTTCT  431

Query  1021      TCTCTTACAACAGAGCTTTACGATCCGAAAACCTTCTTCACTCACGCGGCGTTGCTCGGT  1080
                 ||||||||||||||||||||||||||||||||||||||||||||||||||||||||||||
Sbjct  430       TCTCTTACAACAGAGCTTTACGATCCGAAAACCTTCTTCACTCACGCGGCGTTGCTCGGT  371

Query  1081      CAGGGTTGCCCCCATTGCCGAAGATTCCCTACTGCTGCCTCCCGTAGGAGTCTGGGCCGT  1140
                 ||||||||||||||||||||||||||||||||||||||||||||||||||||||||||||
Sbjct  370       CAGGGTTGCCCCCATTGCCGAAGATTCCCTACTGCTGCCTCCCGTAGGAGTCTGGGCCGT  311

Query  1141      GTCTCAGTCCCAGTGTGGCCGATCACCCTCTCAGGTCGGCTATGTATCGAAGCCTTGGTG  1200
                 ||||||||||||||||||||||||||||||||||||||||||||||||||||||||||||
Sbjct  310       GTCTCAGTCCCAGTGTGGCCGATCACCCTCTCAGGTCGGCTATGTATCGAAGCCTTGGTG  251

Query  1201      AGCCGTTACCTCACCAACTAGCTAATACAACGCAGGTCCATCTCATAGTGAAGCAATTGC  1260
                 ||||||||||||||||||||||||||||||||||||||||||||||||||||||||||||
Sbjct  250       AGCCGTTACCTCACCAACTAGCTAATACAACGCAGGTCCATCTCATAGTGAAGCAATTGC  191

Query  1261      TCCTTTCAAATATCTACCATGCGGTAAATACTGTTATGCGGTATTAGCTATCGTTTCCAA  1320
                 ||||||||||||||||||||||||||||||||||||||||||||||||||||||||||||
Sbjct  190       TCCTTTCAAATATCTACCATGCGGTAAATACTGTTATGCGGTATTAGCTATCGTTTCCAA  131

Query  1321      TAGTTATCCCCCGCTATGAGGCAGGTTACCTACGCGTTACTCACCCGTTCGCAACTCATC  1380
                 ||||||||||||||||||||||||||||||||||||||||||||||||||||||||||||
Sbjct  130       TAGTTATCCCCCGCTATGAGGCAGGTTACCTACGCGTTACTCACCCGTTCGCAACTCATC  71

Query  1381      CGTCTAGTGCAAGCACCAGACTTCAGCGTTCTACTTGCATGTATTAGGCACGCCGCCAGC  1440
                 ||||||||||||||||||||||||||||||||||||||||||||||||||||||||||||
Sbjct  70        CGTCTAGTGCAAGCACCAGACTTCAGCGTTCTACTTGCATGTATTAGGCACGCCGCCAGC  11

Query  1441      GTTCGTCCTGA  1451
                 ||||||| |||
Sbjct  10        GTTCGTC-TGA  1
```

Streptococcus parasuis strain SUT-286 16S ribosomal RNA, partial sequence
Sequence ID: **NR_179215.1** Length: 1533 Number of Matches: 1
Range 1: 1 to 1463

Score	Expect	Identities	Gaps	Strand	Frame
2501 bits(1354)	0.0()	1430/1465(98%)	12/1465(0%)	Plus/Minus	

```
Query  1         AGGCGGCTGGCTCCCAAAAGGTTACCTCACCGACTTCGGGTGTTACAAACTCTCGTGGTG  60
                 |||||||||||||| ||| |||||||||||||||||||||||||||||||||||||||||
Sbjct  1463      AGGCGGCTGGCTCCTAAATGGTTACCTCACCGACTTCGGGTGTTACAAACTCTCGTGGTG  1404

Query  61        TGACGGGCGGTGTGTACAAGGCCCGGGAACGTATTCACCGCGGCGTGCTGATCCGCGATT  120
                 ||||||||||||||||||||||||||||||||||||||||||||||||||||||||||||
Sbjct  1403      TGACGGGCGGTGTGTACAAGGCCCGGGAACGTATTCACCGCGGCGTGCTGATCCGCGATT  1344

Query  121       ACTAGCGATTCCGACTTCATGTAGGCGAGTTGCAGCCTACAATCCGAACTGAGACTGGCT  180
                 ||||||||||||||||||||||||||||||||||||||||||||||||||||||||||||
Sbjct  1343      ACTAGCGATTCCGACTTCATGTAGGCGAGTTGCAGCCTACAATCCGAACTGAGACTGGCT  1284

Query  181       TTAAGAGATTAGCTTGCCGTCACCGACTTGCGACTCGTTGTACCAGCCATTGTAGCACGT  240
                 ||||||||||||||||||||||||||||||||||||||||||||||||||||||||||||
Sbjct  1283      TTAAGAGATTAGCTTGCCGTCACCGACTTGCGACTCGTTGTACCAGCCATTGTAGCACGT  1224

Query  241       GTGTAGCCCAGGTCATAAGGGGCATGATGATTTGACGTCATCCCCACCTTCCTCCGGTTT  300
                 ||||||||||||||||||||||||||||||||||||||||||||||||||||||||||||
Sbjct  1223      GTGTAGCCCAGGTCATAAGGGGCATGATGATTTGACGTCATCCCCACCTTCCTCCGGTTT  1164

Query  301       ATTACCGGCAGTCTCGCTAGAGTGCCCAACTGAATGATGGCAACTAACAATAGGGGTTGC  360
                 ||||||||||||||||||||||||||||||||||||||||||||||||||||||||||||
Sbjct  1163      ATTACCGGCAGTCTCGCTAGAGTGCCCAACTGAATGATGGCAACTAACAATAGGGGTTGC  1104

Query  361       GCTCGTTGCGGGACTTAACCCAACATCTCACGACACGAGCTGACGACAACCATGCACCAC  420
                 ||||||||||||||||||||||||||||||||||||||||||||||||||||||||||||
Sbjct  1103      GCTCGTTGCGGGACTTAACCCAACATCTCACGACACGAGCTGACGACAACCATGCACCAC  1044

Query  421       CTGTCACCGATGCTCCGAAGAGAAACCCTATCTCTAGGGCGGTCATCGGGATGTCAAGAC  480
                 ||||||||||||||||||||||||||||||||||||||||||||||||||||||||||||
Sbjct  1043      CTGTCACCGATGCTCCGAAGAGAAACCCTATCTCTAGGGCGGTCATCGGGATGTCAAGAC  984

Query  481       CTGGTAAGGTTCTTCGCGTTGCTTCGAATTAAACCACATGCTCCACCGCTTGTGCGGGCC  540
                 ||||||||||||||||||||||||||||||||||||||||||||||||||||||||||||
Sbjct  983       CTGGTAAGGTTCTTCGCGTTGCTTCGAATTAAACCACATGCTCCACCGCTTGTGCGGGCC  924

Query  541       CCCGTCAATTCCTTTGAGTTTCAACCTTGCGGTCGTACTCCCCAGGCGGAGTGCTTAATG  600
                 ||||||||||||||||||||||||||||||||||||||||||||||||||||||||||||
Sbjct  923       CCCGTCAATTCCTTTGAGTTTCAACCTTGCGGTCGTACTCCCCAGGCGGAGTGCTTAATG  864

Query  601       CGTTAGCTGCGGCACTGAGTCCCGGAAAGGACCCAACACCTAGCACTCATCGTTTACGGC  660
                 ||||||||||||||||||||||||||||||||||||||||||||||||||||||||||||
Sbjct  863       CGTTAGCTGCGGCACTGAGTCCCGGAAAGGACCCAACACCTAGCACTCATCGTTTACGGC  804

Query  661       GTGGACTACCAGGGTATCTAATCCTGTTCGCTCCCCACGCTTTCGAGCCTCAGCGTCAGT  720
                 ||||||||||||||||||||||||||||||||||||||||||||||||||||||||||||
Sbjct  803       GTGGACTACCAGGGTATCTAATCCTGTTCGCTCCCCACGCTTTCGAGCCTCAGCGTCAGT  744

Query  721       TACAGACCAGAGAGCCGCTTTCGCCACCGGTGTTCCTCCATATATCTACGCATTTCACCG  780
                 ||||||||||||||||||||||||||||||||||||||||||||||||||||||||||||
Sbjct  743       TACAGACCAGAGAGCCGCTTTCGCCACCGGTGTTCCTCCATATATCTACGCATTTCACCG  684

Query  781       CTACACATGGAATTCCACTCTCCCCTTCTGCACTCAAGTTTGACAGTTTCCAAAGCGTAC  840
                 ||||||||||||||||||||||||||||||||||||||||||||||||||||||||||||
Sbjct  683       CTACACATGGAATTCCACTCTCCCCTTCTGCACTCAAGTTTGACAGTTTCCAAAGCGTAC  624

Query  841       TATGGTTAAGCCACAGCCTTTTACTTCAGACTTATCAAACCGCCTGCGCTCGCTTTACGC  900
                 ||||||||||||||||||||||||||||||||||||||||||||||||||||||||||||
Sbjct  623       TATGGTTAAGCCACAGCCTTTTACTTCAGACTTATCAAACCGCCTGCGCTCGCTTTACGC  564

Query  901       CCAATAAATCCGGACAACGCTCGGGACCTACGTATTACCGCGGCTGCTGGCACGTAGTTA  960
                 ||||||||||||||||||||||||||||||||||||||||||||||||||||||||||||
Sbjct  563       CCAATAAATCCGGACAACGCTCGGGACCTACGTATTACCGCGGCTGCTGGCACGTAGTTA  504

Query  961       GCCGTCCCTTTCTGGTAAGATACCGTCAA-GTGAGAAACTTTCCACTCTTCTCACA-GTT  1018
                 ||||||||||||||||||||||||||||  ||| ||| ||||||||||| | |||| |||
```

```
Sbjct  503   GCCGTCCCTTTCTGGTAAGATACCGTCACTGTGTGAA-CTTTCCACTCT-CACACACGTT  446

Query  1019  CTTCTCTTACAACAGAGCTTTACGATCCGAAAACCTTCTTCACTCACGCGGCGTTGCTCG  1078
             ||||||||||||||||||||||||||||||||||||||||||||||||||||||||||||
Sbjct  445   CTTCTCTTACAACAGAGCTTTACGATCCGAAAACCTTCTTCACTCACGCGGCGTTGCTCG  386

Query  1079  GTCAGGGTTGCCCCCATTGCCGAAGATTCCCTACTGCTGCCTCCCGTAGGAGTCTGGGCC  1138
             ||||||||| ||||||||||||||||||||||||||||||||||||||||||||||||||
Sbjct  385   GTCAGGGTTCCCCCCATTGCCGAAGATTCCCTACTGCTGCCTCCCGTAGGAGTCTGGGCC  326

Query  1139  GTGTCTCAGTCCCAGTGTGGCCGATCACCCTCTCAGGTCGGCTATGTATCGAAGCCTTGG  1198
             ||||||||||||||||||||||||||||||||||||||||||||||||||||||||||||
Sbjct  325   GTGTCTCAGTCCCAGTGTGGCCGATCACCCTCTCAGGTCGGCTATGTATCGAAGCCTTGG  266

Query  1199  TGAGCCGTTACCTCACCAACTAGCTAATACAACGCAGGTCCATCTCATAGTGAAGCAATT  1258
             |||||||||||| |||||||||||||||||||||||||||||||||||||||||||| ||
Sbjct  265   TGAGCCGTTACCCCACCAACTAGCTAATACAACGCAGGTCCATCTCATAGTGAAGCAGTT  206

Query  1259  GCTCCTTTCAAATATCTACCATGCGGTAAATACTGTTATGCGGTATTAGCTATCGTTTCC  1318
             |||||||||||  || ||||||||| ||||||||||||||||||||||||||||||||||
Sbjct  205   GCTCCTTTCAAGCATTTACCATGCGATAAATACTGTTATGCGGTATTAGCTATCGTTTCC  146

Query  1319  AATAGTTATCCCCCGCTATGAGGCAGGTTACCTACGCGTTACTCACCCGTTCGCAACTCA  1378
             ||||||||||||||||||||||| |||||||||||||||||||||||||||||| |||||
Sbjct  145   AATAGTTATCCCCCGCTATGAGGTAGGTTACCTACGCGTTACTCACCCGTTCGCGACTCA  86

Query  1379  TCCGTC-TAGTG---CAAGCACCAG-A-CT-TCA-GCGTTCTACTTGCATGTATTAGGCA  1430
             |   |  | |||   ||||| || | | |  ||| |||||| ||||||||||||||||||
Sbjct  85    TGATTAATGGTGGAGCAAGCTCCGGTATCAATCATGCGTTCCACTTGCATGTATTAGGCA  26

Query  1431  CGCCGCCAGCGTTCGTCCTGAGCCA  1455
             |||||||||||||||||||||||||
Sbjct  25    CGCCGCCAGCGTTCGTCCTGAGCCA  1
```

Streptococcus suis strain ATCC 43765 16S ribosomal RNA, partial sequence
Sequence ID: **NR_117504.1** Length: 1336 Number of Matches: 1
Range 1: 1 to 1336

Score	Expect	Identities	Gaps	Strand	Frame
2462 bits(1333)	0.0()	1336/1337(99%)	1/1337(0%)	Plus/Minus	

```
Query  100   GCGGCGTGCTGATCCGCGATTACTAGCGATTCCGACTTCATGTAGGCGAGTTGCAGCCTA  159
             ||||||||||||||||||||||||||||||||||||||||||||||||||||||||||||
Sbjct  1336  GCGGCGTGCTGATCCGCGATTACTAGCGATTCCGACTTCATGTAGGCGAGTTGCAGCCTA  1277

Query  160   CAATCCGAACTGAGACTGGCTTTAAGAGATTAGCTTGCCGTCACCGACTTGCGACTCGTT  219
             ||||||||||||||||||||||||||||||||||||||||||||||||||||||||||||
Sbjct  1276  CAATCCGAACTGAGACTGGCTTTAAGAGATTAGCTTGCCGTCACCGACTTGCGACTCGTT  1217

Query  220   GTACCAGCCATTGTAGCACGTGTGTAGCCCAGGTCATAAGGGGCATGATGATTTGACGTC  279
             ||||||||||||||||||||||||||||||||||||||||||||||||||||||||||||
Sbjct  1216  GTACCAGCCATTGTAGCACGTGTGTAGCCCAGGTCATAAGGGGCATGATGATTTGACGTC  1157

Query  280   ATCCCCACCTTCCTCCGGTTTATTACCGGCAGTCTCGCTAGAGTGCCCAACTGAATGATG  339
             ||||||||||||||||||||||||||||||||||||||||||||||||||||||||||||
Sbjct  1156  ATCCCCACCTTCCTCCGGTTTATTACCGGCAGTCTCGCTAGAGTGCCCAACTGAATGATG  1097

Query  340   GCAACTAACAATAGGGGTTGCGCTCGTTGCGGGACTTAACCCAACATCTCACGACACGAG  399
             ||||||||||||||||||||||||||||||||||||||||||||||||||||||||||||
Sbjct  1096  GCAACTAACAATAGGGGTTGCGCTCGTTGCGGGACTTAACCCAACATCTCACGACACGAG  1037

Query  400   CTGACGACAACCATGCACCACCTGTCACCGATGCTCCGAAGAGAAACCCTATCTCTAGGG  459
             ||||||||||||||||||||||||||||||||||||||||||||||||||||||||||||
Sbjct  1036  CTGACGACAACCATGCACCACCTGTCACCGATGCTCCGAAGAGAAACCCTATCTCTAGGG  977

Query  460   CGGTCATCGGGATGTCAAGACCTGGTAAGGTTCTTCGCGTTGCTTCGAATTAAACCACAT  519
             ||||||||||||||||||||||||||||||||||||||||||||||||||||||||||||
Sbjct  976   CGGTCATCGGGATGTCAAGACCTGGTAAGGTTCTTCGCGTTGCTTCGAATTAAACCACAT  917

Query  520   GCTCCACCGCTTGTGCGGGCCCCCGTCAATTCCTTTGAGTTTCAACCTTGCGGTCGTACT  579
             ||||||||||||||||||||||||||||||||||||||||||||||||||||||||||||
Sbjct  916   GCTCCACCGCTTGTGCGGGCCCCCGTCAATTCCTTTGAGTTTCAACCTTGCGGTCGTACT  857

Query  580   CCCCAGGCGGAGTGCTTAATGCGTTAGCTGCGGCACTGAGTCCCGGAAAGGACCCAACAC  639
             ||||||||||||||||||||||||||||||||||||||||||||||||||||||||||||
Sbjct  856   CCCCAGGCGGAGTGCTTAATGCGTTAGCTGCGGCACTGAGTCCCGGAAAGGACCCAACAC  797

Query  640   CTAGCACTCATCGTTTACGGCGTGGACTACCAGGGTATCTAATCCTGTTCGCTCCCCACG  699
             ||||||||||||||||||||||||||||||||||||||||||||||||||||||||||||
Sbjct  796   CTAGCACTCATCGTTTACGGCGTGGACTACCAGGGTATCTAATCCTGTTCGCTCCCCACG  737

Query  700   CTTTCGAGCCTCAGCGTCAGTTACAGACCAGAGAGCCGCTTTCGCCACCGGTGTTCCTCC  759
             ||||||||||||||||||||||||||||||||||||||||||||||||||||||||||||
Sbjct  736   CTTTCGAGCCTCAGCGTCAGTTACAGACCAGAGAGCCGCTTTCGCCACCGGTGTTCCTCC  677

Query  760   ATATATCTACGCATTTCACCGCTACACATGGAATTCCACTCTCCCCTTCTGCACTCAAGT  819
             ||||||||||||||||||||||||||||||||||||||||||||||||||||||||||||
Sbjct  676   ATATATCTACGCATTTCACCGCTACACATGGAATTCCACTCTCCCCTTCTGCACTCAAGT  617

Query  820   TTGACAGTTTCCAAAGCGTACTATGGTTAAGCCACAGCCTTTTACTTCAGACTTATCAAA  879
             ||||||||||||||||||||||||||||||||||||||||||||||||||||||||||||
Sbjct  616   TTGACAGTTTCCAAAGCGTACTATGGTTAAGCCACAGCCTTTTACTTCAGACTTATCAAA  557

Query  880   CCGCCTGCGCTCGCTTTACGCCCAATAAATCCGGACAACGCTCGGGACCTACGTATTACC  939
             ||||||||||||||||||||||||||||||||||||||||||||||||||||||||||||
Sbjct  556   CCGCCTGCGCTCGCTTTACGCCCAATAAATCCGGACAACGCTCGGGACCTACGTATTACC  497

Query  940   GCGGCTGCTGGCACGTAGTTAGCCGTCCCTTTCTGGTAAGATACCGTCAAGTGAGAAACT  999
             ||||||||||||||||||||||||||||||||||||||||||||||||||||||||||||
Sbjct  496   GCGGCTGCTGGCACGTAGTTAGCCGTCCCTTTCTGGTAAGATACCGTCAAGTGAGAAACT  437

Query  1000  TTCCACTCTTCTCACAGTTCTTCTCTTACAACAGAGCTTTACGATCCGAAAACCTTCTTC  1059
             ||||||||||||||||||||||||||||||||||||||||||||||||||||||||||||
Sbjct  436   TTCCACTCTTCTCACAGTTCTTCTCTTACAACAGAGCTTTACGATCCGAAAACCTTCTTC  377

Query  1060  ACTCACGCGGCGTTGCTCGGTCAGGGTTGCCCCCATTGCCGAAGATTCCCTACTGCTGCC  1119
             ||||||||||||||||||||||||||||||||||||||||||||||||||||||||||||
Sbjct  376   ACTCACGCGGCGTTGCTCGGTCAGGGTTGCCCCCATTGCCGAAGATTCCCTACTGCTGCC  317

Query  1120  TCCCGTAGGAGTCTGGGCCGTGTCTCAGTCCCAGTGTGGCCGATCACCCTCTCAGGTCGG  1179
             ||||||||||||||||||||||||||||||||||||||||||||||||||||||||||||
Sbjct  316   TCCCGTAGGAGTCTGGGCCGTGTCTCAGTCCCAGTGTGGCCGATCACCCTCTCAGGTCGG  257

Query  1180  CTATGTATCGAAGCCTTGGTGAGCCGTTACCTCACCAACTAGCTAATACAACGCAGGTCC  1239
             ||||||||||||||||||||||||||||||||||||||||||||||||||||||||||||
Sbjct  256   CTATGTATCGAAGCCTTGGTGAGCCGTTACCTCACCAACTAGCTAATACAACGCAGGTCC  197

Query  1240  ATCTCATAGTGAAGCAATTGCTCCTTTCAAATATCTACCATGCGGTAAATACTGTTATGC  1299
             ||||||||||||||||||||||||||||||||||||||||||||||||||||||||||||
Sbjct  196   ATCTCATAGTGAAGCAATTGCTCCTTTCAAATATCTACCATGCGGTAAATACTGTTATGC  137

Query  1300  GGTATTAGCTATCGTTTCCAATAGTTATCCCCCGCTATGAGGCAGGTTACCTACGCGTTA  1359
             ||||||||||||||||||||||||||||||||||||||||||||||||||||||||||||
Sbjct  136   GGTATTAGCTATCGTTTCCAATAGTTATCCCCCGCTATGAGGCAGGTTACCTACGCGTTA  77
```

```
Query  1360  CTCACCCGTTCGCAACTCATCCGTCTAGTGCAAGCACCAGACTTCAGCGTTCTACTTGCA  1419
             ||||||||||||||||||||||||||||||||||||||||||||||||||||||||||||
Sbjct  76    CTCACCCGTTCGCAACTCATCCGTCTAGTGCAAGCACCAGACTTCAGCGTTCTACTTGCA  17

Query  1420  TGTATTAGGCACGCCGC  1436
             ||||| |||||||||||
Sbjct  16    TGTAT-AGGCACGCCGC  1
```

Streptococcus oriscaviae strain HKU75 16S ribosomal RNA, complete sequence

Sequence ID: **NR_181935.1** Length: 1557 Number of Matches: 1

Range 1: 8 to 1482

Score	Expect	Identities	Gaps	Strand	Frame
2435 bits(1318)	0.0()	1427/1478(97%)	14/1478(0%)	Plus/Minus	

```
Query  1     AGGCGGCTGGCTCCCAAAAGGTTACCTCACCGACTTCGGGTGTTACAAACTCTCGTGGTG  60
             |||||||||||||| || ||||||||||||||||||||||||||||||||||||||||||
Sbjct  1482  AGGCGGCTGGCTCCTAATAGGTTACCTCACCGACTTCGGGTGTTACAAACTCTCGTGGTG  1423

Query  61    TGACGGGCGGTGTGTACAAGGCCCGGGAACGTATTCACCGCGGCGTGCTGATCCGCGATT  120
             ||||||||||||||||||||||||||||||||||||||||||||||||||||||||||||
Sbjct  1422  TGACGGGCGGTGTGTACAAGGCCCGGGAACGTATTCACCGCGGCGTGCTGATCCGCGATT  1363

Query  121   ACTAGCGATTCCGACTTCATGTAGGCGAGTTGCAGCCTACAATCCGAACTGAGACTGGCT  180
             ||||||||||||||||||||||||||||||||||||||||||||||||||||||||||||
Sbjct  1362  ACTAGCGATTCCGACTTCATGTAGGCGAGTTGCAGCCTACAATCCGAACTGAGACTGGCT  1303

Query  181   TTAAGAGATTAGCTTGCCGTCACCGACTTGCGACTCGTTGTACCAGCCATTGTAGCACGT  240
             ||||||||||||||||||||||||||||||||||||||||||||||||||||||||||||
Sbjct  1302  TTAAGAGATTAGCTTGCCGTCACCGACTTGCGACTCGTTGTACCAGCCATTGTAGCACGT  1243

Query  241   GTGTAGCCCAGGTCATAAGGGGCATGATGATTTGACGTCATCCCCACCTTCCTCCGGTTT  300
             ||||||||||||||||||||||||||||||||||||||||||||||||||||||||||||
Sbjct  1242  GTGTAGCCCAGGTCATAAGGGGCATGATGATTTGACGTCATCCCCACCTTCCTCCGGTTT  1183

Query  301   ATTACCGGCAGTCTCGCTAGAGTGCCCAACTGAATGATGGCAACTAACAATAGGGGTTGC  360
             ||||||||||||||||||||||||||||||| ||||||||||||||||||||||||||||
Sbjct  1182  ATTACCGGCAGTCTCGCTAGAGTGCCCAACTTAATGATGGCAACTAACAATAGGGGTTGC  1123

Query  361   GCTCGTTGCGGGACTTAACCCAACATCTCACGACACGAGCTGACGACAACCATGCACCAC  420
             ||||||||||||||||||||||||||||||||||||||||||||||||||||||||||||
Sbjct  1122  GCTCGTTGCGGGACTTAACCCAACATCTCACGACACGAGCTGACGACAACCATGCACCAC  1063

Query  421   CTGTCACCGATGCTCCGAAGAGAAACCCTATCTCTAGGGCGGTCATCGGGATGTCAAGAC  480
             ||||||||||||  ||||||  |||| |||||||||| |||| |||||||||||||||||
Sbjct  1062  CTGTCACCGATGTACCGAAGTAAAACTCTATCTCTAGAGCGGGCATCGGGATGTCAAGAC  1003

Query  481   CTGGTAAGGTTCTTCGCGTTGCTTCGAATTAAACCACATGCTCCACCGCTTGTGCGGGCC  540
             ||||||||||||||||||||||||||||||||||||||||||||||||||||||||||||
Sbjct  1002  CTGGTAAGGTTCTTCGCGTTGCTTCGAATTAAACCACATGCTCCACCGCTTGTGCGGGCC  943

Query  541   CCCGTCAATTCCTTTGAGTTTCAACCTTGCGGTCGTACTCCCCAGGCGGAGTGCTTAATG  600
             ||||||||||||||||||||||| |||||||| |||||||||||||||||||||||||||
Sbjct  942   CCCGTCAATTCCTTTGAGTTTCAGCCTTGCGGCCGTACTCCCCAGGCGGAGTGCTTAATG  883

Query  601   CGTTAGCTGCGGCACTGAGTCCCGGAAAGGACCCAACACCTAGCACTCATCGTTTACGGC  660
             ||||||||||||||||||||||||||||||||||||||||||||||||||||||||||||
Sbjct  882   CGTTAGCTGCGGCACTGAGTCCCGGAAAGGACCCAACACCTAGCACTCATCGTTTACGGC  823

Query  661   GTGGACTACCAGGGTATCTAATCCTGTTCGCTCCCCACGCTTTCGAGCCTCAGCGTCAGT  720
             |||||||||||||||||||||||||||||||| |||||||||||||||||||||||||||
Sbjct  822   GTGGACTACCAGGGTATCTAATCCTGTTCGCTACCCACGCTTTCGAGCCTCAGCGTCAGT  763

Query  721   TACAGACCAGAGAGCCGCTTTCGCCACCGGTGTTCCTCCATATATCTACGCATTTCACCG  780
             ||||||||||||||||||||||||||||||||||||||||||||||||||||||||||||
Sbjct  762   TACAGACCAGAGAGCCGCTTTCGCCACCGGTGTTCCTCCATATATCTACGCATTTCACCG  703

Query  781   CTACACATGGAATTCCACTCTCCCCTTCTGCACTCAAGTTTGACAGTTTCCAAAGCGTAC  840
             ||||||||||||||||||||||||||||||||||||||||||||||||||||||||||||
Sbjct  702   CTACACATGGAATTCCACTCTCCCCTTCTGCACTCAAGTTTGACAGTTTCCAAAGCGTAC  643

Query  841   TATGGTTAAGCCACAGCCTTTTACTTCAGACTTATCAAACCGCCTGCGCTCGCTTTACGC  900
             ||||||||||||||||||||||||||||||||||||||||||||||||||||||||||||
Sbjct  642   TATGGTTAAGCCACAGCCTTTTACTTCAGACTTATCAAACCGCCTGCGCTCGCTTTACGC  583

Query  901   CCAATAAATCCGGACAACGCTCGGGACCTACGTATTACCGCGGCTGCTGGCACGTAGTTA  960
             ||||||||||||||||||||||||||||||||||||||||||||||||||||||||||||
Sbjct  582   CCAATAAATCCGGACAACGCTCGGGACCTACGTATTACCGCGGCTGCTGGCACGTAGTTA  523

Query  961   GCCGTCCCTTTCTGGTAAGATACCGTCAA-GTGAGAAACTTTCCACTCTTCTCACA-GTT  1018
             ||||||||||||||||||| ||||||||  ||| ||| ||||||||||| | |||  |||
Sbjct  522   GCCGTCCCTTTCTGGTAAGTTACCGTCACTGTGTGAA-CTTTCCACTCT-CACACTCGTT  465

Query  1019  CTTCTCTTACAACAGAGCTTTACGATCCGAAAACCTTCTTCACTCACGCGGCGTTGCTCG  1078
             ||||||||||||||||||||||||||||||||||||||||||||||||||||||||||||
Sbjct  464   CTTCTCTTACAACAGAGCTTTACGATCCGAAAACCTTCTTCACTCACGCGGCGTTGCTCG  405

Query  1079  GTCAGGGTTGCCCCCATTGCCGAAGATTCCCTACTGCTGCCTCCCGTAGGAGTCTGGGCC  1138
             ||||||||| ||||||||||||||||||||||||||||||||||||||||||||||||||
Sbjct  404   GTCAGGGTTCCCCCCATTGCCGAAGATTCCCTACTGCTGCCTCCCGTAGGAGTCTGGGCC  345

Query  1139  GTGTCTCAGTCCCAGTGTGGCCGATCACCCTCTCAGGTCGGCTATGTATCGAAGCCTTGG  1198
             |||||||||||||||||||||||||||||||||||||||||||||||||||  |||||||
Sbjct  344   GTGTCTCAGTCCCAGTGTGGCCGATCACCCTCTCAGGTCGGCTATGTATCGTCGCCTTGG  285

Query  1199  TGAGCCGTTACCTCACCAACTAGCTAATACAACGCAGGTCCATCTCATAGTGAAGCAATT  1258
             |||||||||||| |||||||||||||||||||||||||||||||||||||||||||| ||
Sbjct  284   TGAGCCGTTACCCCACCAACTAGCTAATACAACGCAGGTCCATCTCATAGTGAAGCATTT  225

Query  1259  GCTCCTTTCAAATATCTACCATGCGGTAAATACTGTTATGCGGTATTAGCTATCGTTTCC  1318
             ||||||||||| ||| ||||||||| ||||||||||||||||||||||||||||||||||
Sbjct  224   GCTCCTTTCAAGTATTTACCATGCGATAAATACTGTTATGCGGTATTAGCTATCGTTTCC  165

Query  1319  AATAGTTATCCCCCGCTATGAGGCAGGTTACCTACGCGTTACTCACCCGTTCGCAACTCA  1378
             |||||||||||||||||||||||||||||||||||||||||||||||||||||| |||||
Sbjct  164   AATAGTTATCCCCCGCTATGAGGCAGGTTACCTACGCGTTACTCACCCGTTCGCGACTCA  105

Query  1379  TCC-GTCTAGTG---CAAGCACCAG-A-CTTC-A-GCGTTCTACTTGCATGTATTAGGCA  1430
             |   |  | |||   ||||| || | | |  | | |||||| ||||||||||||||||||
Sbjct  104   TGATGAATGGTGGAGCAAGCTCCGGTATCCACCATGCGTTCCACTTGCATGTATTAGGCA  45

Query  1431  CGCCGCCAGCGTTCGTCCTGAGCCAG-ATCCAAACTCT  1467
             |||||||||||||||||||||||||| ||| |||||||
Sbjct  44    CGCCGCCAGCGTTCGTCCTGAGCCAGGATC-AAACTCT  8
```

Streptococcus oriscaviae strain HKU75 16S ribosomal RNA, partial sequence

Sequence ID: **NR_181481.1** Length: 1512 Number of Matches: 1

Range 1: 1 to 1475

Score	Expect	Identities	Gaps	Strand	Frame
2435 bits(1318)	0.0()	1427/1478(97%)	14/1478(0%)	Plus/Minus	

```
Query  1     AGGCGGCTGGCTCCCAAAAGGTTACCTCACCGACTTCGGGTGTTACAAACTCTCGTGGTG  60
Sbjct  1475  AGGCGGCTGGCTCCTAATAGGTTACCTCACCGACTTCGGGTGTTACAAACTCTCGTGGTG  1416

Query  61    TGACGGGCGGTGTGTACAAGGCCCGGGAACGTATTCACCGCGGCGTGCTGATCCGCGATT  120
Sbjct  1415  TGACGGGCGGTGTGTACAAGGCCCGGGAACGTATTCACCGCGGCGTGCTGATCCGCGATT  1356

Query  121   ACTAGCGATTCCGACTTCATGTAGGCGAGTTGCAGCCTACAATCCGAACTGAGACTGGCT  180
Sbjct  1355  ACTAGCGATTCCGACTTCATGTAGGCGAGTTGCAGCCTACAATCCGAACTGAGACTGGCT  1296

Query  181   TTAAGAGATTAGCTTGCCGTCACCGACTTGCGACTCGTTGTACCAGCCATTGTAGCACGT  240
Sbjct  1295  TTAAGAGATTAGCTTGCCGTCACCGACTTGCGACTCGTTGTACCAGCCATTGTAGCACGT  1236

Query  241   GTGTAGCCCAGGTCATAAGGGGCATGATGATTTGACGTCATCCCCACCTTCCTCCGGTTT  300
Sbjct  1235  GTGTAGCCCAGGTCATAAGGGGCATGATGATTTGACGTCATCCCCACCTTCCTCCGGTTT  1176

Query  301   ATTACCGGCAGTCTCGCTAGAGTGCCCAACTGAATGATGGCAACTAACAATAGGGGTTGC  360
Sbjct  1175  ATTACCGGCAGTCTCGCTAGAGTGCCCAACTTAATGATGGCAACTAACAATAGGGGTTGC  1116

Query  361   GCTCGTTGCGGGACTTAACCCAACATCTCACGACACGAGCTGACGACAACCATGCACCAC  420
Sbjct  1115  GCTCGTTGCGGGACTTAACCCAACATCTCACGACACGAGCTGACGACAACCATGCACCAC  1056

Query  421   CTGTCACCGATGCTCCGAAGAGAAACCCTATCTCTAGGGCGGTCATCGGGATGTCAAGAC  480
Sbjct  1055  CTGTCACCGATGTACCGAAGTAAAACTCTATCTCTAGAGCGGGCATCGGGATGTCAAGAC  996

Query  481   CTGGTAAGGTTCTTCGCGTTGCTTCGAATTAAACCACATGCTCCACCGCTTGTGCGGGCC  540
Sbjct  995   CTGGTAAGGTTCTTCGCGTTGCTTCGAATTAAACCACATGCTCCACCGCTTGTGCGGGCC  936

Query  541   CCCGTCAATTCCTTTGAGTTTCAACCTTGCGGTCGTACTCCCCAGGCGGAGTGCTTAATG  600
Sbjct  935   CCCGTCAATTCCTTTGAGTTTCAGCCTTGCGGCCGTACTCCCCAGGCGGAGTGCTTAATG  876

Query  601   CGTTAGCTGCGGCACTGAGTCCCGGAAAGGACCCAACACCTAGCACTCATCGTTTACGGC  660
Sbjct  875   CGTTAGCTGCGGCACTGAGTCCCGGAAAGGACCCAACACCTAGCACTCATCGTTTACGGC  816

Query  661   GTGGACTACCAGGGTATCTAATCCTGTTCGCTCCCCACGCTTTCGAGCCTCAGCGTCAGT  720
Sbjct  815   GTGGACTACCAGGGTATCTAATCCTGTTCGCTACCCACGCTTTCGAGCCTCAGCGTCAGT  756

Query  721   TACAGACCAGAGAGCCGCTTTCGCCACCGGTGTTCCTCCATATATCTACGCATTTCACCG  780
Sbjct  755   TACAGACCAGAGAGCCGCTTTCGCCACCGGTGTTCCTCCATATATCTACGCATTTCACCG  696

Query  781   CTACACATGGAATTCCACTCTCCCCTTCTGCACTCAAGTTTGACAGTTTCCAAAGCGTAC  840
Sbjct  695   CTACACATGGAATTCCACTCTCCCCTTCTGCACTCAAGTTTGACAGTTTCCAAAGCGTAC  636

Query  841   TATGGTTAAGCCACAGCCTTTTACTTCAGACTTATCAAACCGCCTGCGCTCGCTTTACGC  900
Sbjct  635   TATGGTTAAGCCACAGCCTTTTACTTCAGACTTATCAAACCGCCTGCGCTCGCTTTACGC  576

Query  901   CCAATAAATCCGGACAACGCTCGGGACCTACGTATTACCGCGGCTGCTGGCACGTAGTTA  960
Sbjct  575   CCAATAAATCCGGACAACGCTCGGGACCTACGTATTACCGCGGCTGCTGGCACGTAGTTA  516

Query  961   GCCGTCCCTTTCTGGTAAGATACCGTCAA-GTGAGAAACTTTCCACTCTTCTCACA-GTT  1018
Sbjct  515   GCCGTCCCTTTCTGGTAAGTTACCGTCACTGTGTGAA-CTTTCCACTCT-CACACTCGTT  458

Query  1019  CTTCTCTTACAACAGAGCTTTACGATCCGAAAACCTTCTTCACTCACGCGGCGTTGCTCG  1078
Sbjct  457   CTTCTCTTACAACAGAGCTTTACGATCCGAAAACCTTCTTCACTCACGCGGCGTTGCTCG  398

Query  1079  GTCAGGGTTGCCCCCATTGCCGAAGATTCCCTACTGCTGCCTCCCGTAGGAGTCTGGGCC  1138
Sbjct  397   GTCAGGGTTCCCCCCATTGCCGAAGATTCCCTACTGCTGCCTCCCGTAGGAGTCTGGGCC  338

Query  1139  GTGTCTCAGTCCCAGTGTGGCCGATCACCCTCTCAGGTCGGCTATGTATCGAAGCCTTGG  1198
Sbjct  337   GTGTCTCAGTCCCAGTGTGGCCGATCACCCTCTCAGGTCGGCTATGTATCGTCGCCTTGG  278

Query  1199  TGAGCCGTTACCTCACCAACTAGCTAATACAACGCAGGTCCATCTCATAGTGAAGCAATT  1258
Sbjct  277   TGAGCCGTTACCCCACCAACTAGCTAATACAACGCAGGTCCATCTCATAGTGAAGCATTT  218

Query  1259  GCTCCTTTCAAATATCTACCATGCGGTAAATACTGTTATGCGGTATTAGCTATCGTTTCC  1318
Sbjct  217   GCTCCTTTCAAGTATTTACCATGCGATAAATACTGTTATGCGGTATTAGCTATCGTTTCC  158

Query  1319  AATAGTTATCCCCCGCTATGAGGCAGGTTACCTACGCGTTACTCACCCGTTCGCAACTCA  1378
Sbjct  157   AATAGTTATCCCCCGCTATGAGGCAGGTTACCTACGCGTTACTCACCCGTTCGCGACTCA  98

Query  1379  TCC-GTCTAGTG---CAAGCACCAG-A-CTTC-A-GCGTTCTACTTGCATGTATTAGGCA  1430
Sbjct  97    TGATGAATGGTGGAGCAAGCTCCGGTATCCACCATGCGTTCCACTTGCATGTATTAGGCA  38

Query  1431  CGCCGCCAGCGTTCGTCCTGAGCCAG-ATCCAAACTCT  1467
Sbjct  37    CGCCGCCAGCGTTCGTCCTGAGCCAGGATC-AAACTCT  1
```

Streptococcus respiraculi strain HTS25 16S ribosomal RNA, partial sequence

Sequence ID: **NR_179720.1** Length: 1510 Number of Matches: 1

Range 1: 1 to 1467

Score	Expect	Identities	Gaps	Strand	Frame
2427 bits(1314)	0.0()	1420/1471(97%)	8/1471(0%)	Plus/Minus	

```
Query  1     AGGCGGCTGGCTCCCAAAAGGTTACCTCACCGACTTCGGGTGTTACAAACTCTCGTGGTG  60
Sbjct  1467  AGGCGGCTGGCTCCTAAAAGGTTACCTCACCGACTTCGGGTGTTACAAACTCTCGTGGTG  1408

Query  61    TGACGGGCGGTGTGTACAAGGCCCGGGAACGTATTCACCGCGGCGTGCTGATCCGCGATT  120
Sbjct  1407  TGACGGGCGGTGTGTACAAGGCCCGGGAACGTATTCACCGCGGCGTGCTGATCCGCGATT  1348

Query  121   ACTAGCGATTCCGACTTCATGTAGGCGAGTTGCAGCCTACAATCCGAACTGAGACTGGCT  180
Sbjct  1347  ACTAGCGATTCCGACTTCATGTAGGCGAGTTGCAGCCTACAATCCGAACTGAGACTGGCT  1288
```

```
Query  181   TTAAGAGATTAGCTTGCCGTCACCGACTTGCGACTCGTTGTACCAGCCATTGTAGCACGT  240
Sbjct  1287  TTAAGAGATTAGCTTGCCGTCACCGACTCGCGACTCGTTGTACCAGCCATTGTAGCACGT  1228
Query  241   GTGTAGCCCAGGTCATAAGGGGCATGATGATTTGACGTCATCCCCACCTTCCTCCGGTTT  300
Sbjct  1227  GTGTAGCCCAGGTCATAAGGGGCATGATGATTTGACGTCATCCCCACCTTCCTCCGGTTT  1168
Query  301   ATTACCGGCAGTCTCGCTAGAGTGCCCAACTGAATGATGGCAACTAACAATAGGGGTTGC  360
Sbjct  1167  ATTACCGGCAGTCTCGCTAGAGTGCCCAACTCAATGATGGCAACTAACAATAGGGGTTGC  1108
Query  361   GCTCGTTGCGGGACTTAACCCAACATCTCACGACACGAGCTGACGACAACCATGCACCAC  420
Sbjct  1107  GCTCGTTGCGGGACTTAACCCAACATCTCACGACACGAGCTGACGACAACCATGCACCAC  1048
Query  421   CTGTCACCGATGCTCCGAAGAGAAACCCTATCTCTAGGGCGGTCATCGGGATGTCAAGAC  480
Sbjct  1047  CTGTCACCAGTGTTCCGAAGAAAAATCCTATCTCTAGGACGGTCACTGGGATGTCAAGAC  988
Query  481   CTGGTAAGGTTCTTCGCGTTGCTTCGAATTAAACCACATGCTCCACCGCTTGTGCGGGCC  540
Sbjct  987   CTGGTAAGGTTCTTCGCGTTGCTTCGAATTAAACCACATGCTCCACCGCTTGTGCGGGCC  928
Query  541   CCCGTCAATTCCTTTGAGTTTCAACCTTGCGGTCGTACTCCCCAGGCGGAGTGCTTAATG  600
Sbjct  927   CCCGTCAATTCCTTTGAGTTTCAACCTTGCGGTCGTACTCCCCAGGCGGAGTGCTTAATG  868
Query  601   CGTTAGCTGCGGCACTGAGTCCCGGAAAGGACCCAACACCTAGCACTCATCGTTTACGGC  660
Sbjct  867   CGTTAGCTGCGGCACTGAGTCCCGGAAAGGACCCAACACCTAGCACTCATCGTTTACGGC  808
Query  661   GTGGACTACCAGGGTATCTAATCCTGTTCGCTCCCCACGCTTTCGAGCCTCAGCGTCAGT  720
Sbjct  807   GTGGACTACCAGGGTATCTAATCCTGTTCGCTCCCCACGCTTTCGAGCCTCAGCGTCAGT  748
Query  721   TACAGACCAGAGAGCCGCTTTCGCCACCGGTGTTCCTCCATATATCTACGCATTTCACCG  780
Sbjct  747   TACAGACCAGAGAGCCGCTTTCGCCACCGGTGTTCCTCCATATATCTACGCATTTCACCG  688
Query  781   CTACACATGGAATTCCACTCTCCCCTTCTGCACTCAAGTTTGACAGTTTCCAAAGCGTAC  840
Sbjct  687   CTACACATGGAATTCCACTCCCCCCTTCTGCACTCAAGTTTGACAGTTTCAAAAGCGTAC  628
Query  841   TATGGTTAAGCCACAGCCTTTTACTTCAGACTTATCAAACCGCCTGCGCTCGCTTTACGC  900
Sbjct  627   TATGGTTGAGCCACAGCCTTTTACTTCTGACTTATCAAACCGCCTGCGCTCGCTTTACGC  568
Query  901   CCAATAAATCCGGACAACGCTCGGGACCTACGTATTACCGCGGCTGCTGGCACGTAGTTA  960
Sbjct  567   CCAATAAATCCGGACAACGCTCGGGACCTACGTATTACCGCGGCTGCTGGCACGTAGTTA  508
Query  961   GCCGTCCCTTTCTGGTAAGATACCGTCAAGT-GAGAAACTTTCCACTCTT-CTCACAGTT  1018
Sbjct  507   GCCGTCCCTTTCTGGTAAGTTACCGTCACTTAGCGGAT-TTTCCACTCCCGCTAAC-GTT  450
Query  1019  CTTCTCTTACAACAGAGCTTTACGATCCGAAAACCTTCTTCACTCACGCGGCGTTGCTCG  1078
Sbjct  449   CTTCTCTTACAACAGAGCTTTACGATCCGAAAACCTTCTTCACTCACGCGGCGTTGCTCG  390
Query  1079  GTCAGGGTTGCCCCCATTGCCGAAGATTCCCTACTGCTGCCTCCCGTAGGAGTCTGGGCC  1138
Sbjct  389   GTCAGGGTTCCCCCCATTGCCGAAGATTCCCTACTGCTGCCTCCCGTAGGAGTCTGGGCC  330
Query  1139  GTGTCTCAGTCCCAGTGTGGCCGATCACCCTCTCAGGTCGGCTATGTATCGAAGCCTTGG  1198
Sbjct  329   GTGTCTCAGTCCCAGTGTGGCCGATCACCCTCTCAGGTCGGCTATGTATCGTCGCCTTGG  270
Query  1199  TGAGCCGTTACCTCACCAACTAGCTAATACAACGCAGGTCCATCTCATAGTGAAGCAATT  1258
Sbjct  269   TGAGCCGTTACCTCACCAACTAGCTAATACAACGCAGGTCCATCTGGTAGTGGTGCAATT  210
Query  1259  GCTCCTTTCAAATATCTACCATGCGGTAAATACTGTTATGCGGTATTAGCTATCGTTTCC  1318
Sbjct  209   GCACCTTTCAAATACTTACCATGCAGTAAGTACTTTTATGCGGTATTAGCTATCGTTTCC  150
Query  1319  AATAGTTATCCCCCGCTATGAGGCAGGTTACCTACGCGTTACTCACCCGTTCGCAACTCA  1378
Sbjct  149   AATAGTTATCCCCCGCTACCAGGCAGGTTACCTACGCGTTACTCACCCGTTCGCAACTCC  90
Query  1379  TCCGTCTAG-TGCAAGCACCAGACTTCAGCGTTCTACTTGCATGTATTAGGCACGCCGCC  1437
Sbjct  89    TCCG-CTCGGTGCAAGCACCAAGCTTCAGCGTTCTACTTGCATGTATTAGGCACGCCGCC  31
Query  1438  AGCGTTCGTCCTGAGCCAG-ATCCAAACTCT  1467
Sbjct  30    AGCGTTCGTCCTGAGCCAGGATC-AAACTCT  1
```

Streptococcus gallinaceus strain CCUG 42692 16S ribosomal RNA, partial sequence
Sequence ID: **NR_025453.1** Length: 1502 Number of Matches: 1
Range 1: 1 to 1447

Score	Expect	Identities	Gaps	Strand	Frame
2401 bits(1300)	0.0()	1399/1448(97%)	2/1448(0%)	Plus/Minus	

```
Query  1     AGGCGGCTGGCTCCCAAAAGGTTACCTCACCGACTTCGGGTGTTACAAACTCTCGTGGTG  60
Sbjct  1447  AGGCGGCTGGCTCCTAAAAGGTTACCTCACCGACTTCGGGTGTTACAAACTCTCGTGGTG  1388
Query  61    TGACGGGCGGTGTGTACAAGGCCCGGGAACGTATTCACCGCGGCGTGCTGATCCGCGATT  120
Sbjct  1387  TGACGGGCGGTGTGTACAAGGCCCGGGAACGTATTCACCGCGGCGTGCTGATCCGCGATT  1328
Query  121   ACTAGCGATTCCGACTTCATGTAGGCGAGTTGCAGCCTACAATCCGAACTGAGACTGGCT  180
Sbjct  1327  ACTAGCGATTCCGACTTCATGTAGGCGAGTTGCAGCCTACAATCCGAACTGAGACTGGCT  1268
Query  181   TTAAGAGATTAGCTTGCCGTCACCGACTTGCGACTCGTTGTACCAGCCATTGTAGCACGT  240
Sbjct  1267  TTAAGAGATTAGCTTGCCGTCACCGACTTGCGACTCGTTGTACCAGCCATTGTAGCACGT  1208
Query  241   GTGTAGCCCAGGTCATAAGGGGCATGATGATTTGACGTCATCCCCACCTTCCTCCGGTTT  300
Sbjct  1207  GTGTAGCCCAGGTCATAAGGGGCATGATGATTTGACGTCATCCCCACCTTCCTCCGGTTT  1148
Query  301   ATTACCGGCAGTCTCGCTAGAGTGCCCAACTGAATGATGGCAACTAACAATAGGGGTTGC  360
Sbjct  1147  ATTACCGGCAGTCTCGCTAGAGTGCCCAACTCAATGATGGCAACTAACAATAGGGGTTGC  1088
Query  361   GCTCGTTGCGGGACTTAACCCAACATCTCACGACACGAGCTGACGACAACCATGCACCAC  420
Sbjct  1087  GCTCGTTGCGGGACTTAACCCAACATCTCACGACACGAGCTGACGACAACCATGCACCAC  1028
```

```
Query  421    CTGTCACCGATGCTCCGAAGAGAAACCCTATCTCTAGGGCGGTCATCGGGATGTCAAGAC  480
              ||||||||  ||  ||||||  ||| |||||||||||| ||||||  |||||||||||||
Sbjct  1027   CTGTCACCTCTGTCCCGAAGGAAAATCCTATCTCTAGGACGGTCAGAGGGATGTCAAGAC  968

Query  481    CTGGTAAGGTTCTTCGCGTTGCTTCGAATTAAACCACATGCTCCACCGCTTGTGCGGGCC  540
              ||||||||||||||||||||||||||||||||||||||||||||||||||||||||||||
Sbjct  967    CTGGTAAGGTTCTTCGCGTTGCTTCGAATTAAACCACATGCTCCACCGCTTGTGCGGGCC  908

Query  541    CCCGTCAATTCCTTTGAGTTTCAACCTTGCGGTCGTACTCCCCAGGCGGAGTGCTTAATG  600
              ||||||||||||||||||||||||||||||||||||||||||||||||||||||||||||
Sbjct  907    CCCGTCAATTCCTTTGAGTTTCAACCTTGCGGTCGTACTCCCCAGGCGGAGTGCTTAATG  848

Query  601    CGTTAGCTGCGGCACTGAGTCCCGGAAAGGACCCAACACCTAGCACTCATCGTTTACGGC  660
              |||||||| |||||||||||||||||||||||||||||||||||||||||||||||||||
Sbjct  847    CGTTAGCTACGGCACTGAGTCCCGGAAAGGACCCAACACCTAGCACTCATCGTTTACGGC  788

Query  661    GTGGACTACCAGGGTATCTAATCCTGTTCGCTCCCCACGCTTTCGAGCCTCAGCGTCAGT  720
              ||||||||||||||||||||||||||||||||||||||||||||||||||||||||||||
Sbjct  787    GTGGACTACCAGGGTATCTAATCCTGTTCGCTCCCCACGCTTTCGAGCCTCAGCGTCAGT  728

Query  721    TACAGACCAGAGAGCCGCTTTCGCCACCGGTGTTCCTCCATATATCTACGCATTTCACCG  780
              ||||||||||||||||||||||||||||||||||||||||||||||||||||||||||||
Sbjct  727    TACAGACCAGAGAGCCGCTTTCGCCACCGGTGTTCCTCCATATATCTACGCATTTCACCG  668

Query  781    CTACACATGGAATTCCACTCTCCCCTTCTGCACTCAAGTTTGACAGTTTCCAAAGCGTAC  840
              ||||||||||||||||||||||||||||||||||||||||  ||||||||||||||  ||
Sbjct  667    CTACACATGGAATTCCACTCTCCCCTTCTGCACTCAAGTTGTACAGTTTCCAAAGCACAC  608

Query  841    TATGGTTAAGCCACAGCCTTTTACTTCAGACTTATCAAACCGCCTGCGCTCGCTTTACGC  900
               ||||||||||||| ||||||||||||||||||||  |||||||||||||||||||||||
Sbjct  607    AATGGTTAAGCCACTGCCTTTTACTTCAGACTTATACAACCGCCTGCGCTCGCTTTACGC  548

Query  901    CCAATAAATCCGGACAACGCTCGGGACCTACGTATTACCGCGGCTGCTGGCACGTAGTTA  960
              ||||||||||||||||||||| ||||||||||||||||||||||||||||||||||||||
Sbjct  547    CCAATAAATCCGGACAACGCTTGGGACCTACGTATTACCGCGGCTGCTGGCACGTAGTTA  488

Query  961    GCCGTCCCTTTCTGGTAAGATACCGTCAAGTGAGAAACTTTCCACTCTTCTCACAGTTCT  1020
              ||||||||||||||||||||||||||||  |||| ||||||||||||| ||||  |||||
Sbjct  487    GCCGTCCCTTTCTGGTAAGATACCGTCACTTGAGTAACTTTCCACTCTACTCAACGTTCT  428

Query  1021   TCTCTTACAACAGAGCTTTACGATCCGAAAACCTTCTTCACTCACGCGGCGTTGCTCGGT  1080
              ||||||||||||||||||||||||||||||||||||||||||||||||||||||||||||
Sbjct  427    TCTCTTACAACAGAGCTTTACGATCCGAAAACCTTCTTCACTCACGCGGCGTTGCTCGGT  368

Query  1081   CAGGGTTGCCCCCATTGCCGAAGATTCCCTACTGCTGCCTCCCGTAGGAGTCTGGGCCGT  1140
              ||||||||||||||||||||||||||||||||||||||||||||||||||||||||||||
Sbjct  367    CAGGGTTGCCCCCATTGCCGAAGATTCCCTACTGCTGCCTCCCGTAGGAGTCTGGGCCGT  308

Query  1141   GTCTCAGTCCCAGTGTGGCCGATCACCCTCTCAGGTCGGCTATGTATCGAAGCCTTGGTG  1200
              |||||||||||||||||||||||||||||||||||||||||||||||||  |||||||||
Sbjct  307    GTCTCAGTCCCAGTGTGGCCGATCACCCTCTCAGGTCGGCTATGTATCGTTGCCTTGGTG  248

Query  1201   AGCCGTTACCTCACCAACTAGCTAATACAACGCAGGTCCATCTCATAGTGAAGCAATTGC  1260
              |||||||||||||||||||||||||||||||||||||||||||  |||||||||||||||
Sbjct  247    AGCCGTTACCTCACCAACTAGCTAATACAACGCAGGTCCATCTGGTAGTGAAGCAATTGC  188

Query  1261   TCCTTTCAAATATCTACCATGCGGTAAATACTGTTATGCGGTATTAGCTATCGTTTCCAA  1320
              |||||||||| | ||| |||| |     | ||||||||||||||||||||||||||||||
Sbjct  187    TCCTTTCAAACAGCTAACATGTGTCGGCTGCTGTTATGCGGTATTAGCTATCGTTTCCAA  128

Query  1321   TAGTTATCCCCCGCTATGAGGCAGGTTACCTACGCGTTACTCACCCGTTCGCAACTCATC  1380
              ||||||||||||||||  ||||||||||||||||||||||||||||||||||||||||||
Sbjct  127    TAGTTATCCCCCGCTACCAGGCAGGTTACCTACGCGTTACTCACCCGTTCGCAACTCATC  68

Query  1381   CGTCTAG-TGCAAGCACCAGACTTCAGCGTTCTACTTGCATGTATTAGGCACGCCGCCAG  1439
              || || | |||||||||||  |||||||||||||||||||||||||||||||||||||||
Sbjct  67     CG-CTCGGTGCAAGCACCAAGCTTCAGCGTTCTACTTGCATGTATTAGGCACGCCGCCAG  9

Query  1440   CGTTCGTC  1447
              ||||||||
Sbjct  8      CGTTCGTC  1
```

Streptococcus plurextorum strain 1956-02 16S ribosomal RNA, partial sequence

Sequence ID: **NR_042649.1** Length: 1451 Number of Matches: 1

Range 1: 1 to 1451

Score	Expect	Identities	Gaps	Strand	Frame
2398 bits(1298)	0.0()	1406/1457(96%)	11/1457(0%)	Plus/Minus	

```
Query  4      CGGCTGGCTCCCAAAAGGTTACCTCACCGACTTCGGGTGTTACAAACTCTCGTGGTGTGA  63
              ||||||||| ||  | ||||||||||||||||||||||||||||||||||||||||||||
Sbjct  1451   CGGCTGGCT-CCTTACGGTTACCTCACCGACTTCGGGTGTTACAAACTCTCGTGGTGTGA  1393

Query  64     CGGGCGGTGTGTACAAGGCCCGGGAACGTATTCACCGCGGCGTGCTGATCCGCGATTACT  123
              ||||||||||||||||||||||||||||||||||||||||||||||||||||||||||||
Sbjct  1392   CGGGCGGTGTGTACAAGGCCCGGGAACGTATTCACCGCGGCGTGCTGATCCGCGATTACT  1333

Query  124    AGCGATTCCGACTTCATGTAGGCGAGTTGCAGCCTACAATCCGAACTGAGACTGGCTTTA  183
              ||||||||||||||||||||||||||||||||||||||||||||||||||||||||||||
Sbjct  1332   AGCGATTCCGACTTCATGTAGGCGAGTTGCAGCCTACAATCCGAACTGAGACTGGCTTTA  1273

Query  184    AGAGATTAGCTTGCCGTCACCGACTTGCGACTCGTTGTACCAGCCATTGTAGCACGTGTG  243
              ||||||| ||||||||||||||| ||||||||||||||||||||||||||||||||||||
Sbjct  1272   AGAGATTTGCTTGCCGTCACCGAGTTGCGACTCGTTGTACCAGCCATTGTAGCACGTGTG  1213

Query  244    TAGCCCAGGTCATAAGGGGCATGATGATTTGACGTCATCCCCACCTTCCTCCGGTTTATT  303
              ||||||||||||||||||||||||||||||||||||||||||||||||||||||||||||
Sbjct  1212   TAGCCCAGGTCATAAGGGGCATGATGATTTGACGTCATCCCCACCTTCCTCCGGTTTATT  1153

Query  304    ACCGGCAGTCTCGCTAGAGTGCCCAACTGAATGATGGCAACTAACAATAGGGGTTGCGCT  363
              ||||||||||||||||||||||||||||||||||||||||||||||||||||||||||||
Sbjct  1152   ACCGGCAGTCTCGCTAGAGTGCCCAACTGAATGATGGCAACTAACAATAGGGGTTGCGCT  1093

Query  364    CGTTGCGGGACTTAACCCAACATCTCACGACACGAGCTGACGACAACCATGCACCACCTG  423
              ||||||||||||||||||||||||||||||||||||||||||||||||||||||||||||
Sbjct  1092   CGTTGCGGGACTTAACCCAACATCTCACGACACGAGCTGACGACAACCATGCACCACCTG  1033

Query  424    TCACCGATGCTCCGAAGAGAAACCCTATCTCTAGGGCGGTCATCGGGATGTCAAGACCTG  483
              |||||  ||||||||||||||| |||||||||||| ||||||  ||||||||||||||||
Sbjct  1032   TCACCAGTGCTCCGAAGAGAAATCCTATCTCTAGGACGGTCACTGGGATGTCAAGACCTG  973

Query  484    GTAAGGTTCTTCGCGTTGCTTCGAATTAAACCACATGCTCCACCGCTTGTGCGGGCCCCC  543
              ||||||||||||||||||||||||||||||||||||||||||||||||||||||||||||
Sbjct  972    GTAAGGTTCTTCGCGTTGCTTCGAATTAAACCACATGCTCCACCGCTTGTGCGGGCCCCC  913

Query  544    GTCAATTCCTTTGAGTTTCAACCTTGCGGTCGTACTCCCCAGGCGGAGTGCTTAATGCGT  603
              ||||||||||||||||||||||||||||||||||||||||||||||||||||||||||||
Sbjct  912    GTCAATTCCTTTGAGTTTCAACCTTGCGGTCGTACTCCCCAGGCGGAGTGCTTAATGCGT  853

Query  604    TAGCTGCGGCACTGAGTCCCGGAAAGGACCCAACACCTAGCACTCATCGTTTACGGCGTG  663
              ||||||||||||| || |||||||||| || |||||||||||||||||||||||||||||
Sbjct  852    TAGCTGCGGCACTAAGCCCCGGAAAGGGCCTAACACCTAGCACTCATCGTTTACGGCGTG  793

Query  664    GACTACCAGGGTATCTAATCCTGTTCGCTCCCCACGCTTTCGAGCCTCAGCGTCAGTTAC  723
```

```
Sbjct  792   GACTACCAGGGTATCTAATCCTGTTCGCTACCCACGCTTTCGAGCCTCAGCGTCAGTTAC  733

Query  724   AGACCAGAGAGCCGCTTTCGCCACCGGTGTTCCTCCATATATCTACGCATTTCACCGCTA  783
Sbjct  732   AGACCAGAGAGCCGCTTTCGCCACCGGTGTTCCTCCATATATCTACGCATTTCACCGCTA  673

Query  784   CACATGGAATTCCACTCTCCCCTTCTGCACTCAAGTTTGACAGTTTCCAAAGCGTACTAT  843
Sbjct  672   CACATGGAATTCCACTCTCCCCTTCTGCACTCAAGTTCTACAGTTTCCAAAGCGTACATT  613

Query  844   GGTTAAGCCACAGCCTTTTACTTCAGACTTATCAAACCGCCTGCGCTCGCTTTACGCCCA  903
Sbjct  612   GGTTGAGCCAATGCCTTTGACTTCAGACTTATATAACCGCCTGCGCTCGCTTTACGCCCA  553

Query  904   ATAAATCCGGACAACGCTCGGGACCTACGTATTACCGCGGCTGCTGGCACGTAGTTAGCC  963
Sbjct  552   ATAAATCCGGACAACGCTCGGGACCTACGTATTACCGCGGCTGCTGGCACGTAGTTAGCC  493

Query  964   GTCCCTTTCTGGTAAGATACCGTCA--A-GTGAGAAACTTTCCACTCTT-CTCACAGTTC  1019
Sbjct  492   GTCCCTTTCTGGTAAGATACCGTCATTATGTG-G--ACTTTCCACTCCCACACACA-TTC  437

Query  1020  TTCTCTTACAACAGAGCTTTACGATCCGAAAACCTTCTTCACTCACGCGGCGTTGCTCGG  1079
Sbjct  436   TTCTCTTACAACAGAGCTTTACGATCCGAAAACCTTCTTCACTCACGCGGCGTTGCTCGG  377

Query  1080  TCAGGGTTGCCCCCATTGCCGAAGATTCCCTACTGCTGCCTCCCGTAGGAGTCTGGGCCG  1139
Sbjct  376   TCAGGGTTGCCCCCATTGCCGAAGATTCCCTACTGCTGCCTCCCGTAGGAGTCTGGGCCG  317

Query  1140  TGTCTCAGTCCCAGTGTGGCCGATCACCCTCTCAGGTCGGCTATGTATCGAAGCCTTGGT  1199
Sbjct  316   TGTCTCAGTCCCAGTGTGGCCGATCACCCTCTCAGGTCGGCTATGTATCGTCGCCTTGGT  257

Query  1200  GAGCCGTTACCTCACCAACTAGCTAATACAACGCAGGTCCATCTCATAGTGAAGCAATTG  1259
Sbjct  256   GAGCTGTTACCTCACCAACTAGCTAATACAACGCAGGTCCATCTCATAGTGAAGCAATTG  197

Query  1260  CTCCTTTCAAATATCTACCATGCGGTAAATACTGTTATGCGGTATTAGCTATCGTTTCCA  1319
Sbjct  196   CTTCTTTCAAGTATCTACCATGCGGTAAATACTGTTATGCGGTATTAGCTATCGTTTCCA  137

Query  1320  ATAGTTATCCCCCGCTATGAGGCAGGTTACCTACGCGTTACTCACCCGTTCGCAACTCAT  1379
Sbjct  136   ATAGTTATCCCCCGCTATGAGCCAGGTTACCTACGCGTTACTCACCCGTTCGCAACTCCT  77

Query  1380  C-CGTCTAGTGCAAGCACCAGACTTCAGCGTTCTACTTGCATGTATTAGGCACGCCGCCA  1438
Sbjct  76    CACTTC-AGTGCAAGCACCAAAGCTCAGCGTTCTACTTGCATGTATTAGGCACGCCGCCA  18

Query  1439  GCGTTCGTCCTGAGCCA  1455
Sbjct  17    GCGTTCGTCCTGAGCCA  1
```

Streptococcus saliviloxodontae strain NUM 6306 16S ribosomal RNA, partial sequence
Sequence ID: **NR_126178.1** Length: 1504 Number of Matches: 1
Range 1: 1 to 1447

Score	Expect	Identities	Gaps	Strand	Frame
2396 bits(1297)	0.0()	1397/1447(97%)	0/1447(0%)	Plus/Minus	

```
Query  1     AGGCGGCTGGCTCCCAAAAGGTTACCTCACCGACTTCGGGTGTTACAAACTCTCGTGGTG  60
Sbjct  1447  AGGCGGCTGGCTCCTAAAAGGTTACCTCACCGACTTCGGGTGTTACAAACTCTCGTGGTG  1388

Query  61    TGACGGGCGGTGTGTACAAGGCCCGGGAACGTATTCACCGCGGCGTGCTGATCCGCGATT  120
Sbjct  1387  TGACGGGCGGTGTGTACAAGGCCCGGGAACGTATTCACCGCGGCGTGCTGATCCGCGATT  1328

Query  121   ACTAGCGATTCCGACTTCATGTAGGCGAGTTGCAGCCTACAATCCGAACTGAGACTGGCT  180
Sbjct  1327  ACTAGCGATTCCGACTTCATGTAGGCGAGTTGCAGCCTACAATCCGAACTGAGATTGGCT  1268

Query  181   TTAAGAGATTAGCTTGCCGTCACCGACTTGCGACTCGTTGTACCAGCCATTGTAGCACGT  240
Sbjct  1267  TTAAGAGATTAGCTTGCCGTCACCGGCTTGCGACTCGTTGTACCAACCATTGTAGCACGT  1208

Query  241   GTGTAGCCCAGGTCATAAGGGGCATGATGATTTGACGTCATCCCCACCTTCCTCCGGTTT  300
Sbjct  1207  GTGTAGCCCAGGTCATAAGGGGCATGATGATTTGACGTCATCCCCACCTTCCTCCGGTTT  1148

Query  301   ATTACCGGCAGTCTCGCTAGAGTGCCCAACTGAATGATGGCAACTAACAATAGGGGTTGC  360
Sbjct  1147  ATTACCGGCAGTCTCGCTAGAGTGCCCAACTTAATGATGGCAACTAACAATAGGGGTTGC  1088

Query  361   GCTCGTTGCGGGACTTAACCCAACATCTCACGACACGAGCTGACGACAACCATGCACCAC  420
Sbjct  1087  GCTCGTTGCGGGACTTAACCCAACATCTCACGACACGAGCTGACGACAACCATGCACCAC  1028

Query  421   CTGTCACCGATGCTCCGAAGAGAAACCCTATCTCTAGGGCGGTCATCGGGATGTCAAGAC  480
Sbjct  1027  CTGTCACCGATGTTCCGAAGAAACTTCCTATCTCTAGGAATAGCATCGGGATGTCAAGAC  968

Query  481   CTGGTAAGGTTCTTCGCGTTGCTTCGAATTAAACCACATGCTCCACCGCTTGTGCGGGCC  540
Sbjct  967   CTGGTAAGGTTCTTCGCGTTGCTTCGAATTAAACCACATGCTCCACCGCTTGTGCGGGCC  908

Query  541   CCCGTCAATTCCTTTGAGTTTCAACCTTGCGGTCGTACTCCCCAGGCGGAGTGCTTAATG  600
Sbjct  907   CCCGTCAATTCCTTTGAGTTTCAACCTTGCGGTCGTACTCCCCAGGCGGAGTGCTTAATG  848

Query  601   CGTTAGCTGCGGCACTGAGTCCCGGAAAGGACCCAACACCTAGCACTCATCGTTTACGGC  660
Sbjct  847   CGTTAGCTGCGGCACTGAGTCCCGGAAAGGACCCAACACCTAGCACTCATCGTTTACGGC  788

Query  661   GTGGACTACCAGGGTATCTAATCCTGTTCGCTCCCCACGCTTTCGAGCCTCAGCGTCAGT  720
Sbjct  787   GTGGACTACCAGGGTATCTAATCCTGTTCGCTCCCCACGCTTTCGAGCCTCAGCGTCAGT  728

Query  721   TACAGACCAGAGAGCCGCTTTCGCCACCGGTGTTCCTCCATATATCTACGCATTTCACCG  780
Sbjct  727   TACAGACCAGAGAGCCGCTTTCGCCACCGGTGTTCCTCCATATATCTACGCATTTCACCG  668

Query  781   CTACACATGGAATTCCACTCTCCCCTTCTGCACTCAAGTTTGACAGTTTCCAAAGCGTAC  840
Sbjct  667   CTACACATGGAATTCCACTCTCCCCTTCTGCACTCAAGTTTGACAGTTTCCAAAGCGAAC  608

Query  841   TATGGTTAAGCCACAGCCTTTTACTTCAGACTTATCAAACCGCCTGCGCTCGCTTTACGC  900
Sbjct  607   AATGGTTGAGCCACTGCCTTTAACTTCAGACTTATCAAACCGCCTGCGCTCGCTTTACGC  548

Query  901   CCAATAAATCCGGACAACGCTCGGGACCTACGTATTACCGCGGCTGCTGGCACGTAGTTA  960
```

```
Sbjct  547   CCAATAAATCCGGACAACGCTCGGGACCTACGTATTACCGCGGCTGCTGGCACGTAGTTA  488

Query  961   GCCGTCCCTTTCTGGTAAGATACCGTCAAGTGAGAAACTTTCCACTCTTCTCACAGTTCT  1020
             |||||||||||||||| || ||||||||  |||  |||||||||||||  |||  |||||
Sbjct  487   GCCGTCCCTTTCTGGTTAGTTACCGTCACTTGATGAACTTTCCACTCTCATCAACGTTCT  428

Query  1021  TCTCTTACAACAGAGCTTTACGATCCGAAAACCTTCTTCACTCACGCGGCGTTGCTCGGT  1080
             ||||| ||||||||||||||||||||||||||||||||||||||||||||||||||||||
Sbjct  427   TCTCTAACAACAGAGCTTTACGATCCGAAAACCTTCTTCACTCACGCGGCGTTGCTCGGT  368

Query  1081  CAGGGTTGCCCCCATTGCCGAAGATTCCCTACTGCTGCCTCCCGTAGGAGTCTGGGCCGT  1140
             ||||||||||||||||||||||||||||||||||||||||||||||||||||||||||||
Sbjct  367   CAGGGTTGCCCCCATTGCCGAAGATTCCCTACTGCTGCCTCCCGTAGGAGTCTGGGCCGT  308

Query  1141  GTCTCAGTCCCAGTGTGGCCGATCACCCTCTCAGGTCGGCTATGTATCGAAGCCTTGGTG  1200
             |||||||||||||||||||||||||||||||||||||||||||||||||  |||||||||
Sbjct  307   GTCTCAGTCCCAGTGTGGCCGATCACCCTCTCAGGTCGGCTATGTATCGTCGCCTTGGTG  248

Query  1201  AGCCGTTACCTCACCAACTAGCTAATACAACGCAGGTCCATCTCATAGTGAAGCAATTGC  1260
             |||||||||||||||||||||||||||||||||||||||||||  ||||| |||||||||
Sbjct  247   AGCCGTTACCTCACCAACTAGCTAATACAACGCAGGTCCATCTTGTAGTGGAGCAATTGC  188

Query  1261  TCCTTTCAAATATCTACCATGCGGTAAATACTGTTATGCGGTATTAGCTATCGTTTCCAA  1320
              |||||||| |   || |||| | ||   ||| |||||||||||||||||||||||||||
Sbjct  187   CCCTTTCAAGTTAGTAACATGTGTTACTAACTATTATGCGGTATTAGCTATCGTTTCCAA  128

Query  1321  TAGTTATCCCCCGCTATGAGGCAGGTTACCTACGCGTTACTCACCCGTTCGCAACTCATC  1380
             ||||||||||||||||  ||||||||||||||||||||||||||||||||||||||||||
Sbjct  127   TAGTTATCCCCCGCTACAAGGCAGGTTACCTACGCGTTACTCACCCGTTCGCAACTCATC  68

Query  1381  CGTCTAGTGCAAGCACCAGACTTCAGCGTTCTACTTGCATGTATTAGGCACGCCGCCAGC  1440
             ||||||||||||||||||||||||||||||||||||||||||||||||||||||||||||
Sbjct  67    CGTCTAGTGCAAGCACCAGACTTCAGCGTTCTACTTGCATGTATTAGGCACGCCGCCAGC  8

Query  1441  GTTCGTC  1447
             |||||||
Sbjct  7     GTTCGTC  1
```

Taxonomy

Reports

- **Lineage**

Organism	Blast Name	Score	Number of Hits	Description
Streptococcus	firmicutes		100	
.Streptococcus suis	firmicutes	2684	3	**Streptococcus suis hits**
.Streptococcus parasuis	firmicutes	2501	2	**Streptococcus parasuis hits**
.Streptococcus oriscaviae	firmicutes	2435	2	**Streptococcus oriscaviae hits**
.Streptococcus respiraculi	firmicutes	2427	1	**Streptococcus respiraculi hits**
.Streptococcus gallinaceus	firmicutes	2401	1	**Streptococcus gallinaceus hits**
.Streptococcus plurextorum	firmicutes	2398	1	**Streptococcus plurextorum hits**
.Streptococcus saliviloxodontae	firmicutes	2396	1	**Streptococcus saliviloxodontae hits**
.Streptococcus himalayensis	firmicutes	2386	1	**Streptococcus himalayensis hits**
.Streptococcus koreensis	firmicutes	2379	1	**Streptococcus koreensis hits**
.Streptococcus cristatus AS 1.3089	firmicutes	2366	1	**Streptococcus cristatus AS 1.3089 hits**
.Streptococcus parasanguinis ATCC 15912	firmicutes	2366	3	**Streptococcus parasanguinis ATCC 15912 hits**
.Streptococcus marmotae	firmicutes	2361	1	**Streptococcus marmotae hits**
.Streptococcus porcorum	firmicutes	2357	1	**Streptococcus porcorum hits**
.Streptococcus cristatus ATCC 51100	firmicutes	2353	2	**Streptococcus cristatus ATCC 51100 hits**
.Streptococcus sinensis	firmicutes	2348	1	**Streptococcus sinensis hits**
.Streptococcus sanguinis SK1 = NCTC 7863	firmicutes	2346	2	**Streptococcus sanguinis SK1 = NCTC 7863 hits**
.Streptococcus australis	firmicutes	2346	1	**Streptococcus australis hits**
.Streptococcus hyointestinalis	firmicutes	2342	1	**Streptococcus hyointestinalis hits**
.Streptococcus vestibularis ATCC 49124	firmicutes	2342	1	**Streptococcus vestibularis ATCC 49124 hits**
.Streptococcus salivarius	firmicutes	2342	1	**Streptococcus salivarius hits**
.Streptococcus penaeicida	firmicutes	2340	1	**Streptococcus penaeicida hits**
.Streptococcus rubneri	firmicutes	2340	1	**Streptococcus rubneri hits**
.Streptococcus urinalis	firmicutes	2340	2	**Streptococcus urinalis hits**
.Streptococcus lutetiensis	firmicutes	2338	3	**Streptococcus lutetiensis hits**
.Streptococcus loxodontisalivarius	firmicutes	2337	1	**Streptococcus loxodontisalivarius hits**
.Streptococcus ilei	firmicutes	2335	1	**Streptococcus ilei hits**
.Streptococcus gordonii	firmicutes	2333	2	**Streptococcus gordonii hits**
.Streptococcus equinus	firmicutes	2333	2	**Streptococcus equinus hits**
.Streptococcus vicugnae	firmicutes	2331	1	**Streptococcus vicugnae hits**
.Streptococcus thermophilus	firmicutes	2331	2	**Streptococcus thermophilus hits**
.Streptococcus uberis	firmicutes	2329	1	**Streptococcus uberis hits**
.Streptococcus ruminantium	firmicutes	2327	1	**Streptococcus ruminantium hits**
.Streptococcus sanguinis	firmicutes	2327	1	**Streptococcus sanguinis hits**
.Streptococcus hillyeri	firmicutes	2324	1	**Streptococcus hillyeri hits**
.Streptococcus agalactiae ATCC 13813	firmicutes	2324	3	**Streptococcus agalactiae ATCC 13813 hits**
.Streptococcus oralis ATCC 35037	firmicutes	2318	2	**Streptococcus oralis ATCC 35037 hits**
.Streptococcus macedonicus	firmicutes	2318	1	**Streptococcus macedonicus hits**

.Streptococcus cristatus	firmicutes	2318	1	Streptococcus cristatus hits
.Streptococcus oralis subsp. tigurinus AZ_3a	firmicutes	2316	1	Streptococcus oralis subsp. tigurinus AZ_3a hits
.Streptococcus oralis subsp. dentisani	firmicutes	2314	1	Streptococcus oralis subsp. dentisani hits
.Streptococcus moroccensis	firmicutes	2314	1	Streptococcus moroccensis hits
.Streptococcus acidominimus	firmicutes	2311	1	Streptococcus acidominimus hits
.Streptococcus oralis	firmicutes	2311	1	Streptococcus oralis hits
.Streptococcus azizii	firmicutes	2309	1	Streptococcus azizii hits
.Streptococcus tangierensis	firmicutes	2307	1	Streptococcus tangierensis hits
.Streptococcus porcinus	firmicutes	2307	1	Streptococcus porcinus hits
.Streptococcus parauberis	firmicutes	2307	1	Streptococcus parauberis hits
.Streptococcus lactarius	firmicutes	2307	1	Streptococcus lactarius hits
.Streptococcus mitis	firmicutes	2302	3	Streptococcus mitis hits
.Streptococcus downii	firmicutes	2300	1	Streptococcus downii hits
.Streptococcus iniae	firmicutes	2300	2	Streptococcus iniae hits
.Streptococcus vulneris	firmicutes	2298	1	Streptococcus vulneris hits
.Streptococcus alactolyticus	firmicutes	2298	1	Streptococcus alactolyticus hits
.Streptococcus toyakuensis	firmicutes	2290	1	Streptococcus toyakuensis hits
.Streptococcus ictaluri 707-05	firmicutes	2290	1	Streptococcus ictaluri 707-05 hits
.Streptococcus panodentis	firmicutes	2289	1	Streptococcus panodentis hits
.Streptococcus cameli	firmicutes	2289	1	Streptococcus cameli hits
.Streptococcus infantis ATCC 700779	firmicutes	2287	1	Streptococcus infantis ATCC 700779 hits
.Streptococcus pasteurianus	firmicutes	2287	1	Streptococcus pasteurianus hits
.Streptococcus timonensis	firmicutes	2285	1	Streptococcus timonensis hits
.Streptococcus cunicolipharyngis	firmicutes	2285	1	Streptococcus cunicolipharyngis hits
.Streptococcus troglodytidis	firmicutes	2283	1	Streptococcus troglodytidis hits
.Streptococcus cuniculi	firmicutes	2281	1	Streptococcus cuniculi hits
.Streptococcus minor	firmicutes	2281	1	Streptococcus minor hits
.Streptococcus varani	firmicutes	2279	1	Streptococcus varani hits
.Streptococcus dysgalactiae subsp. equisimilis	firmicutes	2279	1	Streptococcus dysgalactiae subsp. equisimilis hits
.Streptococcus xiaochunlingii	firmicutes	2278	1	Streptococcus xiaochunlingii hits
.Streptococcus porci	firmicutes	2278	1	Streptococcus porci hits
.Streptococcus constellatus	firmicutes	2276	1	Streptococcus constellatus hits
.Streptococcus pseudoporcinus LQ 940-04	firmicutes	2276	1	Streptococcus pseudoporcinus LQ 940-04 hits
.Streptococcus pyogenes	firmicutes	2274	1	Streptococcus pyogenes hits
.Streptococcus intermedius	firmicutes	2270	1	Streptococcus intermedius hits
.Streptococcus gwangjuense	firmicutes	2268	1	Streptococcus gwangjuense hits
.Streptococcus oricebi	firmicutes	2263	1	Streptococcus oricebi hits
.Streptococcus chosunense	firmicutes	2254	1	Streptococcus chosunense hits
.Streptococcus anginosus SK52 = DSM 20563	firmicutes	2254	1	Streptococcus anginosus SK52 = DSM 20563 hits
.Streptococcus constellatus subsp. pharyngis	firmicutes	2252	1	Streptococcus constellatus subsp. pharyngis hits
.Streptococcus oriloxodontae	firmicutes	2246	1	Streptococcus oriloxodontae hits
.Streptococcus pneumoniae	firmicutes	2246	1	Streptococcus pneumoniae hits
.Streptococcus caledonicus	firmicutes	2242	1	Streptococcus caledonicus hits

- **Organism**

Description	Score	E value	Accession
Streptococcus suis [firmicutes]			
**Streptococcus suis strain S735 16S ribosomal RNA, partial sequence**	2684	0.0	**NR_036918**
**Streptococcus suis strain ATCC 43765 16S ribosomal RNA, partial sequence**	2667	0.0	**NR_115737**
**Streptococcus suis strain ATCC 43765 16S ribosomal RNA, partial sequence**	2462	0.0	**NR_117504**
Streptococcus parasuis [firmicutes]			
**Streptococcus parasuis strain SUT-286 16S ribosomal RNA, partial sequence**	2501	0.0	**NR_179215**
**Streptococcus parasuis strain SUT-286 16S ribosomal RNA, partial sequence**	2388	0.0	**NR_178262**
Streptococcus oriscaviae [firmicutes]			
**Streptococcus oriscaviae strain HKU75 16S ribosomal RNA, complete sequence**	2435	0.0	**NR_181935**
**Streptococcus oriscaviae strain HKU75 16S ribosomal RNA, partial sequence**	2435	0.0	**NR_181481**
Streptococcus respiraculi [firmicutes]			
**Streptococcus respiraculi strain HTS25 16S ribosomal RNA, partial sequence**	2427	0.0	**NR_179720**
Streptococcus gallinaceus [firmicutes]			
**Streptococcus gallinaceus strain CCUG 42692 16S ribosomal RNA, partial sequence**	2401	0.0	**NR_025453**
Streptococcus plurextorum [firmicutes]			
**Streptococcus plurextorum strain 1956-02 16S ribosomal RNA, partial sequence**	2398	0.0	**NR_042649**
Streptococcus saliviloxodontae [firmicutes]			

Description	Score	E value	Accession
**Streptococcus saliviloxodontae strain NUM 6306 16S ribosomal RNA, partial sequence**	2396	0.0	NR_126178
Streptococcus himalayensis [firmicutes]			
**Streptococcus himalayensis strain HTS2 16S ribosomal RNA, partial sequence**	2386	0.0	NR_156072
Streptococcus koreensis [firmicutes]			
**Streptococcus koreensis strain KCOM 2890 16S ribosomal RNA, partial sequence**	2379	0.0	NR_165737
Streptococcus cristatus AS 1.3089 [firmicutes]			
**Streptococcus cristatus AS 1.3089 16S ribosomal RNA, partial sequence**	2366	0.0	NR_103943
Streptococcus parasanguinis ATCC 15912 [firmicutes]			
**Streptococcus parasanguinis ATCC 15912 16S ribosomal RNA, partial sequence**	2366	0.0	NR_024842
**Streptococcus parasanguinis ATCC 15912 16S ribosomal RNA, partial sequence**	2351	0.0	NR_115241
**Streptococcus parasanguinis ATCC 15912 16S ribosomal RNA, partial sequence**	2333	0.0	NR_115735
Streptococcus marmotae [firmicutes]			
**Streptococcus marmotae strain HTS5 16S ribosomal RNA, partial sequence**	2361	0.0	NR_152678
Streptococcus porcorum [firmicutes]			
**Streptococcus porcorum strain 682/03 16S ribosomal RNA, partial sequence**	2357	0.0	NR_108477
Streptococcus cristatus ATCC 51100 [firmicutes]			
**Streptococcus cristatus ATCC 51100 16S ribosomal RNA, partial sequence**	2353	0.0	NR_042771
**Streptococcus cristatus ATCC 51100 16S ribosomal RNA, partial sequence**	2327	0.0	NR_115274
Streptococcus sinensis [firmicutes]			
**Streptococcus sinensis strain HKU4 16S ribosomal RNA, partial sequence**	2348	0.0	NR_028833
Streptococcus sanguinis SK1 = NCTC 7863 [firmicutes]			
**Streptococcus sanguinis SK1 = NCTC 7863 16S ribosomal RNA, partial sequence**	2346	0.0	NR_024841
**Streptococcus sanguinis SK1 = NCTC 7863 16S ribosomal RNA, partial sequence**	2248	0.0	NR_115736
Streptococcus australis [firmicutes]			
**Streptococcus australis strain AI-1 16S ribosomal RNA, partial sequence**	2346	0.0	NR_036936
Streptococcus hyointestinalis [firmicutes]			
**Streptococcus hyointestinalis strain ATCC 49169 16S ribosomal RNA, partial sequence**	2342	0.0	NR_041780
Streptococcus vestibularis ATCC 49124 [firmicutes]			
**Streptococcus vestibularis ATCC 49124 16S ribosomal RNA, partial sequence**	2342	0.0	NR_042777
Streptococcus salivarius [firmicutes]			
**Streptococcus salivarius strain ATCC 7073 16S ribosomal RNA, partial sequence**	2342	0.0	NR_042776
Streptococcus penaeicida [firmicutes]			
**Streptococcus penaeicida strain CAIM 1838 16S ribosomal RNA, partial sequence**	2340	0.0	NR_178901
Streptococcus rubneri [firmicutes]			
**Streptococcus rubneri strain LMG 27207 16S ribosomal RNA, partial sequence**	2340	0.0	NR_109720
Streptococcus urinalis [firmicutes]			
**Streptococcus urinalis strain 2285-97 16S ribosomal RNA gene, partial sequence**	2340	0.0	NR_037101
**Streptococcus urinalis strain 2285-97 16S ribosomal RNA, partial sequence**	2337	0.0	NR_115738
Streptococcus lutetiensis [firmicutes]			
**Streptococcus lutetiensis strain HDP90246 16S ribosomal RNA, partial sequence**	2338	0.0	NR_037096
**Streptococcus lutetiensis strain CIP 106849 16S ribosomal RNA, partial sequence**	2331	0.0	NR_115719
**Streptococcus lutetiensis strain NEM 782 16S ribosomal RNA, partial sequence**	2274	0.0	NR_042051
Streptococcus loxodontisalivarius [firmicutes]			
**Streptococcus loxodontisalivarius strain NUM 6304 16S ribosomal RNA, partial sequence**	2337	0.0	NR_126177
Streptococcus ilei [firmicutes]			
**Streptococcus ilei strain I-G2 16S ribosomal RNA, partial sequence**	2335	0.0	NR_178515
Streptococcus gordonii [firmicutes]			
**Streptococcus gordonii strain SK3 16S ribosomal RNA, partial sequence**	2333	0.0	NR_028666
**Streptococcus gordonii strain ATCC 10558 16S ribosomal RNA, partial sequence**	2314	0.0	NR_115242
Streptococcus equinus [firmicutes]			
**Streptococcus equinus strain NBRC 12553 16S ribosomal RNA, partial sequence**	2333	0.0	NR_113594
**Streptococcus equinus strain NCDO 1037 16S ribosomal RNA, partial sequence**	2296	0.0	NR_114642
Streptococcus vicugnae [firmicutes]			
**Streptococcus vicugnae strain SL1232 16S ribosomal RNA, partial sequence**	2331	0.0	NR_181987
Streptococcus thermophilus [firmicutes]			
**Streptococcus thermophilus strain ATCC 19258 16S ribosomal RNA, partial sequence**	2331	0.0	NR_042778
**Streptococcus thermophilus strain DSM 20617 16S ribosomal RNA, partial sequence**	2289	0.0	NR_118998
Streptococcus uberis [firmicutes]			
**Streptococcus uberis strain JCM 5709 16S ribosomal RNA, partial sequence**	2329	0.0	NR_040820
Streptococcus ruminantium [firmicutes]			
**Streptococcus ruminantium strain GUT-187 16S ribosomal RNA, partial sequence**	2327	0.0	NR_158064
Streptococcus sanguinis [firmicutes]			

Description	Score	E value	Accession
Streptococcus sanguinis strain JCM 5708 16S ribosomal RNA, partial sequence	2327	0.0	NR_113260
Streptococcus hillyeri [firmicutes]			
Streptococcus hillyeri strain 28462 16S ribosomal RNA, partial sequence	2324	0.0	NR_180151
Streptococcus agalactiae ATCC 13813 [firmicutes]			
Streptococcus agalactiae ATCC 13813 strain JCM 5671 16S ribosomal RNA, partial sequence	2324	0.0	NR_040821
Streptococcus agalactiae ATCC 13813 strain JCM 5671 16S ribosomal RNA, partial sequence	2324	0.0	NR_113262
Streptococcus agalactiae ATCC 13813 16S ribosomal RNA, partial sequence	2309	0.0	NR_115728
Streptococcus oralis ATCC 35037 [firmicutes]			
Streptococcus oralis ATCC 35037 16S ribosomal RNA, partial sequence	2318	0.0	NR_114413
Streptococcus oralis ATCC 35037 16S ribosomal RNA, partial sequence	2318	0.0	NR_042927
Streptococcus macedonicus [firmicutes]			
Streptococcus macedonicus strain LAB617 16S ribosomal RNA, partial sequence	2318	0.0	NR_037002
Streptococcus cristatus [firmicutes]			
Streptococcus cristatus strain 2-4 16S ribosomal RNA, partial sequence	2318	0.0	NR_029052
Streptococcus oralis subsp. tigurinus AZ_3a [firmicutes]			
Streptococcus oralis subsp. tigurinus AZ_3a 16S ribosomal RNA, partial sequence	2316	0.0	NR_118234
Streptococcus oralis subsp. dentisani [firmicutes]			
Streptococcus oralis subsp. dentisani strain 7747 16S ribosomal RNA, partial sequence	2314	0.0	NR_117719
Streptococcus moroccensis [firmicutes]			
Streptococcus moroccensis strain CCMM B831 16S ribosomal RNA, partial sequence	2314	0.0	NR_134191
Streptococcus acidominimus [firmicutes]			
Streptococcus acidominimus strain LMG 17755 16S ribosomal RNA, partial sequence	2311	0.0	NR_104972
Streptococcus oralis [firmicutes]			
Streptococcus oralis strain CCUG 24891 16S ribosomal RNA, partial sequence	2311	0.0	NR_115734
Streptococcus azizii [firmicutes]			
Streptococcus azizii strain 12-5202 16S ribosomal RNA, partial sequence	2309	0.0	NR_159228
Streptococcus tangierensis [firmicutes]			
Streptococcus tangierensis strain CCMM B832 16S ribosomal RNA, partial sequence	2307	0.0	NR_134818
Streptococcus porcinus [firmicutes]			
Streptococcus porcinus strain 176 16S ribosomal RNA, partial sequence	2307	0.0	NR_024634
Streptococcus parauberis [firmicutes]			
Streptococcus parauberis strain DSM 6631 16S ribosomal RNA, partial sequence	2307	0.0	NR_043001
Streptococcus lactarius [firmicutes]			
Streptococcus lactarius strain MV1 16S ribosomal RNA, partial sequence	2307	0.0	NR_117425
Streptococcus mitis [firmicutes]			
Streptococcus mitis strain NS51 16S ribosomal RNA, partial sequence	2302	0.0	NR_028664
Streptococcus mitis strain ATCC 49456 16S ribosomal RNA, partial sequence	2278	0.0	NR_115240
Streptococcus mitis strain ATCC 49456 16S ribosomal RNA, partial sequence	2270	0.0	NR_115732
Streptococcus downii [firmicutes]			
Streptococcus downii 16S ribosomal RNA, partial sequence	2300	0.0	NR_175455
Streptococcus iniae [firmicutes]			
Streptococcus iniae strain ATCC 29178 16S ribosomal RNA, partial sequence	2300	0.0	NR_025148
Streptococcus iniae strain ATCC 29178 16S ribosomal RNA, partial sequence	2244	0.0	NR_115731
Streptococcus vulneris [firmicutes]			
Streptococcus vulneris strain DM3B3 16S ribosomal RNA, complete sequence	2298	0.0	NR_179383
Streptococcus alactolyticus [firmicutes]			
Streptococcus alactolyticus strain ATCC 43077 16S ribosomal RNA, partial sequence	2298	0.0	NR_041781
Streptococcus toyakuensis [firmicutes]			
Streptococcus toyakuensis strain TP1632 16S ribosomal RNA, partial sequence	2290	0.0	NR_179385
Streptococcus ictaluri 707-05 [firmicutes]			
Streptococcus ictaluri 707-05 16S ribosomal RNA, partial sequence	2290	0.0	NR_115802
Streptococcus panodentis [firmicutes]			
Streptococcus panodentis strain TKU50 16S ribosomal RNA, partial sequence	2289	0.0	NR_145950
Streptococcus cameli [firmicutes]			
Streptococcus cameli strain CCMM B834 16S ribosomal RNA, partial sequence	2289	0.0	NR_134817
Streptococcus infantis ATCC 700779 [firmicutes]			
Streptococcus infantis ATCC 700779 16S ribosomal RNA, partial sequence	2287	0.0	NR_042928
Streptococcus pasteurianus [firmicutes]			
Streptococcus pasteurianus strain CIP 107122 16S ribosomal RNA, partial sequence	2287	0.0	NR_043660
Streptococcus timonensis [firmicutes]			
Streptococcus timonensis strain Marseille-P2915 16S ribosomal RNA, partial sequence	2285	0.0	NR_179540
Streptococcus cuniculipharyngis [firmicutes]			

Description	Score	E value	Accession
**Streptococcus cuniculipharyngis strain DICM10-00796B 16S ribosomal RNA, partial sequence**	2285	0.0	**NR_137219**
Streptococcus troglodytidis [firmicutes]			
**Streptococcus troglodytidis strain M09-11185 16S ribosomal RNA, partial sequence**	2283	0.0	**NR_109371**
Streptococcus cuniculi [firmicutes]			
**Streptococcus cuniculi strain : NED12-00049-6B 16S ribosomal RNA, partial sequence**	2281	0.0	**NR_134190**
Streptococcus minor [firmicutes]			
**Streptococcus minor strain ON59 16S ribosomal RNA, partial sequence**	2281	0.0	**NR_025729**
Streptococcus varani [firmicutes]			
**Streptococcus varani strain FF10 16S ribosomal RNA, partial sequence**	2279	0.0	**NR_179404**
Streptococcus dysgalactiae subsp. equisimilis [firmicutes]			
**Streptococcus dysgalactiae subsp. equisimilis strain CIP 105120 16S ribosomal RNA, partial sequence**	2279	0.0	**NR_043661**
Streptococcus xiaochunlingii [firmicutes]			
**Streptococcus xiaochunlingii strain E24 16S ribosomal RNA, partial sequence**	2278	0.0	**NR_180875**
Streptococcus porci [firmicutes]			
**Streptococcus porci strain 2923-03 16S ribosomal RNA, partial sequence**	2278	0.0	**NR_115087**
Streptococcus constellatus [firmicutes]			
**Streptococcus constellatus strain ATCC 27823 16S ribosomal RNA, partial sequence**	2276	0.0	**NR_041721**
Streptococcus pseudoporcinus LQ 940-04 [firmicutes]			
**Streptococcus pseudoporcinus LQ 940-04 16S ribosomal RNA, partial sequence**	2276	0.0	**NR_043704**
Streptococcus pyogenes [firmicutes]			
**Streptococcus pyogenes strain JCM 5674 16S ribosomal RNA, partial sequence**	2274	0.0	**NR_112088**
Streptococcus intermedius [firmicutes]			
**Streptococcus intermedius strain 1877 16S ribosomal RNA, partial sequence**	2270	0.0	**NR_028736**
Streptococcus gwangjuense [firmicutes]			
**Streptococcus gwangjuense strain ChDC B345 16S ribosomal RNA, partial sequence**	2268	0.0	**NR_165744**
Streptococcus oricebi [firmicutes]			
**Streptococcus oricebi strain M8 16S ribosomal RNA, partial sequence**	2263	0.0	**NR_148589**
Streptococcus chosunense [firmicutes]			
**Streptococcus chosunense strain ChDC B353 16S ribosomal RNA, partial sequence**	2254	0.0	**NR_165741**
Streptococcus anginosus SK52 = DSM 20563 [firmicutes]			
**Streptococcus anginosus SK52 = DSM 20563 16S ribosomal RNA, partial sequence**	2254	0.0	**NR_117426**
Streptococcus constellatus subsp. pharyngis [firmicutes]			
**Streptococcus constellatus subsp. pharyngis strain MM9889a 16S ribosomal RNA, partial sequence**	2252	0.0	**NR_042833**
Streptococcus oriloxodontae [firmicutes]			
**Streptococcus oriloxodontae strain NUM 2101 16S ribosomal RNA, partial sequence**	2246	0.0	**NR_178254**
Streptococcus pneumoniae [firmicutes]			
**Streptococcus pneumoniae strain ATCC 33400 16S ribosomal RNA, partial sequence**	2246	0.0	**NR_028665**
Streptococcus caledonicus [firmicutes]			
**Streptococcus caledonicus strain S784/96/1 16S ribosomal RNA, partial sequence**	2242	0.0	**NR_180776**

- ## Taxonomy

Taxonomy	Number of hits	Number of Organisms	Description
Streptococcus	100	80	
. Streptococcus suis	3	1	**Streptococcus suis hits**
. Streptococcus parasuis	2	1	**Streptococcus parasuis hits**
. Streptococcus oriscaviae	2	1	**Streptococcus oriscaviae hits**
. Streptococcus respiraculi	1	1	**Streptococcus respiraculi hits**
. Streptococcus gallinaceus	1	1	**Streptococcus gallinaceus hits**
. Streptococcus plurextorum	1	1	**Streptococcus plurextorum hits**
. Streptococcus saliviloxodontae	1	1	**Streptococcus saliviloxodontae hits**
. Streptococcus himalayensis	1	1	**Streptococcus himalayensis hits**
. Streptococcus koreensis	1	1	**Streptococcus koreensis hits**
. Streptococcus cristatus	1	3	**Streptococcus cristatus hits**
.. Streptococcus cristatus AS 1.3089	1	1	**Streptococcus cristatus AS 1.3089 hits**
.. Streptococcus cristatus ATCC 51100	2	1	**Streptococcus cristatus ATCC 51100 hits**
. Streptococcus parasanguinis ATCC 15912	3	1	**Streptococcus parasanguinis ATCC 15912 hits**
. Streptococcus marmotae	1	1	**Streptococcus marmotae hits**
. Streptococcus porcorum	1	1	**Streptococcus porcorum hits**
. Streptococcus sinensis	1	1	**Streptococcus sinensis hits**
. Streptococcus sanguinis	1	2	**Streptococcus sanguinis hits**
.. Streptococcus sanguinis SK1 = NCTC 7863	2	1	**Streptococcus sanguinis SK1 = NCTC 7863 hits**
. Streptococcus australis	1	1	**Streptococcus australis hits**

. Streptococcus hyointestinalis	1	1	Streptococcus hyointestinalis hits
. Streptococcus vestibularis ATCC 49124	1	1	Streptococcus vestibularis ATCC 49124 hits
. Streptococcus salivarius	1	1	Streptococcus salivarius hits
. Streptococcus penaeicida	1	1	Streptococcus penaeicida hits
. Streptococcus rubneri	1	1	Streptococcus rubneri hits
. Streptococcus urinalis	2	1	Streptococcus urinalis hits
. Streptococcus lutetiensis	3	1	Streptococcus lutetiensis hits
. Streptococcus loxodontisalivarius	1	1	Streptococcus loxodontisalivarius hits
. Streptococcus ilei	1	1	Streptococcus ilei hits
. Streptococcus gordonii	2	1	Streptococcus gordonii hits
. Streptococcus equinus	2	1	Streptococcus equinus hits
. Streptococcus vicugnae	1	1	Streptococcus vicugnae hits
. Streptococcus thermophilus	2	1	Streptococcus thermophilus hits
. Streptococcus uberis	1	1	Streptococcus uberis hits
. Streptococcus ruminantium	1	1	Streptococcus ruminantium hits
. Streptococcus hillyeri	1	1	Streptococcus hillyeri hits
. Streptococcus agalactiae ATCC 13813	3	1	Streptococcus agalactiae ATCC 13813 hits
. Streptococcus oralis	1	4	Streptococcus oralis hits
.. Streptococcus oralis ATCC 35037	2	1	Streptococcus oralis ATCC 35037 hits
.. Streptococcus oralis subsp. tigurinus AZ_3a	1	1	Streptococcus oralis subsp. tigurinus AZ_3a hits
.. Streptococcus oralis subsp. dentisani	1	1	Streptococcus oralis subsp. dentisani hits
. Streptococcus macedonicus	1	1	Streptococcus macedonicus hits
. Streptococcus moroccensis	1	1	Streptococcus moroccensis hits
. Streptococcus acidominimus	1	1	Streptococcus acidominimus hits
. Streptococcus azizii	1	1	Streptococcus azizii hits
. Streptococcus tangierensis	1	1	Streptococcus tangierensis hits
. Streptococcus porcinus	1	1	Streptococcus porcinus hits
. Streptococcus parauberis	1	1	Streptococcus parauberis hits
. Streptococcus lactarius	1	1	Streptococcus lactarius hits
. Streptococcus mitis	3	1	Streptococcus mitis hits
. Streptococcus downii	1	1	Streptococcus downii hits
. Streptococcus iniae	2	1	Streptococcus iniae hits
. Streptococcus vulneris	1	1	Streptococcus vulneris hits
. Streptococcus alactolyticus	1	1	Streptococcus alactolyticus hits
. Streptococcus toyakuensis	1	1	Streptococcus toyakuensis hits
. Streptococcus ictaluri 707-05	1	1	Streptococcus ictaluri 707-05 hits
. Streptococcus panodentis	1	1	Streptococcus panodentis hits
. Streptococcus cameli	1	1	Streptococcus cameli hits
. Streptococcus infantis ATCC 700779	1	1	Streptococcus infantis ATCC 700779 hits
. Streptococcus pasteurianus	1	1	Streptococcus pasteurianus hits
. Streptococcus timonensis	1	1	Streptococcus timonensis hits
. Streptococcus cuniculipharyngis	1	1	Streptococcus cuniculipharyngis hits
. Streptococcus troglodytidis	1	1	Streptococcus troglodytidis hits
. Streptococcus cuniculi	1	1	Streptococcus cuniculi hits
. Streptococcus minor	1	1	Streptococcus minor hits
. Streptococcus varani	1	1	Streptococcus varani hits
. Streptococcus dysgalactiae subsp. equisimilis	1	1	Streptococcus dysgalactiae subsp. equisimilis hits
. Streptococcus xiaochunlingii	1	1	Streptococcus xiaochunlingii hits
. Streptococcus porci	1	1	Streptococcus porci hits
. Streptococcus anginosus group	4	4	
.. Streptococcus constellatus	1	2	Streptococcus constellatus hits
... Streptococcus constellatus subsp. pharyngis	1	1	Streptococcus constellatus subsp. pharyngis hits
.. Streptococcus intermedius	1	1	Streptococcus intermedius hits
.. Streptococcus anginosus SK52 = DSM 20563	1	1	Streptococcus anginosus SK52 = DSM 20563 hits
. Streptococcus pseudoporcinus LQ 940-04	1	1	Streptococcus pseudoporcinus LQ 940-04 hits
. Streptococcus pyogenes	1	1	Streptococcus pyogenes hits
. Streptococcus gwangjuense	1	1	Streptococcus gwangjuense hits
. Streptococcus oricebi	1	1	Streptococcus oricebi hits
. Streptococcus chosunense	1	1	Streptococcus chosunense hits
. Streptococcus oriloxodontae	1	1	Streptococcus oriloxodontae hits
. Streptococcus pneumoniae	1	1	Streptococcus pneumoniae hits
. Streptococcus caledonicus	1	1	Streptococcus caledonicus hits

Follow NCBI

Connect with NLM

National Library of Medicine
8600 Rockville Pike
Bethesda, MD 20894

Web Policies
FOIA
HHS Vulnerability Disclosure

Help
Accessibility
Careers

- NLM
- NIH
- HHS
- USA.gov
